# Perceived difficulty and appropriateness of decision making by General Practitioners: a systematic review of scenario studies

**DOI:** 10.1186/s12913-014-0621-2

**Published:** 2014-11-29

**Authors:** Nicola McCleary, Craig R Ramsay, Jill J Francis, Marion K Campbell, Julia Allan

**Affiliations:** Aberdeen Health Psychology Group & Health Services Research Unit, University of Aberdeen, 2nd Floor Health Sciences Building, Foresterhill, Aberdeen, AB25 2ZD Scotland, UK; Health Services Research Unit, University of Aberdeen, Scotland, UK; School of Health Sciences, City University London, England, UK; Aberdeen Health Psychology Group, University of Aberdeen, Scotland, UK

**Keywords:** Systematic review, Clinical decision making, Decision difficulty, Decision appropriateness, General Practitioner, Primary care physician, Patient scenario, Vignette

## Abstract

**Background:**

Health-care quality in primary care depends largely on the appropriateness of General Practitioners’ (GPs; Primary Care or Family Physicians) decisions, which may be influenced by how difficult they perceive decisions to be. Patient scenarios (clinical or case vignettes) are widely used to investigate GPs’ decision making. This review aimed to identify the extent to which perceived decision difficulty, decision appropriateness, and their relationship have been assessed in scenario studies of GPs’ decision making; identify possible determinants of difficulty and appropriateness; and investigate the relationship between difficulty and appropriateness.

**Methods:**

MEDLINE, EMBASE, PsycINFO, the Cochrane Library and Web of Science were searched for scenario studies of GPs’ decision making. One author completed article screening. Ten percent of titles and abstracts were checked by an independent volunteer, resulting in 91% agreement. Data on decision difficulty and appropriateness were extracted by one author and descriptively synthesised. Chi-squared tests were used to explore associations between decision appropriateness, decision type and decision appropriateness assessment method.

**Results:**

Of 152 included studies, 66 assessed decision appropriateness and five assessed perceived difficulty. While no studies assessed the relationship between perceived difficulty and appropriateness, one study objectively varied the difficulty of the scenarios and assessed the relationship between a measure of objective difficulty and appropriateness. Across 38 studies where calculations were possible, 62% of the decisions were appropriate as defined by the appropriateness standard used. Chi-squared tests identified statistically significant associations between decision appropriateness, decision type and decision appropriateness assessment method. Findings suggested a negative relationship between decision difficulty and appropriateness, while interventions may have the potential to reduce perceived difficulty.

**Conclusions:**

Scenario-based research into GPs’ decisions rarely considers the relationship between perceived decision difficulty and decision appropriateness. The links between these decisional components require further investigation.

**Electronic supplementary material:**

The online version of this article (doi:10.1186/s12913-014-0621-2) contains supplementary material, which is available to authorized users.

## Background

There is extensive evidence demonstrating that patients do not always receive the highest quality care possible [1]. In primary care, General Practitioners (GPs, also known as Primary Care or Family Physicians) are largely responsible for making clinical decisions concerning their patients, so their decisions have a significant impact on health care quality. Decision appropriateness in this context can be defined as the extent to which clinical decisions made by GPs are in accordance with a standard such as an evidence-based clinical guideline (although it is important to note that appropriateness can be conceptualised in many different ways, and guidelines are one of a number of standards that can be used to assess appropriateness). Research into the appropriateness of GPs’ decisions often involves the use of patient scenarios (clinical or case vignettes), where GPs review patient descriptions and simulate the decisions they would make in a real consultation. Decision appropriateness is then assessed by comparing the decisions to an appropriate decision defined by a standard such as a clinical guideline.

Scenario methods can also be used to identify factors influencing the appropriateness of GPs’ decisions. Cognitive psychology theory suggests that a key determinant of a decision outcome is the difficulty of that decision [2,3]: perceived difficulty with decision making is therefore likely to be related to the appropriateness of GPs decisions. There is currently no widely accepted definition of perceived difficulty, but it has been described as being experienced when a decision maker finds it difficult to choose a certain course of action, or when it is unclear which course of action best meets a decision makers’ goals [2]. Perceived difficulty can be assessed by asking decision makers to use a scale to rate the difficulty experienced when making a decision [2].

Although all individuals will have at some point experienced difficulty when making a decision, there has been little scientific study of this concept [2,3]: as such, there is currently no theoretical consensus on the characteristics of a decision that make it difficult [3]. However, there is some indication that the complexity of the decision may be important: increasing complexity may cause difficulty whereby complex decisions involving consideration of many factors are perceived as difficult [3,4]. In focus group discussions based on patient scenarios, GPs described their difficulty with deciding whether to refer older patients for colon cancer screening [5]. One of the main sources of difficulty cited was the number of factors which had to be taken into account [5].

Although it might be intuitively appealing to expect that increased decision difficulty leads to less appropriate decisions, it might also be expected that making difficult decisions involves the use of a more in-depth analytic decision making process and leads to more appropriate decisions. Although one previous systematic review of 30 scenario studies considered the appropriateness of GPs’ decisions [6], to our knowledge there have been no previous reviews of the perceived difficulty of decisions made by GPs, the factors that might influence this, or the relationship between the difficulty and appropriateness of GPs’ decisions. Clearly, many decisions made by GPs are difficult and it may not always possible to make them easier. However, identifying health conditions, decision types, or patient characteristics that are associated with increased difficulty and quantifying the relationship between difficulty and appropriateness could be important for enhancing the knowledge base relating to GPs’ decision making processes, and informing strategies aimed at improving the appropriateness of clinical decisions, and thus patient care.

Consequently, the present review aims to: a) identify the extent to which perceived decision difficulty, decision appropriateness, and the relationship between the two have been assessed in scenario studies of GPs’ decision making; b) identify possible determinants of difficulty and appropriateness within the primary care setting; and c) investigate the relationship between difficulty and appropriateness in the context of GPs’ decision making.

## Methods

This is a systematic review of published studies. This study did not recruit any participants, but involved secondary analysis of papers that are in the public domain: review by an ethics committee was therefore not applicable to this study. This article reflects the relevant components of the PRISMA checklist for the reporting of systematic reviews [7].

### Inclusion criteria

Studies eligible for inclusion used patient scenarios. To ensure study selection was systematic, a definition of patient scenarios was created, similar to that of Veloski and colleagues [8]: a patient scenario is “a brief description of a patient designed to represent an actual primary care consultation”. Studies which stated that scenarios were used but which did not fit this definition were excluded. There were no restrictions on the scenario format or delivery method. Studies using just one scenario were excluded as they could not have compared responses to different scenarios to identify determinants of decision difficulty or appropriateness. Qualitative and think aloud studies were excluded as they do not involve quantitative assessment of decision difficulty or appropriateness in situations which reflect actual consultations. Interview studies which were quantitative in nature (i.e. where an interviewer was present but GPs were given or shown scenarios and asked to give quantitative responses) were included. Studies investigating end of life decision making were excluded because of the many ethical, legal and societal issues inherent in these decisions which are unlikely to be present in the everyday clinical decisions made by GPs working in the community [9].

Participants must have included fully qualified GPs working in community settings. Eligible studies required participants to make a clinical decision (a decision made with respect to patient care [10]) regarding the patients presented in the scenarios. Studies involving non-definitive decisions (for example, where participants rated their willingness to prescribe) were included. Studies which collected any other types of outcome measures (such as estimates of treatment effectiveness) or which did not elicit decisions specifically relating to the patients in the scenarios (for example, where participants rated treatment appropriateness) were excluded, on the basis that participants were not making clinical decisions for patients as they would in actual practice. There were no restrictions on response format.

### Search methods

Electronic searches were carried out in MEDLINE (1946 to week 1 of February 2012) and Embase (1980 to week 6 of 2012) using the OVID interface, and in PsycINFO, the Cochrane Library and Web of Science on February 14th 2012. A search strategy (included in Additional file 1) was designed in MEDLINE and modified accordingly for use in the additional databases. No language restrictions were imposed. GPs are labelled in various ways in countries other than the UK, while many different terms can be used to refer to the use of patient scenarios: this was reflected in our search strategies. The journal *Medical Decision Making* was hand-searched for relevant conference abstracts from 2009-April 2012. The reference lists of included studies were reviewed.

### Data collection

One author (NM) screened titles and abstracts retrieved by electronic searching; 10% were screened by an independent volunteer (Brian Power), resulting in 91% agreement. As an additional check, all authors independently screened a set of the same 10 titles and abstracts. Screening disagreements were resolved by discussion. One author (NM) screened full-text articles using a form (included in Additional file 2) designed using Cochrane guidance [11] and which was piloted by all authors. Study eligibility doubts were resolved by discussion with all authors. One author (NM) extracted data using a form (included in Additional file 3) developed using Cochrane guidance [11] and relevant literature [6,10,12,13] and which was piloted by all authors. Data pertaining to study characteristics, participants, patient scenario construction, outcome measures, and results were extracted and stored electronically.

### Data analysis

Where relevant details were not available in the paper, efforts were made to obtain them by contacting study authors. The number of studies which assessed decision difficulty, decision appropriateness, or the possible relationship between the two was counted. For a study to be categorised as having assessed decision appropriateness, the appropriateness of the decisions made must have been explicitly assessed in reference to some standard, or scenarios must have been designed according to some standard such that the appropriate decision was evident. This review aimed to be inclusive and gather studies which had used a range of approaches to assess decision appropriateness: therefore, we did not restrict appropriateness assessment (for example to national standards), but rather accepted all standards. Studies which discussed guidelines or another standard in relation to their results, but which did not explicitly assess decision appropriateness and provide results for this, were categorised as not having assessed decision appropriateness.

Heterogeneity between studies prevented correlational analysis of factors associated with decision difficulty, so studies were descriptively analysed. However, it was possible to pool the data from the studies which assessed decision appropriateness, by focussing on the number of decisions deemed appropriate. The number of appropriate decisions (defined by the standard used) was calculated by NM where this was possible, as follows:

- Where the overall number of appropriate decisions across all scenarios was reported, this was taken directly from the paper.
- Where the number of appropriate decisions per scenario was reported, these details were taken directly from the paper and summed to create an overall total.
- Where percentages of appropriate decisions were reported, these details were taken directly from the paper and used to calculate the number of appropriate decisions, either overall or per scenario and then summed to create an overall total.

The specific types of decisions made and the decision appropriateness assessment methods used were categorised for each study by one author (NM), and any doubts were resolved by discussion with all authors. To categorise decision type, NM extracted decision details from all studies, grouped them under headings such as prescribing, and headings were then agreed by all authors. All authors agreed on the final categories listed in Table 1. The diagnosis category included diagnostic decisions; the screening or testing category included decisions involving screening, examination or diagnostic test ordering; the treatment or management category included decisions about treatment and further patient management. Since the latter two categories covered a wide range of decisions, these were sub-categorised as indicated in Table 1. The categories of decision appropriateness assessment method in Table 1 reflect the methods used to determine appropriate and inappropriate decisions.

**Table 1:** **Associations between decision appropriateness and decision type and appropriateness assessment standard**

		**No. (%) appropriate decisions**	**No. (%) inappropriate decisions**	**Total**
Decision type*** ^**a**^				
Screening or testing		9133 (81%)	2175 (19%)	11308
Diagnosis		5000 (73%)	1856 (27%)	6856
Treatment or management		19950 (55%)	15991 (45%)	35941
Total		34083 (62%)	20022 (38%)	54105^b^
Decision type sub-group*** ^**a**^				
Screening or testing	Test ordering	9081 (81%)	2105 (19%)	11186
	Examination^c^	52 (43%)	70 (57%)	122
Treatment or management	Prescribing	8000 (60%)	5217 (40%)	13217
	Giving advice	4008 (47%)	4469 (53%)	8477
	Referral	5748 (54%)	4795 (46%)	10543
	Follow-up^c^	105 (43%)	138 (57%)	243
	Appointment-scheduling^c^	11 (52%)	10 (48%)	21
	Treatment other than prescribing^c^	31 (7%)	388 (93%)	419
Total		27036 (61%)	17192 (39%)	44228^d^
Method used for decision appropriateness assessment***				
Guidelines		13284 (55%)	10716 (45%)	24000
Expert panel		15956 (68%)	7432 (32%)	23388
Literature		2653 (71%)	1080 (29%)	3733
Actual diagnosis		48 (23%)	161 (77%)	209
Combination		2339 (56%)	1821 (44%)	4160
Total		34280 (62%)	21210 (38%)	55490^e^

Note: ***p < .001.

^a^Studies included in multiple categories if multiple decisions of different types made.

^b^4561 decisions from four studies excluded as either a) they could not be clearly classified into one category; b) there was insufficient information regarding either the decisions made or the response options given to allow for classification into a category.

^c^Category represents one study.

^d^The 6856 diagnostic decisions were not sub-categorised; 7582 decisions from eight studies excluded due to reasons a) and b) noted above.

^e^3176 decisions from four studies excluded because the standard used was not specified.

Chi-squared tests were used to explore associations between decision appropriateness and decision type and method used to determine decision appropriateness. The decisions within the studies were already categorised for these analyses, based on the categorisation of the overall studies from which the decisions came. Categorising decisions for the analysis was therefore based on the already agreed categories for the studies and so validity checking of the decision categorisations was not thought to be necessary. Data were analysed using SPSS version 20.

## Results

### Study selection

**Figure 1:**
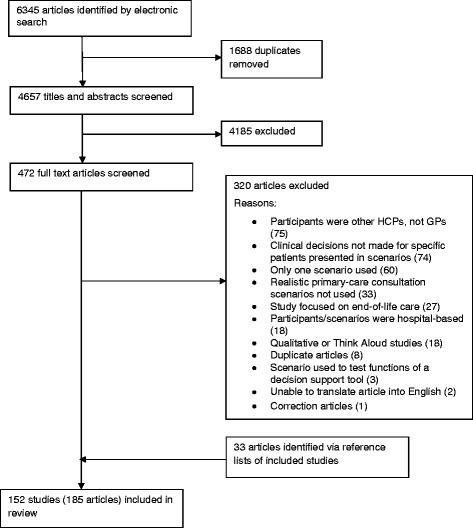
**Flow chart of identification and selection of included studies.** Note: GP = General Practitioner; HCP = Health Care Professional.

The search retrieved 4657 articles, and 185 articles (reporting 152 studies) were included in the review. Full details of the study selection process are provided in Figure 1.

### Characteristics of included studies

The majority of included studies (119) used questionnaires. Twelve of these had additional components: 10 were nested within larger studies (five within randomised controlled trials (RCTs), two within observational studies, one within a pre-post intervention study, one within a before-and-after study, and one within a standardised patient study), and two were factorial experiments. Of the remaining 33 studies, 25 used interviews. Nine of these had additional components: one was nested within an RCT, and eight were factorial experiments. Of the remaining eight studies, two used questionnaire and/or interview surveys, two were crossover experiments which used a balanced block design, one was a simulated cluster RCT, and three were simulated decision making experiments.

Studies were published between 1974 and 2011, with 59% published during or after the year 2000. Thirty seven percent were conducted in the USA, 20% in the UK, 9% in Canada, 5% in Australia and 5% in the Netherlands. The remainder were carried out in different or multiple countries. The studies focussed on a wide range of clinical behaviours, such as diagnosis, test-ordering, prescribing, providing advice, and referral. Further study descriptives are included in Table 2. The majority of studies (74%) used written scenarios which were delivered via paper questionnaires. The rest used scenarios presented on computers or via video. Where specified, various different sources were used to generate scenario content, including clinical experience, real patient data, the literature, and clinical guidelines. A summary of the key features of each study is included in Additional file 4.

**Table 2:** **Key characteristics of 152 included studies**

**Study characteristic**	**Mean (SD)**	**Mode**	**Range**	**Total**
**Number of GPs** ^**a**^	226 (304)	40	4–2155	31252
**Number of scenarios** ^**b**^	18 (38)	4	2–390	2713
**Number of scenarios per participant** ^**c**^	9 (13)	2	1–390	1319
**Number of decisions per participant per scenario** ^**d**^	2 (1)	1	1–12	226

Note: GP = General Practitioner.

Number of studies where these data were missing: ^a^14; ^b^4; ^c^2; ^d^26.

### Decision appropriateness

Sixty-six of the 152 studies assessed decision appropriateness, 43 using guidelines. The number of appropriate decisions could be extracted or calculated for 38 studies: overall, 58666 clinical decisions were made, and 62% were appropriate (mean 58%, SD 24%, range 6% to 100%). Pearson Chi-squared tests found statistically significant associations between decision appropriateness and decision type and decision appropriateness assessment method (Table 1). Across the three analyses, decision appropriateness was

- Highest for screening or testing decisions and lowest for treatment or management decisions
- Highest for test-ordering decisions and lowest for treatment other than prescribing decisions
- Highest when literature was used to assess decision appropriateness, and lowest when actual diagnosis was used

Some studies contributed many decisions (for example, one study [14,15] contributed 20631 decisions), and some categories represented one study. Sensitivity analyses were carried out whereby the analyses were repeated after removing decisions from studies contributing 1000 decisions or more to a category from that category, and categories representing one study. These analyses were not pre-specified. Significant associations between decision appropriateness and study characteristics remained, indicating that the findings are robust.

Table 3 summarises the decision types investigated, and decision appropriateness assessment and analysis methods used in the remaining 28 studies where the number of appropriate decision could not be calculated. This shows that 79% of these studies focussed on treatment or management decisions, with 64% specifically focussed on prescribing. Additionally, 50% of the studies used guidelines only to assess decision appropriateness. The studies used a range of methods to analyse decision appropriateness, such as calculating agreement with the decision appropriateness assessment standard (for example, percentage agreement with an expert panel’s decisions), or calculating quality scores (for example, scoring GPs a point for an appropriate decision then calculating a mean score) (Table 3). For 43% of the studies, proportions of decisions were calculated but the way in which the data were presented prevented inclusion in the Chi-squared analyses.

**Table 3:** **Decision types assessed, decision appropriateness assessment standards used, and decision appropriate analysis methods of 28 studies not included in the Chi-squared analyses**

		**No. studies**
**Decision type** ^**a**^		
Screening or testing		11
Diagnosis^b^		6
Treatment or management		22
**Decision type sub-group** ^**a**^		
Screening or testing	Test ordering	11
	Examination	5
Treatment or management	Prescribing	18
	Giving advice	5
	Referral	8
	Follow-up	4
	Appointment-scheduling	0
	Treatment other than prescribing	5
**Method used for decision appropriateness assessment**		
Guidelines		14
Expert panel		5
Literature		3
Actual diagnosis		1
Combination		5
**Method used to analyse decision appropriateness**		
Calculated agreement with decision appropriateness assessment standard		6
Calculated quality scores		5
Calculated mean proportion of appropriate or non-appropriate decisions		3
Decisions assessed on scales; mean scale ratings compared to decision appropriateness assessment standard		2
Calculated proportion of GPs making different decisions, but:		
• Data presented with those of other HCPs		4
• Unclear specifically which options were appropriate/inappropriate		3
• Focussed only on certain appropriate decisions		2
• Data presented in graphs so cannot extract		1
• Total number analysed not specified		1
• Scenario results amalgamated with results from other questions		1

Note: GP = General Practitioner; HCP = Health Care Professional.

^a^Studies included in multiple categories if multiple decisions of different types made.

^b^The studies focussing on diagnostic decisions were not sub-categorised.

### Decision difficulty and the relationship between decision difficulty and decision appropriateness

Five of the 152 studies assessed the perceived difficulty of the GPs’ decisions [16-20]. One did not report the difficulty data [18], leaving four studies to be analysed. The key features of these studies are summarised in Table 4: further details can be found in the summary of key study features in Additional file 4. As Table 4 indicates, all studies differed greatly in terms of the study type, the health condition investigated, and how the outcome of decision difficulty was measured and the outcome data summarised.

**Table 4:** **Key findings from four studies assessing perceived decision difficulty**

**First author, year, country**	**Study design (interventions assessed)**	**Number of GPs and scenarios**	**Decision made and difficulty assessment**	**Decision results**	**Decision difficulty results**
Bonetti 2005, UK [16]	RCT (A&F & ERM)	Baseline 214 GPs, 10 scenarios	Order lumbar x-ray for back pain (yes or no)	Yes decisions summed per GP	Scores summed per GP
		Follow-up	10-point difficulty scale^a^	Baseline mean scores:	Baseline mean scores:
		152 GPs, 10 scenarios		No A&F 3.59; A&F 3.70	No A&F 40.09; A&F 39.53
				No ERM 3.75; ERM 3.55	No ERM 40.82; ERM 38.77
				Follow-up mean scores:	Follow-up mean scores:
				No A&F 3.47; A&F 3.14^*^	No A&F 41.16; A&F 38.61^*^
				No ERM 3.60; ERM 3.01^*^	No ERM 40.31; ERM 39.46
Carroll 2011, Canada [17]	RCT (KT)	Baseline 80 GPs, 10 scenarios	Refer women with different HBOC risk (yes or no)	Appropriate decisions summed per GP	Scores summed per GP
		Follow-up	7-point difficulty scale^a^	Baseline mean scores:	Baseline mean scores:
		80 GPs, 10 scenarios		Control 7.1; KT 6.5	Control: 30.7; KT: 32.8
				Follow-up^b^ mean scores:	Follow-up^b^ mean scores:
				Control 6.4; KT 7.8^*^	Control: 33.4; KT: 29.7
Short 2003, UK [20]	Before & after (CDSS)	15 GPs, 10 scenarios	Prescribe aspirin for stroke (15 point scale^c^)	Across 9 scenarios where prescribing appropriate, overall shift 116 points towards prescribing	Mean scale scores:
			5-point difficulty scale^d,e^		Before = 2.7; After = 3.1
Lynggaard 2006, Denmark [19]	Questionnaire^f^	55 GPs, 5 scenarios	Prescribe for hypertension	% GPs prescribing per scenario:	% ‘easy’ decisions per scenario:
			3-point difficulty scale^g^	96%; 85%; 96%; 56%; 63%	83%; 67%; 80%; 50%; 50%

Note: A&F = audit & feedback; CDSS = computerised decision support system; ERM = educational reminder messages; HBOC = hereditary breast & ovarian cancer; KT = knowledge translation; RCT = randomised controlled trial.

^*^p < .05.

^a^Not at all difficult to extremely difficult.

^b^Adjusted for baseline imbalance between the intervention and control group.

^c^Yes aspirin to no aspirin, with unsure at mid-point.

^d^Strongly disagree to strongly agree prescribing decisions easy to make (assessed in relation to decisions overall, not per scenario).

^e^Both scales adapted from scales developed by the Ottawa Hospital Research Institute.

^f^Adapted from Hamilton-Craig and colleagues [21].

^g^Hard, moderate, easy.

Efforts were made to contact authors of all four studies to obtain scenario content, and the scenarios used by Lynggaard and Strandgaard and by Short and colleagues were obtained. The number of pieces of information in these scenarios were counted by one author (NM) and used as an indicator of complexity. The scenarios used by Lynggaard and Strandgaard, the number of pieces of information, the percentage of GPs who prescribed and who perceived the decision as easy are include in Table 5. As Table 5 shows, the two scenarios containing eight pieces of information were perceived as easy by a greater proportion of GPs than the three scenarios containing nine pieces. Therefore, clinical situations containing more information were more often perceived as difficult. However, it is not clear whether this is due to either the number of pieces of information per se, what these pieces of information were, how they have been combined in the scenarios, another reason we have not considered, or whether this is simply a chance finding. This could not be explored further using Short and colleagues’ scenarios since difficulty was not assessed per scenario.

**Table 5:** **Scenario details and percentage of GPs prescribing and who perceived the prescribing decision as easy for the scenarios used by Lynggaard and Strandgaard** [19]

**Scenarios**	**No. pieces of information**	**Pieces of information**	**% GPs prescribing**	**% GPs who perceived decision as easy**
1. Mrs Louise Pastor, a 74 year old woman, non-diabetic who smokes 20 cigarettes a day. Her blood tests reveal a total cholesterol of 4.4, an HDL of 1.4 (ratio of 4), and she has a blood pressure of 180/84.	8	Gender, age, diabetes status, smoking status, total cholesterol, high density lipoprotein cholesterol, cholesterol ratio, blood pressure	96%	83%
2. Miss Alexandra Fleming is a 52 year old mycologist. She is not diabetic, and an avowed non-smoker. On her last visit she had a total cholesterol of 7.2, hdl 1.2 (ratio of 6), and a blood pressure of 150/95.	9	As above plus occupation	85%	67%
3. Mr Samuel Vise, is a 50 year old man. He has Non-insulin dependent diabetes mellitus, is a non-smoker, with a total cholesterol of 6.6, an hdl of 1.1 (ratio of 6) and a blood pressure of 162/92.	8	As above	96%	80%
4. Mrs Marie Curry - 58 year old French woman with Non Insulin-dependent diabetes mellitus, who smokes 20 cigarettes a day, has a total cholesterol of 9.0 and hdl 1.3 (ratio of 6) and a demonstrated blood pressure of 150/98.	9	As above plus nationality	56%	50%
5. Carl “Rocky” Tansky is a 35 year old boxer. He is a non-diabetic whose coach will not allow him to smoke, with a total cholesterol of 5.0 and an HDL of 1.0 (ratio of 5). He has a blood pressure 158/96 when not in the ring.	9	As above plus occupation	63%	50%

Note: Scenarios reproduced with the permission of the corresponding author of the original article from which the scenarios were adapted [21].

One study, by Kostopoulou and colleagues, explicitly investigated the relationship between decision difficulty and decision appropriateness [22]. However, perceived decision difficulty was not assessed: various sources were used to derive 10 factors which may cause diagnostic difficulty, and the presence of these was varied across scenarios [22]. This study therefore focussed on objective difficulty, which can be defined as an independent assessment of the difficulty of a decision task. Participants (63 GPs and 21 residents) made diagnostic and management decisions, and decision appropriateness was assessed using expert panel diagnoses and guideline recommendations [22]. The correlation between decision difficulty and appropriateness for the diagnostic decisions was reported. There was a significant negative correlation: as the number of difficulty factors increased, the number of appropriate diagnostic decisions decreased [22].

The four previous studies which assessed perceived decision difficulty provide some support for there being a negative relationship between perceived difficulty and appropriateness, although the relationship was not explicitly assessed:

- Bonetti and colleagues conducted an RCT investigating the effectiveness of audit and feedback and educational reminder messages in improving lumbar spine x-ray ordering decisions for back pain [16]. There was no effect of the educational reminders on difficulty. However, the GPs who received audit and feedback found the post-intervention decisions significantly less difficult than those who did not [16] (Table 4). There was a significant correlation between difficulty and post-intervention decisions [16]: as the difficulty score increased, the number of decisions to order an x-ray (which were mostly inappropriate) increased. However, difficulty did not enter a regression model predicting decisions [16] (instead, decisions were predicted by attitude, subjective norm, and perceived behavioural control, from the Theory of Planned Behaviour [23]).
- Carroll and colleagues conducted an RCT investigating the effectiveness of a knowledge translation intervention in improving referral decisions for women with different cancer risks [17]. The authors found that a significantly greater number of appropriate decisions were made in the intervention group than the control group at post-intervention and, as indicated previously, that difficulty was lower [17] (Table 4).
- Short and colleagues conducted a before-and-after study investigating the effectiveness of a computerised decision support tool in improving aspirin prescribing decisions for stroke patients with complicating co-morbidity [20]. After the intervention the authors found stronger agreement that the decisions were easy and an overall shift towards prescribing (which was mostly appropriate) [20] (Table 4).
- Lynggaard and Strandgaard conducted a questionnaire survey investigating decisions to start treatment for mild to moderate hypertension and commented that according to the New Zealand Core Services Committee Guidelines, it was appropriate to prescribe for scenarios 1–3, and not to prescribe for scenarios 4 and 5 in their study [19]. The percentage of appropriate decisions per scenario was 96%, 85%, 96%, 44%, and 37% respectively (Tables 4 and 5). Comparing this to the percentages of decisions considered easy (83%, 67%, 80%, 50% and 50% respectively) shows that when fewer GPs made an appropriate decision, more GPs considered that decision difficult.

## Discussion

Of 152 scenario studies which investigated GPs’ clinical decisions, 66 assessed decision appropriateness, 5 assessed perceived decision difficulty, and one assessed the relationship between objective difficulty and appropriateness. Therefore, the appropriateness of GPs’ decision making has been assessed to a much greater extent than difficulty, while the potentially important relationship between difficulty and appropriateness has rarely been studied in this context. This agrees with the wider psychological literature on decision making which, as stated in the introduction, has rarely investigated decision difficulty and its relationship with decision outcomes [2,3].

Overall, 62% of the decisions made across studies were appropriate, which is consistent with the hypothesis that there are deficiencies in clinical decision making. However, the range of appropriate decisions across included studies was considerably large (6% to 100%): it is therefore unclear to what extent this overall figure relates to real practice. The wide range in appropriateness found may reflect a multitude of factors: for example, the different patient groups and decision types studied in the included papers, the varying levels of detail provided in the scenarios, or the different methods used to assess decision appropriateness. It is often argued that scenarios have limited ecological validity (the extent to which an aspect of the real world is represented, in this case the real world of clinical practice) [24]. For example, scenarios are often missing certain components of real consultations, such as information gathered from non-verbal cues, although video scenarios can help alleviate some of these problems. We cannot comment on the ecological validity of the included scenarios, as assessing this was beyond scope of this review. However, many studies show that there is wide variation in the quality of primary care delivered in practices across the UK [25-28], and our results agree with this.

Most decisions concerned treatment or management, most of which concerned prescribing. This is also true for the 28 studies where the number of appropriate decisions could not be calculated. Many actual consultations involve prescribing: for example, each GP in Scotland issues an average of 70 prescriptions a day [29]. Prescribing is therefore one of the most common decisions made by GPs, and so it is not surprising that this decision type would be frequently studied using scenarios. Only 60% of the prescribing decisions in the Chi-squared analyses were appropriate, and further evidence suggests that inappropriate prescribing does occur in primary care [30-34]. This indicates that decision appropriateness is suboptimal for the decisions most frequently made by GPs: further work is required to improve these decisions.

Only 47% of the advice-giving decisions were appropriate, which is again suboptimal. The literature on prescribing for upper respiratory tract infections (one of the most common primary care consultation types [32,35]) suggests that GPs prescribe rather than provide appropriate advice because they assume this will save time [36,37]. This suggests that GPs’ decisions are not solely influenced by the relevant evidence base: practical constraints, such as physician time, effort, and reward, are also important. Indeed, this agrees with previous quantitative and qualitative research indicating that ‘non-clinical’ or contextual factors, such as the time available, workload, years of experience, and patient demand (or GPs’ perceptions of demand) do influence the decisions that GPs make [38-46].

Both ours and a previous review [6] found that decision appropriateness is typically assessed by comparing decisions with guidelines. This seems logical - guidelines were created to improve and standardise practice [47], so could be considered a highly appropriate standard for evaluating practice. In addition, our review found that conclusions regarding decision appropriateness differ depending on how appropriateness is assessed: in comparison to guidelines, studies using other methods potentially overestimate decision appropriateness. However, guidelines may not always reflect appropriate decisions in the real world. As most GPs are well aware, guideline recommendations usually apply to the ‘average patient’ and it is the responsibility of the clinician to use the recommendation in conjunction with their knowledge of the patient to form a clinical opinion, and then discuss options with the patient. However, GPs cannot use this strategy in scenario studies: when assessing decision appropriateness using guidelines, the decision made is compared to the guideline recommendation and rated as appropriate or inappropriate. The results of this review further emphasise a point made in the introduction, namely that there are numerous ways in which health care quality can be conceptualised: it is possible that these other methods allow for broader definitions of appropriate decisions. In the studies using expert panels, the panels were convened to consider the specific scenarios used, and therefore made recommendations based on these specific clinical situations. This may have contributed to the difference in percentage of appropriate decisions. In future studies, it may be advantageous to use a combination of methods.

Related to this, the fact that we could not include 28 of the studies that assessed decision appropriateness in the Chi-squared analyses indicates not only that there are different ways of conceptualising decision appropriateness, but also that there are different ways of analysing and reporting the results of studies that investigate this. Aside from the 12 studies where data could not be included in our analyses due to the format in which the data were presented, the most common analysis methods in these studies involved calculating agreement with an appropriateness standard such as an expert panel, or calculating quality scores. This highlights the difficulty with synthesising scenario data: there are no widely accepted reporting standards, therefore a variety of methods are used.

As mentioned in the introduction, there has been little research into difficulty and its potential determinants in the psychological literature: the present review shows that this is also the case in clinical decision-making scenario literature. This is an important gap in the research: there could be many factors which contribute to increased difficulty with real clinical decisions. These could be patient-related factors such as the nature of the complaint, the presence of contradictory symptoms or signs, the severity of problem, or whether the patient requests a particular treatment; GP or practice-related factors such as the number of years qualified, the practice case mix, or typical workloads; or other factors such as the number of possible treatment or management options that can be selected and their potential outcomes, or the time of day or day of the week. Further studies are needed which investigate or manipulate these factors and compare difficulty across situations to identify factors that may influence difficulty.

The few studies identified which had assessed perceived decision difficulty did so for a variety of different consultation types and health problems, and measured and summarised decision difficulty in a variety of ways. Despite this variability, the three intervention studies indicated that interventions aimed at improving GPs’ decisions have the potential to reduce perceived difficulty [16,17,20]. Only one study provided difficulty scores per scenario [19]: in the other studies, perceived difficulty was assessed for the set of scenarios as a whole [20], or difficulty scores were summed across scenarios [16,17] (see Table 4). This is a key finding: studies where difficulty scores are assessed per scenario afford the opportunity to investigate which specific scenario factors influence the perceived difficulty scores. As our review has found, these types of studies in particular are lacking.

Although no studies explicitly assessed the relationship between perceived difficulty and decision appropriateness, one study identified a significant negative association between objective difficulty and appropriateness [22]. In another study [19], the more information the scenarios contained, the greater the proportion of participants who perceived scenarios as difficult, supporting the view that as complexity increases, difficulty increases [3-5]. Here, complexity could be regarded as an objective measure of difficulty. Importantly, as more GPs perceived scenarios as difficult and complexity increased, fewer GPs made appropriate decisions. However, it is important to note that it is not clear that increasing the amount of information leads to increased perceived difficulty. This result could be due to the nature or relevance of the information in the scenarios or could be a chance finding. Further research is therefore required before any firm conclusions can be drawn. Although the relationship between perceived difficulty and appropriateness was not explicitly assessed in the four studies which assessed perceived difficulty, the results provide some support for there being a negative relationship: where decision difficulty was greater, it was generally the case that fewer appropriate decisions were made [16,17,19,20].

### Recommendations for future research

On the basis of these results, hypotheses can be generated regarding the relationships between objective decision difficulty, perceived decision difficulty, and decision appropriateness, in the context of GPs’ clinical decision making. Specifically, our findings are in accordance with the hypotheses that a) as objective difficulty increases, perceived difficulty increases; b) as objective difficulty increases, appropriateness decreases; and c) as perceived difficulty increases, appropriateness decreases.

Further robustly-designed studies are necessary to test these hypotheses, especially given that studies in this review have indicated that interventions may have the potential to reduce perceived decision difficulty. This further research should involve assessment of perceived difficulty, as well as objective measurement of the difficulty of the scenarios used, perhaps using panels of GPs. Objective difficulty could also be manipulated in the manner of Kostopoulou and colleagues. It is also important going forward for researchers to create scenarios in a systematic fashion such that specific aspects can be related to difficulty and appropriateness, perhaps using regression techniques: this would help identify determinants of difficulty and appropriateness. Finally, future studies could use both guidelines and expert panels to assess appropriateness in a comprehensive manner.

We suggested in the introduction that a decision perceived as difficult may instigate a more effortful decision making process than decisions perceived as easy, leading to a more appropriate decision, The results of this review are not in line with this hypothesis, However, the negative relationships suggested by the review results may be attenuated in scenario studies as opposed to studies of real decisions, as participants in scenario studies may see this as a good opportunity to instigate an effortful decision process. Investigation of real decisions is therefore also important, especially given the context of ever-growing complexity in primary care: GPs are expected to be aware of an increasing number of guideline recommendations, and to incorporate patient preferences into their decision strategies [48]. Multi-morbidity also increases complexity: this can result in competing issues, and can make following guideline recommendations, which are typically written for a single morbidity, increasingly complicated.

### Strengths and limitations

The comprehensive nature of the search allowed studies from a range of countries to be included in this review, increasing the generalisability of our findings. Our study also included data from a wide range of GPs’ decisions, allowing results to be applicable to the range of common decisions faced in primary care. In addition, a large number of scenarios were included (data from over 58000 individual clinical decisions) increasing the power of our study to detect clinically important differences, should they exist. However, as previously indicated, issues with the ecological validity of scenarios mean that the extent to which our findings are generalizable to real practice in unclear. Nevertheless, our results agree with the multitude of studies showing that there is variation in quality of care.

There are also a number of limitations. Since participants in these studies responded to multiple scenarios, their decisions may not be independent: this was not adjusted for in the Chi-squared analyses. The significant associations found were highly statistically significant, which occurs when large sample sizes are used: this discussion has, therefore, focussed on the trends in the data. Although a Chi-squared analysis is limited, we considered it the optimal method of analysing our results as of all the analysis methods considered, it was the method that enabled data from the greatest proportion of the studies which assessed decision appropriateness to be analysed. These analyses did not include two further factors which may be associated with decision appropriateness: patient group, and whether appropriate decisions involved the GPs performing an action (e.g. ordering a necessary test) or not performing an action (e.g. not prescribing unnecessary antibiotics). These were excluded due to difficulties with study categorisation. As discussed, it was not possible to calculate numbers of appropriate decisions for 28 of the 66 studies which assessed decision appropriateness, so we were unable to aggregate all the data. This highlights an important issue with respect to outcome reporting in scenario research: many different methods are used, preventing data synthesis. It is important that researchers, reviewers and editors strive for consistency.

Most of the analyses were carried out by one reviewer: however, issues were resolved by discussion with the whole author team. Another issue arose when determining whether the same studies were being reported in certain articles. Articles were treated as reporting the same study when this was clear. However, we acknowledge that these difficulties may have resulted in double-counting of some studies. In all but two instances where there was a concern, only one of the papers had decision appropriateness data that were used in the Chi-squared analyses. These two instances involved the diagnostic decision data for depression from the studies by Freund and colleagues (121 appropriate of 128 decisions) and Frayne and colleagues (155 appropriate of 243 decisions), and the diagnostic, prescribing and test-ordering data for Coronary Heart Disease from the studies by Arber and colleagues (873 appropriate of total 1536 decisions) and Shackelton-Piccolo and colleagues (942 appropriate of total 1835 decisions). It is also possible that we may not have retrieved all relevant studies: however, we attempted to combat this by creating as comprehensive a search strategy as possible.

The final issue concerns the validity of patient scenarios as a proxy method of studying GPs’ decision making. Evidence from rigorous studies carried out by Peabody and colleagues suggests that scenarios are a valid proxy measure of clinical behaviour [49,50], but this is not conclusive [10,51]. This may be because there is no standardised method for developing valid scenarios, resulting in wide variation in how rigorously scenarios are validated before use. However, scenario studies are arguably less ethically challenging than methods requiring direct observation of consultations, and considerably less resource intensive. Therefore, it seems sensible for researchers to carry out further work to explore the relationship between difficulty and appropriateness firstly using valid scenarios designed to reflect real practice, such that the results of scenario studies are useful for informing practice, and then in real practice, where studies are considerably more resource-intensive and complex. In summary, this review has certain limitations which must be acknowledged when interpreting the results. It is unclear how representative of real practice the scenarios used were, and the Chi-squared analyses were limited. Therefore, the extent to which our findings are generalizable to real practice in unclear. However, our results agree with studies of real practice showing that quality of care varies, and have allowed for the generation of specific hypotheses regarding relationships between decisional components, which researchers may wish to test.

## Conclusions

This review has found that in scenario studies, the appropriateness of GPs’ decision making has been assessed to a much greater extent than perceived difficulty, which is not routinely assessed. On average, 62% of the decisions made across studies were judged appropriate by some external assessment method (ranging from 81% for screening or testing decisions to only 55% for treatment or management decisions). The results also indicate that variation in difficulty might be important for decision appropriateness: specifically, greater decision difficulty may result in lower likelihood of an appropriate clinical decision being made. However, intervention studies indicate that perceived decision difficulty is modifiable and so it may be possible to improve decision appropriateness through attempts to reduce decision difficulty, although it is important to firstly establish the determinants of difficulty. Scenario-based research into GPs’ decisions rarely considers the relationship between decision difficulty and appropriateness: more research is needed to identify the specific factors which influence decision difficulty and appropriateness, to specify the relationship between decision difficulty and appropriateness, and ultimately to improve the appropriateness of clinical decisions made by GPs.

## Additional files

Additional file 1:
**Search Strategies.**
Additional file 2:
**Screening Form.**
Additional file 3:
**Data Extraction Form.**
Additional file 4:
**Key Features of Included Studies.**

## Abbreviations

A&F: Audit & feedback
CDSS: Computerised decision support system
ERM: Educational reminder messages
GP: General Practitioner
HBOC: Hereditary breast & ovarian cancer
HCP: Health Care Professional
KT: Knowledge translation
RCT: Randomised controlled trial
