# Supplementary material for: Perceived difficulty and appropriateness of decision making by General Practitioners: a systematic review of scenario studies
Source: BMC Health Serv Res. 2014 Nov 29;14:621. doi: 10.1186/s12913-014-0621-2 (PMC4258016; doi:10.1186/s12913-014-0621-2)
Supplement: Additional file 1: — Search Strategies. [file 12913_2014_621_MOESM1_ESM.doc]

**Additional File 1: Search Strategies**

*MEDLINE and Embase:*

1. exp Physicians/ use mesz
2. exp Physician/ use emez
3. (general practitioner? or medical practitioner? or physician? or clinician? or doctor?).tw.
4. (gp or gps).tw.
5. (health care provider? or healthcare provider?).tw.
6. (health care practitioner? or healthcare practitioner?).tw.
7. (health care professional? or healthcare professional?).tw.
8. Primary Health Care/
9. primary medical care/ use emez
10. General Practice/
11. Family Practice/ use mesz
12. (primary care or primary medical care or primary health care or primary healthcare).tw.
13. general practice.tw.
14. family practice.tw.
15. or/1-14
16. Decision Making/
17. exp Clinical Decision Making/ use emez
18. exp Medical Decision Making/ use emez
19. Judgment/ use mesz
20. Problem solving/
21. (decision making or judg?ment or problem solving or reasoning).tw.
22. ((clinical or medical) adj3 (decision making or decision? or judg?ment? or problem solving or reasoning)).tw.
23. ((research or measure or assess) adj3 (decision? or judg?ment?)).tw.
24. ((research or measure or assess) adj3 (clinical or medical) adj3 (decision? or judg?ment?)).tw.
25. ((treat$ or therap$) adj3 (decision? or judg?ment? or strategy or strategies)).tw.
26. ((diagnos$ or management) adj3 (decision? or judg?ment? or strategy or strategies)).tw.
27. or/16-26
28. vignette/ use emez
29. simulation/ use emez
30. (vignette? or scenario? or simulation?).tw.
31. ((patient or clinical or case or written) adj3 (vignette? or scenario? or description? or simulation?)).tw.
32. (hypothetical patient? or hypothetical case? or hypothetical scenario?).tw.
33. ((behavio?r? or behavioural or case? or patient?) adj3 (simulate or simulation)).tw.
34. ((proxy or measure) adj3 clinical behavio?r?).tw.
35. ((proxy or measure) adj3 medical behavio?r?).tw.
36. patient management problem?.tw.
37. case history questionnaire?.tw.
38. paper case?.tw.
39. (case history or case histories).tw.
40. judg?ment case?.tw.
41. or/28-40
42. 15 and 27 and 41
43. (review or letter or comment or editorial).pt.
44. 42 not 43
45. (animals/ or nonhuman/) not humans/
46. 44 not 45
47. remove duplicates from 46

*Cochrane Library:*

1. MeSH descriptor Physicians explode all trees
2. physician or "general practitioner" or clinician or "medical practitioner" or doctor
3. gp or gps
4. "health-care practitioner" or "healthcare practitioner"
5. "health-care provider" or "healthcare provider"
6. "health-care professional" or "healthcare professional"
7. MeSH descriptor Primary Health Care, this term only
8. MeSH descriptor General Practice explode all trees
9. "primary care" or "primary medical care" or "primary health-care" or "primary healthcare"
10. "general practice" or "family practice"
11. (#1 OR #2 OR #3 OR #4 OR #5 OR #6 OR #7 OR #8 OR #9 OR #10)
12. MeSH descriptor Decision Making explode all trees
13. MeSH descriptor Judgment, this term only
14. MeSH descriptor Problem Solving, this term only
15. "decision making" or judgement or judgment
16. "problem solving" or reasoning
17. (clinical or medical) NEAR/3 ("decision making" or decision or judgement or judgment or "problem solving" or reasoning)
18. (research or measure or assess) NEAR/3 (decision or judgement or judgment)
19. (research or measure or assess) NEAR/3 (clinical or medical) NEAR/3 (decision or judgement or judgment)
20. (treat* or therap*) NEAR/3 (decision or judgement or judgment or strategy)
21. (diagnos* or management) NEAR/3 (decision or judgement or judgment or strategy)
22. (#12 OR #13 OR #14 OR #15 OR #16 OR #17 OR #18 OR #19 OR #20 OR #21)
23. MeSH descriptor Patient Simulation, this term only
24. vignette or scenario or simulation
25. (patient or clinical or case or written) NEAR/3 (vignette or scenario or description or simulation)
26. "hypothetical patient" or "hypothetical case" or "hypothetical scenario"
27. (behavior or behaviour or behavioral or behavioural or case or patient) NEAR/3 (simulate or simulation)
28. "case history questionnaire"
29. "paper case" or "case history" or "judgement case" or "judgment case"
30. (#23 OR #24 OR #25 OR #26 OR #27 OR #28 OR #29)
31. (#11 AND #22 AND #30)

*PsycINFO:*

1. DE "Physicians" OR DE "Family Physicians" OR DE "General Practitioners" OR DE "Gynecologists" OR DE "Internists" OR DE "Neurologists" OR DE "Obstetricians" OR DE "Pathologists" OR DE "Pediatricians" OR DE "Psychiatrists" OR DE "Surgeons"
2. DE "Clinicians"
3. TX general practitioner OR TX gp OR TX medical practitioner OR TX physician OR TX clinician OR TX doctor
4. TX health-care provider OR TX healthcare provider
5. TX health-care practitioner OR TX healthcare practitioner
6. TX health-care professional OR TX healthcare professional
7. DE "Primary Health Care"
8. TX primary care OR TX primary medical care OR TX primary health-care OR TX primary healthcare
9. TX general practice OR TX family practice
10. S1 or S2 or S3 or S4 or S5 or S6 or S7 or S8 or S9
11. DE "Decision Making" OR DE "Choice Behavior"
12. DE "Judgment" OR DE "Clinical Judgment (Not Diagnosis)" OR DE "Probability Judgment"
13. DE "Problem Solving" OR DE "Cognitive Hypothesis Testing" OR DE "Heuristics"
14. DE "Reasoning" OR DE "Case Based Reasoning"
15. TX decision making OR TX judg#ment OR TX problem solving OR TX reasoning
16. TX (clinical or medical) N3 (decision making or decision or judg#ment or problem solving or reasoning)
17. TX (research or measure or assess) N3 (decision or judg#ment)
18. TX (research or measure or assess) N3 (clinical or medical) N3 (decision or judg#ment)
19. TX (treat* or therap*) N3 (decision or judg#ment or strategy)
20. TX (diagnos* or management) N3 (decision or judg#ment or strategy)
21. S11 or S12 or S13 or S14 or S15 or S16 or S17 or S18 or S19 or S20
22. TX vignette OR TX scenario OR TX simulation
23. TX (patient or clinical or case or written) N3 (vignette or scenario or description or simulation)
24. TX hypothetical patient OR TX hypothetical case OR TX hypothetical scenario
25. TX (behavio#r or behavioural or case or patient) N3 (simulate or simulation)
26. TX patient management problem OR TX case history questionnaire
27. TX paper case OR TX case history OR TX judg#ment case
28. TX written analogy
29. TX (proxy or measure) N3 (clinical behavio#r)
30. TX (proxy or measure) N3 (medical behavio#r)
31. S22 or S23 or S24 or S25 or S26 or S27 or S28 or S29 or S30
32. S10 and S21 and S31

*Web of Science:*

1. TS= (physician$ or clinician$ or "general practitioner" or "general practitioners" or "medical practitioner" or "medical practitioners" or doctor$)
2. TS= (gp or gps)
3. TS= ("health-care practitioner" or "health-care practitioners" or "healthcare practitioner" or "healthcare practitioners")
4. TS= ("health-care provider" or "health-care providers" or "healthcare provider" or "healthcare providers")
5. TS= ("health-care professional" or "health-care professionals" or "healthcare professional" or "healthcare professionals")
6. TS= ("primary care" or "primary medical care" or "primary health-care" or "primary healthcare")
7. TS= ("general practice" or "family practice")
8. #7 OR #6 OR #5 OR #4 OR #3 OR #2 OR #1
9. TS= ("decision making" or "medical decision making" or "clinical decision making")
10. TS= (judg$ment or "problem solving" or reasoning)
11. TS= ((clinical or medical) NEAR/3 (decision$ or judg$ment$ or "problem solving" or reasoning))
12. TS= ((research or measure or assess) NEAR/3 (decision$ or judg$ment$))
13. TS= ((research or measure or assess) NEAR/3 (clinical or medical) NEAR/3 (decision$ or judg$ment$))
14. TS= ((treat* or therap* or diagnos* or management) NEAR/3 (decision$ or judg$ment$ or strategy or strategies))
15. #14 OR #13 OR #12 OR #11 OR #10 OR #9
16. TS= ((patient or clinical or case or written) NEAR/3 (vignette$ or scenario$ or simulation$ or description$))
17. TS= ("hypothetical patient" or "hypothetical case" or "hypothetical scenario" or "hypothetical patients" or "hypothetical cases" or "hypothetical scenarios")
18. TS= ("patient management problem" or "patient management problems")
19. TS= ("case history questionnaire" or "case history questionnaires")
20. TS= ("paper case" or "case history" or "judg$ment case" or "paper cases" or "case histories" or "judg$ment cases")
21. TS= (vignette$ or scenario$)
22. TS= ((behavio$r* or case$ or patient$) NEAR/3 (simulate or simulation))
23. #22 OR #21 OR #20 OR #19 OR #18 OR #17 OR #16
24. #23 AND #15 AND #8
