# Supplementary material for: Perceived difficulty and appropriateness of decision making by General Practitioners: a systematic review of scenario studies
Source: BMC Health Serv Res. 2014 Nov 29;14:621. doi: 10.1186/s12913-014-0621-2 (PMC4258016; doi:10.1186/s12913-014-0621-2)
Supplement: Additional file 2: — Screening Form. [file 12913_2014_621_MOESM2_ESM.doc]

**Additional File 2: Screening Form**

Screening Form version 3 (05/04/12)

| Reviewer initials |  | | Date |  |
| --- | --- | --- | --- | --- |
| First author |  | | | |
| Journal |  | | | |
| Citation information |  | Refworks ID | |  |

**Study Eligibility**

Please circle your response to the following questions:

| Are multiple patient scenarios used[[1]](#footnote-2)? | Yes/no |
| --- | --- |
| Do participants include fully qualified General Practitioners (GPs)[[2]](#footnote-3)? | Yes/no |
| Are the scenarios used to elicit clinical decisions[[3]](#footnote-4) from the participants? | Yes/no |

| If any responses are no, do not proceed to data extraction: record reason(s) for exclusion |
| --- |
|  |
| Further comments |
|  |

References

Hrisos S, Eccles MP, Francis JJ, Dickinson HO, Kaner EF, Beyer F, Johnston M: **Are there valid proxy measures of clinical behaviour? A systematic review.** Implementation Science 2009, **4**:37.

Veloski J, Tai S, Evans AS, Nash DB: **Clinical vignette-based surveys: A tool for assessing physician practice variation.** *Am J Med Qual* 2005, **20**(3):151-157.

1. Patient scenarios are descriptions of patients which comprise a brief case history of a hypothetical patient (Veloski et al, 2005). The scenarios can be delivered on paper or electronically via a computer. [↑](#footnote-ref-2)
2. GPs can also be referred to as Primary Care or Family Physicians. [↑](#footnote-ref-3)
3. Clinical decisions are decisions that a healthcare practitioner makes with respect to a patient in their care (Hrisos et al, 2009): for GPs, this will take the form of a diagnostic, treatment or other management decision, or a combination of these. [↑](#footnote-ref-4)
