# Supplementary material for: Perceived difficulty and appropriateness of decision making by General Practitioners: a systematic review of scenario studies
Source: BMC Health Serv Res. 2014 Nov 29;14:621. doi: 10.1186/s12913-014-0621-2 (PMC4258016; doi:10.1186/s12913-014-0621-2)
Supplement: Additional file 3: — Data Extraction Form. [file 12913_2014_621_MOESM3_ESM.doc]

**Additional File 3: Data Extraction Form**

Data Extraction Form Version 6 (25/06/12)

| Reviewer Initials |  | Date |  |
| --- | --- | --- | --- |
| Study Title |  | | |
| First author |  | | |
| Journal |  | | |
| Citation information |  | Refworks ID |  |
| Country(ies) where study conducted |  | | |

| Study aim(s); objective(s); research question(s); hypothesis(es) |
| --- |
|  |

| Study design | Postal questionnaire survey/ RCT/before and after study/interrupted time series study/other (please specify): |
| --- | --- |
| Year survey distributed (or study duration if applicable) |  |

Participants

| Inclusion/exclusion criteria | | |
| --- | --- | --- |
|  | | |
| Recruitment method | Whole population approached/random sample/other (please specify): | |
| Sampling frame | | |
|  | | |
| Number GPs approached |  | |
| Number GPs recruited |  | |
| Number GPs analysed |  | |
| Number approached |  | |
| Number recruited |  | |
| Number analysed |  | |
| If questionnaire: strategy for maximising response | | |
|  | | |
| If questionnaire: does paper indicate that non-response/ selection bias was assessed? | | Yes/no |
| If yes, provide details | | |
|  | | |
| Descriptive information collected and values | | |
|  | | |

Patient scenarios

| Term(s) used to label the use of scenarios within the paper |  | | | | |
| --- | --- | --- | --- | --- | --- |
| Total number of scenarios used |  | Total number of scenarios provided to each participant | | |  |
| Reason if different |  | | | | |
| Clinical decision(s) investigated | | | | | |
|  | | | | | |
| Source of scenario content | | | | | |
| Guidelines/ previous research/ data from real patients/ clinical experience of authors/ scenarios from a previous study used (provide citation details)/ other (please specify)/ unclear  Brief description of sources: | | | | | |
| Method used to combine scenario content | | | | | |
| full factorial approach/ fractional factorial approach/ content varied independently/ other (please specify)/ unclear  Brief description of methods: | | | | | |
| If factorial/independent variation: provide details of factors and levels of each factor | | | | | |
|  | | | | | |
| Provide brief scenario description and other important details included within scenarios (if not clear from previous questions) | | | | | |
|  | | | | | |
| Does paper indicate that participants were asked to act as if they would in real practice? | yes/no | | Does paper indicate that study took place in participants’ usual work setting? | yes/no | |
| Scenario format | Written/video-recording/photographic/other (please specify): | | | | |
| Method of delivery | Paper-based/computer-based/other (please specify): | | | | |
| Are example scenarios provided either in the paper or online? | Paper/online/neither | | | | |
| Does paper indicate that scenarios were piloted? | Yes/no | | | | |
| If yes, provide details | | | | | |
|  | | | | | |
| Does paper indicate that scenarios assessed for validity? | Yes/no | | | | |
| If yes, provide details | | | | | |
|  | | | | | |

Outcomes

| Decision required | Response format | Units of measurement and properties | Analysis methods |
| --- | --- | --- | --- |
|  |  |  |  |
|  |  |  |  |

| Is perceived decision difficulty assessed? | Yes/no |
| --- | --- |
| If yes: response format, units of measurement and properties, analysis methods | |
|  | |
| Is decision difficulty assessed in any other way? | Yes/no |
| If yes, how was this achieved and what analysis methods were used? | |
|  | |
| Is decision quality in terms of guideline adherence assessed? | Yes/no |
| If yes, how was this achieved and what analysis methods were used? | |
|  | |
| Is decision quality assessed in any other way? | Yes/no |
| If yes, how was this achieved and what analysis methods were used? | |
|  | |
| Is the relationship between decision difficulty and decision quality assessed? | Yes/no |
| If yes, how was this achieved and what analysis methods were used? | |
|  | |

Results

| Decision required | Number analysed | Findings |
| --- | --- | --- |
|  |  |  |
|  |  |  |

| Decision difficulty assessment |
| --- |
|  |

| Decision quality assessment |
| --- |
|  |

| Relationship between decision difficulty and decision quality |
| --- |
|  |

**Conclusions and additional information**

| Key conclusions of study authors (if related to decision difficulty, decision quality or scenario construction methods) | | | |
| --- | --- | --- | --- |
|  | | | |
| References to other relevant studies | | | |
| First Author | Year | Journal | Citation |
|  |  |  |  |
|  |  |  |  |

| Correspondence required? | yes/no |
| --- | --- |
| If yes: study contact details | |
|  | |

| Further comments if applicable |
| --- |
|  |
