# Supplementary material for: Perceived difficulty and appropriateness of decision making by General Practitioners: a systematic review of scenario studies
Source: BMC Health Serv Res. 2014 Nov 29;14:621. doi: 10.1186/s12913-014-0621-2 (PMC4258016; doi:10.1186/s12913-014-0621-2)
Supplement: Additional file 4: — Key Features of Included Studies. [file 12913_2014_621_MOESM4_ESM.doc]

**Additional File 4: Key Features of Included Studies**

Thirty-eight studies which assessed decision appropriateness and for which the number of appropriate & non-appropriate decisions could be extracted/calculated

| **First author, year, country** | **Study design & aims(s)** | **GP participants** | **Scenarios^a,b^** | **Decision types & findings^c^** | **Decision quality assessment method & results** |
| --- | --- | --- | --- | --- | --- |
| Abdulmajeed, 2000, United Arab Emirates [1] | Questionnaire survey within prospective pre-post intervention study  Evaluate 2 week psychiatry course | 14 recruited & analysed  43% M, 57% F  Median age 41, range 32-54 | 3  Depression/ alcohol dependency/ anxiety disorder with panic attacks | Diagnosis & treatment/management (subgroup NS)  Response formats NS  No basic decisional data | Expert panel  Scoring system created by 2 psychiatry & family medicine consultants: 60% of model score was cut-off for appropriate decisions  Pre-intervention:  No. appropriate decisions (42 possible for each):  Diagnosis: 9  Treatment/management: 10 |
| Ang, 2007, USA [2] | Questionnaire survey  Assess abilities in maximizing nonsurgical osteoarthritis treatment | 149 recruited & analysed  94 M, 55 F  31 <= 5 years’ experience; 30 6-10; 88 >= 11 | 10  Incl. age, gender, ethnicity, other conditions, medication, symptoms, current & previous management | Treatment/management (subgroup NS)  Multiple choice  Across scenarios, 4-95% chose nonsurgical & 5-96% chose surgical treatment | Literature  Used published total joint arthroplasty appropriateness criteria to determine appropriate decisions (consider surgery/maximize nonsurgical treatment)  No. appropriate treatment/management decisions: 1164 (1490 possible) |
| Arber, 2004 & 2006, Bonte, 2008, Lutfey, 2009a & 2009b, McKinlay, 2006 & 2007, Von dem Knesebeck, 2008, USA,UK & Germany [3-10] | Factorial experiment within interview survey  Examine influences on CHD & depression decisions, differences between countries, & guideline adherence | 384 recruited & analysed  (128 each country)  192 M, 192 F  50% <5 years’ experience, 50% >15 years | 32; 16 each diagnosis, each received 1 of each (in Germany saw CHD only)  Varied gender, age, SES, race (race USA & UK only)  Signs & symptoms of CHD/depression  Video scenarios | Screening/testing, diagnosis, & treatment/ management (test ordering, prescribing, giving advice, referral)  Open response  CHD scenario (UK & USA & Germany):  % diagnosing CHD: 88% & 95% & 74%  Mean 3.5 & 3.2 & 0.9 tests ordered  Mean 2.9 & 2.8 & 1.9 pieces advice given  31% & 10% & 19% referred to cardiologist/coronary specialist  Mean 13.1 & 11.3 & 5.7 days wait before re-consultation  Depression scenario (UK & USA):  % diagnosing depression: 90% & 93%  Mean 4.6 & 3.6 tests ordered  Mean 0.3 & 0.7 pieces advice given  4% & 16% referred appropriately  Mean 10.1 & 15.1 days wait before re-consultation | CHD: guidelines  No. appropriate decisions (384 possible for each, except test ordering):  Diagnosis: 330  Prescription: 169  Referral: 77  Giving advice: 122  Test ordering: 374 (768 possible)  Depression: DSM-IV used to create scenarios, but quality assessment standard unclear  No. appropriate decisions (256 possible for each):  Diagnosis: 234  Prescription: 63  Referral: 25 |
| Binard, 2009, France [11] | Questionnaire survey  Determine equivalence between polymyalgia rheumatica disease activity score calculated by different specialties | 163 recruited & analysed  No sex/age/ experience data | 7  Polymyalgia rheumatica patients spanning full spectrum of disease activity level | Diagnosis  Response format NS  Across scenarios, 5-95% gave relapse diagnosis  Also decided whether prednisone dosage increase needed: across scenarios, 5-93% increased prednisone dosage | Expert panel  35 rheumatologists, appropriate decision (relapse diagnosis/no relapse) that for which at least 80% agreed  No. appropriate diagnosis decisions: 1029 (1141 possible) |
| Chin, 1997, USA [12] | Questionnaire survey  Identify whether ACE inhibitors underused & if  physicians characteristics associated with patterns of self-reported use | 460 analysed  Family practitioners & Internists:  83% & 77% M  7% & 7% aged <35; 38% & 41% 35-44; 31% & 29% 45-54; 13% & 17% 55-64; 11% & 6% >=65 | 4  Patients with reduced ejection fraction  Asymptomatic/ chronic heart failure/ asymptomatic post-myocardial infarction/ symptomatic heart failure & ejection fraction=0.35 | Treatment/ management (prescribing)  Multiple choice  Rates of ACE inhibitors use (family practitioners & Internists):  Scenario 1: 68% & 78%  2: >88% & >88%  3: 58% & 70%  4: 72% & 76% | Guidelines  Prescribing ACE inhibitors was appropriate decision for all scenarios according to the guidelines used  No. appropriate prescribing decisions: 976 (1380 possible)  (Unable to calculate for scenario 2) |
| de Klippel, 2008, Germany, Belgium & Portugal [13] | Questionnaire survey  Assess accuracy in diagnosing & treating headache/migraine | 705 recruited & analysed  72% in community practice >15 years, 14% 10–15 years, 9% 5–10 years, 5% <5 years | 4  New migraine case/worsening migraine/chronic migraine/tension-type headache  Included signs, symptoms & medical history | One decision covered multiple categories: screening/ testing, diagnosis & treatment/ management (test ordering, examination, prescribing, giving advice, referral)  Multiple choice  Scenario 1: 66% chose ‘follow steps to diagnose migraine, prescribe triptan if confirmed’  2: 42% ‘recommend early intervention with a triptan’  3: 32% ‘counsel slow discontinuation of NSAIDs & prescribe antiepileptic drug to be slowly up-titrated’  4: 56% ‘complete formal evaluation of stress & anxiety levels’ | Guidelines  Used guidelines to determine appropriate decisions  No appropriate screening/testing, diagnosis & treatment/management decisions: 2047 (2820 possible) |
| Di Caccavo, 2000, UK [14] | Questionnaire survey  Investigate influence of race on diagnosis & treatment of psychological complaints | 18 recruited & analysed  10 M, 8 F | 18 (6 psychosis, 6 depression, 6 anxiety); each received 9  Varied gender & race  All presenting for first time, symptoms occurring over last few months  Written & photographic scenarios | Diagnosis  Open response  Also decided likelihood with which treatment/ management actions would be undertaken (options rated using 6-point Likert scales, never-definitely)  No basic decisional data | Guidelines  Used DSM-IV to create scenarios; psychosis/ depression/anxiety diagnoses were appropriate decisions  No. appropriate diagnosis decisions: 85 (162 possible) |
| Emery, 2000, & Glasspool, 2001, UK [15, 16] | Crossover experiment with balanced block design  Compare two types computer support with pen & paper methods for recording & interpreting family histories of cancer | 36 recruited & analysed  69% M  Median 21 years’ experience (range 7­36) | 18  Women with different HBOC risks  Each responded to 6 scenarios for each of the 3 support methods | Treatment/management (referral)  Response format NS  No basic decisional data | Combination (expert panel & current research)  GP & health services researcher used a published strategy to determine appropriate decisions (low risk, no referral; medium, refer to breast unit; high, refer to genetics clinic)  Control group: 36 GPs, 6 scenarios: No. appropriate referral decisions: 140 (216 possible) |
| Evink, 2000, USA [17] | Questionnaire survey  Examine extent of variation in process for diagnosing childhood ADHD & investigate whether different specialties differ in diagnosis & treatment | 36 analysed  No sex/age/ experience data | 3  Girl not meeting criteria for ADHD/boy, significant family dysfunction, prior diagnosis general learning disability/boy, classic ADHD | Diagnosis  Response format NS  Primary diagnoses:  1: normal behavioural for 41%; ADHD for 12%  2: 53% learning disability; ADHD 31%  3: ADHD 97%  Also decided which tests to use for further evaluation, whether to refer for further evaluation, & medications to first prescribe (if any)  Overall: 69% would refer, 15% would not prescribe initially | Guidelines  DSM-IV used to create one scenario which represented classic ADHD; appropriate decision was ADHD diagnosis  No. appropriate diagnostic decisions for that scenario: 35 (36 possible) |
| Ferreira, 2010, Portugal [18] | Questionnaire survey  Identify main differences in treatment of allergic rhinitis relative to guidelines | 402 recruited, 397 analysed  No sex/age/ experience data | 7  First 3 moderate -severe rhinitis (one intermittent, two persistent), 2 persistent moderate -severe rhinitis, 2 mild rhinitis (one intermittent, one persistent)  Included age, gender, occupation, symptoms, current medications, exam results | Treatment/management (prescribing)  Multiple choice  1: 51% prescribed non -sedating oral antihistamines  2: 53% start high doses topical nasal steroids & daily antihistamines, if necessary in double doses, discontinue topical vasoconstrictor  3: 45% non -sedating oral antihistamines  4&5&7: 78% & 67% & 64% oral non -sedating antihistamine, standard dose  6: 51% topical nasal steroids | Guidelines  Used guidelines to determine appropriate decisions & those representing under-/over-treatment  No. appropriate prescribing decisions: 800 (1586 possible)  (Decision quality calculated for scenarios 4-7 only) |
| Frayne, 2004a, & 2004b, USA [19, 20] | Interview survey  Compare depression decision making between veteran’s affairs & non-veterans affairs physicians & determine influence of patient gender &/or age | 243 recruited & analysed  58% M  Mean age 46 | 16, each received 1  Varied age, gender, race, & SES  Signs & symptoms of depression  Video scenarios | Diagnosis & treatment/ management (prescribing, referral, follow-up)  Open response  115 veteran’s affairs & 128 non-veteran’s affairs:  54% & 73% had depression likelihood >=50%  13% & 2% chose mental health referral  11% & 13% chose antidepressants  32%; 53% chose appropriate follow-up within 2 weeks | Guidelines  Used guidelines to determine appropriate decisions  No. appropriate decisions (243 possible for each):  Diagnosis: 155  Prescription & referral: 46 (antidepressant/mental health practitioner referral/ both)  Follow-up: 105 |
| Freund, 2003 & McKinlay, 2002, USA [21, 22] | Factorial experiment within interview survey  Investigate influences on decision making for late life depression/ polymyalgia rheumatica | 128 recruited & analysed  48 F  Mean 18 years’ experience | 32; 16 each diagnosis, each received 1 of each  Varied age, gender, race, SES  Signs & symptoms of depression/ polymyalgia rheumatica  Video scenarios | Diagnosis  Open response  Polymyalgia rheumatica scenario:  52 included polymyalgia rheumatica in differential diagnosis  Depression scenario:  121 included depression in differential diagnosis  Also decided lab tests to order, medication, referrals, & when to see patient for a return visit: 91 ordered thyroid function test; 16 chose antidepressants; 3 chose mental health referral; mean 21 days until follow-up visit | Depression: guidelines  DSM-III-R used to create scenarios, appropriate decision was depression diagnosis  No. appropriate diagnosis decisions: 121 (128 possible)  Polymyalgia rheumatica: quality assessment standard unclear  No. appropriate diagnosis decisions: 52 (128 possible) |
| Glazier, 1996 & 1998, Canada [23, 24] | Questionnaire survey  Examine influences on management of common musculoskeletal disorders & compare to expert panel recommendations | 529 recruited & analysed  354 M  Mean 14 years’ experience | 5  Shoulder problem/ moderately severe knee osteoarthritis/ acutely hot, swollen knee/early/late rheumatoid arthritis | Screening/ testing & treatment/ management (test ordering, prescribing, giving advice, referral)  Multiple choice  Scenario 1: 65% recommended ice/heat  2: 61% NSAID/ high-dose aspirin  3: 62% NSAID/ high-dose aspirin  4: 86% NSAID/ high-dose aspirin  5: 72% NSAID/ high-dose aspirin | Expert panel  Panel of 36 GPs, rheumatologists, physiatrists, orthopaedic surgeons, physiotherapists, occupational therapists & social workers determined appropriate decisions  No. appropriate decisions:  Test-ordering: 8498 (10051 possible)  Prescribing: 1159 (1587 possible)  Giving advice: 1795 (4232 possible)  Referral: 2614 (4761 possible) |
| Hillson, 1995, USA [25] | Interview survey within RCT  Examine influence of computer assisted test interpretation for electrocardiograms on decisions | 40 recruited & analysed  31 M  Mean 11 years’ experience | 10  Described history, physical examination, relevant lab findings, electro-cardiogram including original computer-generated report | Diagnosis  Response format NS  Participants reported 0-9 alternative diagnoses (in addition to leading diagnoses), mean 2.2 alternative diagnoses per scenario | Actual diagnosis  Two internists rated agreement between provided diagnoses & final diagnosis from actual consultation  Control group: 19 GPs, no. appropriate diagnosis decisions: 29 (190 possible) |
| Jiwa, 2008, Australia & UK [26] | Questionnaire survey  Investigate influences on referral decisions for colorectal symptoms (some with high cancer risk) | 260 recruited & analysed  133 M  48 had <5 years’ experience, 46 5-10, 48 10-15, 41 15-20, 76 20+ | 64; each received 9  Varied age, symptom duration, presence/ absence of: rectal bleeding, change in bowel habit, weight loss, iron deficiency anaemia | Treatment/ management (referral)  Multiple choice  1632/2367 scenarios selected for referral; 1012/2367 scenarios referred urgently  350/1433 cancer scenarios recognised as likely/very likely to have cancer | Guidelines  Used guidelines to determine appropriate decisions (referring cases with cancer symptoms urgently; routine referral for all other cases)  No. appropriate referral decisions: 1330 (2367 possible) |
| Jones, 1996, UK [27] | Questionnaire survey  Examine use of oral steroids & antibiotics & timing of follow-up in asthma attacks associated with URTIs | 185 recruited & analysed  No sex/age/ experience data | 2  45-year-old /9-year-old with acute asthma associated with URTI | Treatment/ management (referral)  Yes/no choice  Adult scenario: 6 referred  Child scenario: 14 referred  If not referring, decided which treatment to give & when to suggest review:  Adult scenario: 167/179 chose oral steroids, 90/179 review in 4-25 hrs  Child scenario: 148/169 chose oral steroids, 88/169 review in 4-25 hrs | Guidelines  In line with guidelines, management in primary care appropriate; referral to hospital not appropriate  No. appropriate referral decisions: 350 (370 possible) |
| Kales, 2005, USA [28] | Questionnaire survey  Investigate role of physician bias in racial disparities in diagnosis & treatment of late-life depression | 178 recruited & analysed  125 M | 4; each received 1  Varied gender & race  Depression signs & symptoms  Video scenarios | Diagnosis  Multiple choice  44 saw white F & 46 white M & 46 African-American F & 42 African-American M:  35 & 37 & 40 & 39 diagnosed depression  Also decided initial treatment, how long to follow patient, & referral:  36 & 41 & 41 & 38 chose antidepressant  15 & 16 & 19 & 12 follow-up months 1–3; 3 & 6 & 2 & 2 months 4–6; 22 & 13 & 17 & 19 months >6  3 & 6 & 4 & 7 chose non-medical doctor mental health provider | Guidelines  DSM-IV used to create scenarios representing depression, which was appropriate diagnosis  No. appropriate diagnosis decisions: 151 (178 possible) |
| Kuyvenhoven, 1984, The Netherlands [29] | Simulated decision making experiment  Assess content validity of scenarios by establishing whether acute appendicitis identified & treated accordingly | 19 recruited & analysed  All established in practice for at least 5 years | 6  5 patients with common, non-acute complaints, 1 patient with acute, severe signs & symptoms clearly suggesting acute appendicitis | Diagnosis  Open response  Also decided what to tell the patient (if considered necessary, could refer/prescribe) :  All referred acute appendicitis patient to a surgeon  Patients with vague complaints: differences in therapeutic procedure included differences in medication, referral & appointments for a second encounter | Actual diagnosis  Real patient data used to create one scenario clearly representing acute appendicitis, which was appropriate diagnosis  No. appropriate diagnostic decisions: 19 (19 possible) |
| Murray, 2000, Australia [30] | Pilot questionnaire survey  Determine influence of signs & symptoms on likelihood of prescribing antibiotics for URTI | 20 recruited & analysed  No sex/age/ experience data | 32  20-year-old with URTI, no patient pressure to prescribe  Signs & symptoms varied: runny nose, sore throat, fever, cough | Treatment/management (prescribing)  Response format NS  Total 640 decisions: antibiotics prescribed for 212/640 | Literature  Scenarios constructed to represent cases for which prescribing antibiotics inappropriate based on best available evidence in literature; not prescribing therefore appropriate decision  No. appropriate prescribing decisions: 428 (640 possible) |
| Park, 2006, USA [31] | Questionnaire survey  Evaluate management of children with acute pharyngitis | 590 recruited, 525 analysed  368 M  Median 14 years’ experience (range 1–42) | 2  6-year-old with clinical & epidemiological findings consistent with bacterial pharyngitis/with findings consistent with viral pharyngitis | Screening/ testing & treatment/ management (test-ordering & prescribing)  Multiple choice  1 (N=514): 30% chose rapid strep test, if pos, give antibiotics; if neg, throat culture, give antibiotics while awaiting results, stop antibiotics if neg  2 (N=517): 39% chose rapid strep test, if pos, give antibiotics, if neg, nothing further | Guidelines  In line with guidelines, test-ordering & prescription (if discontinued for negative test result) were appropriate decisions for scenario 1; not ordering tests or prescribing were appropriate decisions for scenario 2  No. appropriate test ordering & prescribing decisions: 371 (1031 possible) |
| Persell, 2010, USA [32] | Questionnaire survey  Examine influence of 10-year risk estimates & lifetime risk on preventive cardiology decisions | 104 recruited, 99 analysed  58 M, 41 F  29 had <10 years’ experience; 38 11-20; 25 >20 | 5  First presented CHD risk factor information alone, then with estimated 10-year risk estimates, then with 10-year & lifetime risk estimates | Screening/testing & treatment/management (test-ordering & giving advice (multiple choice), prescribing (yes/no choice)  % not prescribing ranged from 7% to 74% across scenarios  For test-ordering & giving advice, decided when to repeat lipid testing: a) 5 years; b) 1 year; c) 6 months; d) 6 weeks & initiate therapeutic lifestyle change; e) start therapy without retesting  Scenario 1: 82% chose a-c  2: 46% chose a-c  3: 86% chose b-d  4: 78% chose b-d  5: 81% chose b-d | Guidelines  appropriate decisions according to guidelines:  Prescribing: scenario 2: prescribing aspirin; not prescribing for all other scenarios  Test-ordering & giving advice: scenarios 1 & 2: giving lifestyle advice & testing in 6 weeks (or, for scenario 2, prescribing & giving advice without testing); scenarios 3-5: testing in 5 years  No. appropriate decisions (495 possible for each):  Prescribing: 327  Test-ordering & giving advice: 84 |
| Rose, 2001, UK [33] | Postal questionnaire survey  Assess referral for women with different HBOC risk | 164 recruited & analysed  109 M, 55 F  18 had 0-10 years’ experience, 67 11-20, 57 21-30, 22 31-40 | 6  Women with different HBOC risk | Treatment/management (referral)  Response format NS  No basic decisional data | Literature  Appropriate decisions according to literature: low risk cases managed at primary care (not referred); moderate/high risk cases referred  No. appropriate referral decisions: 514 (933 possible) |
| Schulman, 1999, USA [34] | Questionnaire survey  Assess influence of patient race & gender on treatment for patients with various types of chest pain | 720 recruited & analysed  494 M, 226 F | 144; each received 1  Varied race, gender, age, coronary risk, chest pain type, results of exercise stress test with thallium  Video scenarios | Diagnosis  Response format NS  31% cases classified as definite angina, 65% possible angina, 4% non-anginal chest pain  Also decided whether to order further cardiac evaluations: stress tests recommended for 93% white M & white F & 98% black M & black F  Also decided whether to refer for cardiac catheterization: mean referral rates: 84% nonanginal pain cases; 90% possible angina; 89% definite angina | Combination  Guidelines & expert panel: four cardiologists, used guidelines & diagnosed scenarios as representing definite angina/possible angina/nonanginal chest pain (appropriate diagnoses)  No. appropriate diagnosis decisions: 367 (720 possible) |
| Shackelton, 2009a & 2009b, USA [35, 36] | Factorial experiment within interview survey  Examine influences on diabetes decision-making & determine whether clinical guidelines achieve intended consequences | 192 recruited, 122 analysed  No sex/age/ experience data | 24; each received 1  Varied age, race, gender & SES  Patient with diabetes & symptoms suggestive of peripheral neuropathy  Video scenarios | Screening/ testing (examination)  Response format NS  57 would perform visual examination for ulcers, 77 would perform vibration/ monofilament exam, 82 would check foot pulses, 52 would do all three  Also asked about prescriptions, lifestyle recommendations, referrals | Combination  Guidelines & one expert: according to guidelines & a consulting diabetologist: visual inspection for ulcers, vibration/monofilament exam, & palpation of foot pulses examinations should all be performed (appropriate decision)  No. appropriate examination decisions: 52 (122 possible) |
| Shackelton-Piccolo, 2011, USA [37] | Factorial experiment within interview survey  Determine whether there are differences in decisions for a patient with CHD between different specialties | 384 recruited, 367 analysed  182 M, 185 F  180 had more experience, 187 less | 16; each received 1  Varied age, gender, race, SES  CHD signs & symptoms  Video scenarios | Screening/ testing (test ordering), diagnosis, treatment/management (prescribing)  Open response  97% included CHD & gastrointestinal illness in differential diagnosis  192 family practitioners & 175 internists:  47% & 68% ordered stress test  17%; 29% prescribed beta blockers  35%; 45% prescribed nitrate  40% overall prescribed aspirin  Mean 4.2; 3.1 pieces of advice given | Combination  Guidelines, expert panel & literature: according to guidelines, literature & advice from clinical consultants, ordering stress test & electrocardiogram & prescribing beta blockers & nitrate & aspirin were all appropriate decisions; appropriate diagnosis was inclusion of CHD in differential diagnosis  No. appropriate decisions:  Diagnosis: 356 (367 possible)  Test ordering: 209 (367 possible)  (Unable to calculate for ordering of electrocardiogram)  Prescribing: 377 (1101 possible) |
| Sohn, 2007, USA [38] | Questionnaire survey  Investigate recommendations for supplemental fluoride use based on caries risk status of infants & toddlers | 383 recruited & analysed  74% M  All had about 20 years’ experience | 2  Described oral condition & general health status of 12 month old children  Healthy girl at low risk for dental caries/boy at high risk for dental caries  Written & photographic scenarios | Treatment/ management (prescribing, giving advice)  Response formats NS  For scenarios 1 & 2:  77% & 76% recommended fluoride supplements  59% & 70% recommended brushing with fluoridated toothpaste | Expert panel  Centers for Disease Control & Prevention experts determined appropriate decisions: prescribing fluoride supplements for scenario 2 & not for scenario 1; advise brushing with small amount fluoridated toothpaste for both scenarios  No. appropriate decisions (766 possible for each):  Prescribing: 348  Giving advice: 494 |
| Spiegel, 2010, USA [39] | Questionnaire survey  Investigate diagnostic decision-making in irritable bowel syndrome | 89 recruited & analysed  57% M  Mean 26 years’ experience | 2  Patients with irritable bowel syndrome symptoms | Diagnosis  Yes/no choice  Scenario 1: 34% chose yes, 7% no, 59% unsure  Scenario 2: 17% chose yes,19% no, 64% unsure | Guidelines  Scenarios designed to represent Rome III criteria for irritable bowel syndrome (appropriate diagnosis)  No. appropriate diagnosis decisions: 45 (178 possible) |
| Stoppe, 1994, 1995a, 1995b, 1996, Germany [40-43] | Interview survey  determine differences in treatment of Alzheimer's disease & vascular dementia & investigate diagnostic accuracy | 145 recruited & analysed  No sex/age/ experience data | 4; each received 2  1: Slight, common non-specific memory problem: patient either did/didn’t demand drugs  2: Vascular dementia/ Alzheimer's disease | Diagnosis  Open response  Scenario 1: 56% had vascular encephalopathy in differential diagnosis  2: no GP-only data  For scenario 2, also asked how to treat sleep disturbances: 42% chose neuroleptics  Prescribing: no GP-only data | Guidelines  according to DSM-III-R & International Classification of Diseases 10 criteria, depression should be main differential diagnosis for scenario 1  No. appropriate diagnosis decisions: 59 (145 possible)  (Unable to calculate for scenario 2) |
| Stoppe, 2007, Germany [44] | Interview survey  Measure diagnostic competence for moderate dementia | 122 recruited & analysed  56% M | 4; each received 2; almost identical to those in previous survey; only 1 analysed:  Vascular dementia/ Alzheimer’s disease | Diagnosis  Open response  61 per case: 61 & 54 had vascular dementia in differential diagnosis  Also asked about screening/testing & referral: 69% & 69% took history; 82% & 67% did blood analysis; 79% & 84% would refer | Guidelines  Scenarios described vascular dementia (scenario 1)/ Alzheimer’s disease (scenario 2) according to International Classification of Diseases 10: inclusion in differential diagnoses are appropriate decisions  No. appropriate diagnostic decisions: 100 (122 possible) |
| Tiemeier, 2002, The Netherlands [45] | Questionnaire survey  Determine variation in treatment decisions & adherence to guidelines for depression treatment | 57 recruited & analysed  No sex/age/ experience data | 22  Described an episode of major or subthreshold depression | Treatment management (prescribing, monitoring, treatment other than prescribing)  Multiple choice  N=1246 decisions: 157 decisions for watchful waiting, 298 psychotherapeutic management, 136 psychotherapy, 417 psycho-pharmacotherapy, 238 combined therapy | Combination  Guidelines & expert panel used to determine appropriate decisions & under-/over-treatment  No. appropriate prescribing & monitoring & treatment other than prescribing decisions: 827 (1246 possible) |
| Tucker, 2003, UK [46] | Questionnaire survey  assess diagnosis & management of mild nonproteinuric hypertension against guideline recommendations | 117 recruited & analysed  No sex/age/ experience data | 2  Slightly raised diastolic blood pressure & estimation of protein in the urine by dipstick of only “+” proteinuria/ elevated blood pressure, negative urinalysis | Screening/ testing, diagnosis & treatment/management (test ordering, investigation, examination, monitoring)  Response formats NS  Scenario 1: 109 provided a diagnosis; 67 pre-eclampsia  107 provided management; 63 did not recommended admission, 88 did not recommend referral for specialist opinion/management | Guidelines  According to guidelines, appropriate diagnosis for scenario 1 mild non-proteinuric hypertension/ hypertension/pregnancy induced hypertension; appropriate management for both cases: blood pressure recording & urine dipstix twice weekly, clinical appraisal of maternal & foetal well-being & foetal size, & single blood tests  No. appropriate decisions:  Diagnosis: 12 (109 possible)  Test ordering & investigation & examination & monitoring: 8 (215 possible) |
| Vancheri, 2008 & 2009, Sweden & Italy [47, 48] | Questionnaire survey  Investigate whether physicians in countries with different cardiovascular risk levels make different lipid-lowering treatment decisions | 76 recruited & analysed  38 Stockholm: 40% M  Median age 54, range 43–65  38 Sicily: 76% M  Median age 51, range 42–70 | 9  Included age, gender, systolic blood pressure, cholesterol, smoking  Spectrum of high-low 10-year risk of coronary event | Treatment/ management (prescribing)  Yes/no choice  Means of proportions of decisions to start treatment (no. yes decisions/ total no. decisions for each GP):  Stockholm:  All cases: 0.46  High-risk cases: 0.82  Low-risk cases: 0.18  Sicily:  All cases: 0.54  High-risk cases: 0.88  Low-risk cases: 0.27 | Literature  According to research, prescribing lipid-lowering treatment appropriate for cases with 10-year absolute CHD risk >= 20% & not appropriate for lower risks  No. appropriate prescribing decisions: 547 (670 possible) |
| Watson, 2001, UK [49] | Questionnaire survey within cluster RCT  Evaluate effectiveness of two educational interventions for referral decision making | 426 recruited & analysed  63% M  Mean 18 years’ experience | 6  Women with range of HBOC risk levels (low/med/high) | Treatment/ management (referral)  Yes/no choice  No basic decisional data | Not specified  Calculated number making appropriate referral decisions, but unclear which quality assessment standard used & how used  Control group 162 GPs, no. appropriate referral decisions: 675 (972 possible) |
| Webster, 2005 & 2006, USA [50, 51] | Questionnaire survey  Assess extent to which decisions for back pain consistent with guidelines & whether responses varied with presentation of sciatica or by physician characteristics | 419 recruited & analysed  No sex/age/ experience data | 2  Nonspecific back pain/sciatica with neurologic findings | Treatment/ management (prescribing, giving advice, treatment other than prescribing)  Response format NS  Also asked about test-ordering & referral  Speciality: 103 general practice & 196 family practice & 120 internal medicine:  Scenario 1: 45% & 16% & 26% chose x-ray  82% & 90% & 91% gave education on low back pain  84% & 94% & 90% prescribed NSAIDs  27% & 9% & 18% referred to specialist  Scenario 2: 70% & 65% & 71% chose Magnetic Resonance Imaging  80% & 86% & 90% gave education on low back pain  75% & 91% & 81% prescribed NSAIDs  95% & 75% & 87% referred to specialist | Guidelines  Used guidelines to determine appropriate decisions  No. appropriate decisions:  Prescribing: 2287 (3352 possible)  Giving advice: 1414 (2095 possible)  Treatment other than prescribing: 31 (419 possible) |
| White, 2008, USA [52] | Factorial experiment within questionnaire survey  Examine whether patient request for genetic test influences referral decisions | 284 recruited & analysed  71% M  Half were > 15 years out of residency | 8; each received 1  Female patient presenting for annual exam & requesting genetic testing for breast cancer  Varied level of worry, insurance status, race  Written & photographic scenarios | Treatment/ management (referral)  Multiple choice  8% chose neither genetic testing nor genetic counselling, 23% chose both, 13% genetic testing, 50% genetic counselling, 6% other | Guidelines  Scenarios designed such that according to guidelines, referral to genetic counselling & testing not appropriate  No. appropriate referral decisions: 23 (284 possible) |
| Wijeratne, 2009, Australia [53] | Questionnaire survey  Determine recognition of common mental disorders of late life, & examine investigation, specialist referral & initial treatment decisions | 436 recruited & analysed  No sex/age/ experience data | 4  Older male suffering from depression/ dementia/ delirium/CHD (control scenario) | Diagnosis  Open response  Rate of recognition of: depression 93%; dementia= 98%; CHD= 99%  Also asked about test ordering, investigations, referral, giving advice & prescribing:  Depression: 97% chose blood sugar, 52% refer to psychogeriatrician 83% advise walking, 68% prescribed antidepressant  Dementia: 99% electrocardiogram, 95% cardiologist, 80% eat more fruit & veg, 69% Beta blocker/ACE inhibitor  CHD: 96% full blood count, 64% geriatrician, 70% eat more fruit & veg, 54% cholinesterase inhibitor | Not specified  Provided % diagnosing depression/CHD/dementia (appropriate decisions), but unclear which quality standard used  No. appropriate diagnostic decisions: 1264 (1308 possible)  (Data for delirium scenario not provided) |
| Windak, 2008, 2009, 2010a & 2010b, Pol& [54-57] | Questionnaire survey  Assess influences on decision making for hypertensive patients with different levels of cardiovascular risk & how decisions differ from guideline recommendations | 125 recruited & analysed  64% F  Mean 14 years’ experience | 8  Varied blood pressure, presence/ absence other cardiovascular disease risk factors, presence/ absence diabetes | Diagnosis & treatment/ management (prescribing, giving advice)  Open response  Most common prescriptions: ACE inhibitors (47%) & diuretics (24%)  Also asked about test-ordering: mean 4.9 tests ordered per case; serum lipid profile most frequently ordered (658 times) | Guidelines  Used guidelines to determine appropriate decisions  No, appropriate decisions (1000 possible for each):  Diagnosis: 548  Prescribing: 519  Giving advice: 183  (Also examined test-ordering: total 4988 tests ordered, mean 4.9 tests per case: represents 48% tests which should be ordered according to the guidelines) |
| Yanovski, 1992, USA [58] | Interview survey  Examine telephone triage decisions | 21 recruited & analysed  No age/sex/ experience data | 3  11-month-old with severe diarrhoea & dehydration/18-month-old with minor head trauma/2½-year-old with fever | Treatment/ management (appointment scheduling)  Response format NS  No basic decisional data | Combination  Guidelines, expert panel & literature: according to guidelines, literature & panel of 2 GPs & 3 paediatricians, child with diarrhoea & dehydration should be examined immediately (appropriate decision)  No. appropriate appointment scheduling decisions: 11 (21 possible) |

**Notes:** *ACE=angiotensin converting enzyme; ADHD=attention deficit hyperactivity disorder; CHD= coronary heart disease; DSM=Diagnostic and Statistical Manual of Mental Disorders; F=female; GP= General Practitioner (used to represent any variant in terminology used e.g. primary care physician /family physician etc.); HBOC=hereditary breast & ovarian cancer; M=male; NS=not specified; NSAID=nonsteroidal anti-inflammatory drug; RCT=randomised controlled trial*; *SES=socioeconomic status; URTI=upper respiratory tract infection*

*^a^Unless otherwise specified, participants responded to all scenarios;* **^b^***written scenarios unless otherwise specified;* ^c^*where ‘no basic decisional data’ is stated, studies did not report any descriptive statistics in relation to decisions made in response to scenarios*

*Twenty-eight studies which assessed decision appropriateness and for which the number of appropriate & non-appropriate decisions could not be extracted/calculated*

| **First author, year, country** | **Study design & aims(s)** | **GP participants** | **Scenarios^a,b^** | **Decisions & findings^c^** | **Decision quality results** |
| --- | --- | --- | --- | --- | --- |
| Backlund, 2000, Sweden [59] | Questionnaire survey  Investigate factors influencing lipid-lowering drug prescribing for hyper-cholesterolemia, & determine concordance with guidelines | 38 recruited & analysed  26 M, 12 F  Mean age 48 | 40  Patients with at least mild elevation of blood cholesterol values | Willingness to prescribe a lipid-lowering drug (visual analogue scale; 0% prescription totally unreasonable - 100% prescription obvious)  Total mean scale rating 54%, lowest mean rating for a scenario 28%, highest 78% | Appropriate decisions, according to guidelines: prescribing pharmacological treatment for 12 scenarios describing CHD; consider pharmacologic treatment for 11 scenarios; non-pharmacological treatment/advice for 17 scenarios  Mean scale ratings:  12 CHD scenarios: 65%  11 consider pharmacologic treatment scenarios: 62%  17 non-pharmacologic/advice scenarios: 40% |
| Bonetti, 2005, UK [60] | Questionnaire survey within RCT  Investigate whether audit & feedback &/or educational reminders influence lumbar x-ray referral for back pain | 214 recruited, 152 analysed (follow-up)  100 M, 52 F  Mean 20 years’ experience, range 5-37 | 20, 10 baseline, 10 follow-up  Included 5–8 elements  Stratified by appropriateness & age | Whether to refer for lumbar x-ray  Yes decisions summed per GP  Mean score: 3.64 baseline, 3.16 follow-up  No. decisions to refer range 0-9 baseline, 0-10 follow-up | Scenarios included data relevant to referral decision according to guidelines; expert opinion determined criteria for appropriate referral decisions  For 2/10 scenarios in each set, referral was appropriate decision: no. referring only these cases:  Baseline: 1  Follow-up: 6 |
| Carroll, 2011, Canada [61] | Questionnaire survey within RCT  Develop & evaluate knowledge translation intervention for genetics referrals | 125 recruited, 80 analysed  31 M, 49 F  Years’ experience range 2–40 | 10  Women with different HBOC risk | Whether to refer to a cancer genetics clinic  No basic decisional data | Used guidelines to determine appropriate decisions  Calculated quality score (max=10): scored 1 for every appropriate decision; scores then summed  Mean scores:  Baseline: control 7.1; intervention 6.5  Follow-up *(*adjusted for baseline scores): control 6.4; intervention 7.8 |
| Chavannes, 2004, The Netherlands [62] | Interview survey  Determine abilities in differentiating between chronic respiratory diseases when spirometry provided | 39 recruited, 36 analysed  33 M, 3 F  Mean age 48 | 12  Included medical history & physical exam results, absolute & predicted post bronchodilator spirometry test results | After spirometry:  Differential diagnosis: mean 1.35 diagnoses per case considered, max 6  Test-ordering: 28% probability of initiating diagnostic prednisolone course  Referral: 32% probability of referral  Prescribing: 39% probability of prescription | Panel of two pulmonologists, a pulmonary physiologist, & a GP reached consensus on spirometric diagnoses; appropriate decisions were those agreeing with panel consensus  % agreement with expert panel:  Obstruction: 91%  Normal spirometry: 78%  Incorrect manoeuvres: 65%  Rare pathological curves: 41% |
| Cherkin, 1994, USA [63] | Questionnaire survey  Determine what tests advised for low back pain, differences between specialties, & appropriateness | 169 recruited & analysed  No age/sex/ experience data | 3  Acute back pain alone/acute pain & sciatica/chronic low back pain | Imaging studies: overall, GPs equally likely to order magnetic resonance imaging/computed tomography without contrast; diskography almost never ordered | Used guidelines to determine appropriate decisions; ordering imaging tests for scenarios 1 & 3 inappropriate, questionable for scenario 2  % GPs ordering different tests presented in graphs so cannot extract |
| Christian, 2006, & Mosca, 2005 USA [64, 65] | Questionnaire survey  Examine influences on cardiovascular disease preventive care & awareness of & barriers to adoption of prevention guidelines | 300 recruited & analysed  81% M  Mean 16 years since completing residency | 16  Each received 10 (2 low risk, 4 intermediate & high risk)  2 low risk: only gender differed  4 intermediate risk: varied age, gender, race, cholesterol, blood pressure  4 high risk: varied age, gender, cholesterol, personal history CHD/diabetes | N=500 (incl. 100 obstetricians/ gynaecologists & 100 cardiologists):  Cardiovascular disease preventive treatment recommendations: most popular recommendation always physical activity | Used guidelines to evaluate decision quality  Appropriate decisions per scenario not analysed: scenario results amalgamated with results from other questions |
| de Melker, 1991, The Netherlands [66] | Questionnaire survey  Describe management of URTIs in light of standards & current research | 376 recruited & analysed  No age/sex/ experience data | 20  4 described URTIs: acute/recurrent tonsillitis/acute otitis media/ sinusitis | Decided how to treat & manage  Scenario 1: 52% chose antibiotics  2: 85% referral  3: 65% symptomatic medication  4: 84% antibiotics | Developed criteria based on guidelines & research findings which was used to evaluate decision quality  There were no explicitly appropriate/non-appropriate decisions: overall, found that type, dose & duration of antibiotics prescribed were in general agreement with guidelines but some aspects of prescribing practices were not |
| Elinson, 1999, Canada [67] | Questionnaire survey  Determine patterns of & reasons for prescribing HRT, use of investigations & surveillance of HRT, & consistency with existing recommendations | 249 recruited & analysed  No age/sex/ experience data | 3  Healthy 51-year-old, experiencing severe menopausal symptoms/ healthy 54-year-old, no menopausal symptoms/40-year-old, undergone hysterectomy & bilateral oophorectomy | N=327 (incl. 78 gynaecologists):  Scenario 1 (N=308): 97% prescribed combined formulation  Scenario 3 (N=316): 82% prescribed oestrogen alone | Used guidelines to determine appropriate decisions: combined hormone replacement therapy for scenario 1; only oestrogen for scenario 3  Incl. gynaecologists, % making appropriate decisions:  Scenario 1: 97%  Scenario 3: 82% |
| Everitt, 1990, & Mahoney, 1994, USA [68, 69] | Interview survey  Explore concordance between nurse practitioner & GP decisions | 501 recruited & analysed  24% <6 years’ experience, 35% 6-20, 41% >20 | 6; each received 3  New patient seeking help for insomnia/joint pain/stomach discomfort  1/3 received middle-aged, patients, others received older patients | Insomnia scenarios: 65% chose prescription; 39% counselling; 29% lifestyle change  Prescription selected as single most effective therapy for 46% of 373 assessing older patient  Counselling single most effective therapy for 53% of 128 assessing younger patient | Panel of 2 geriatricians, 2 gerontological nurse practitioners, & 2 geriatric pharmacists determined appropriate decisions: panel rated responses on 4-point scale, very inappropriate-very appropriate  Only responses rated as appropriate contributed to final score: 1 point awarded for every matching item  373 GPs completing 3 geriatric scenarios:  Appropriateness score mean 13.3, range: 1-31 |
| Ferris, 1993, USA [70] | Questionnaire survey  Review decision making for abnormal cervical cytology reports & assess influences on decision making | 115 recruited & analysed  93 M, 21 F, 1 no data  Mean 11.5 years’ experience, 59 < 10, 50 >10, 6 no data | 2  Inflammatory Pap smear, included colour photograph/ high-grade squamous intraepithelial lesion: included colour photograph, colposcopic examination of silicone model & microscopic examination | Scenario 1 (N=110): 71 chose to evaluate aetiology, treat & repeat smear at 2-6 months  Scenario 2 (N=104): 43 referred to specialist | Decision quality assessed in reference to epidemiologic & scientific data:  scenario 1: empiric treatment approach inappropriate: scenario 2: colposcopic examination appropriate  Unclear whether other options were appropriate/ inappropriate  Majority of responses appropriate  Scenario 1: Most organisms likely to cause inflammatory smear appropriately chosen for testing.; empiric treatment most common inappropriate decision  Scenario 2: Most selected colposcopic evaluation |
| Fortinsky, 1995, USA [71] | Questionnaire survey  Investigate extent of variation in diagnostic & management approach for dementia & influences on decisions | No. recruited & analysed NS  No age/sex/ experience data | 2; each received 1  72 -year-old with mild/moderate symptoms of cognitive dysfunction | N=498 (incl. osteopathic medicine practitioners)  Scenario 1 & 2:  Mean 8.7 & 9.5 tests ordered  85% & 82% selected for referral to local chapter Alzheimer’s association | Used literature to determine appropriate decisions; included 6 tests deemed most appropriate to rule out reversible dementia & one of selection of cognitive tests  % ordering appropriate tests (incl. osteopathic medicine practitioners):  Complete blood count: 92%; rapid plasma regain: 66%; thyroid stimulating hormone: 89%; thyroxine: 56%; B12: 76%; geriatric depression scale: 28%; cognitive test: 58% |
| Foster, 2007, USA [72] | Questionnaire survey  Compare COPD decisions to guideline recommendations | 943 recruited, 784 analysed  Of 462:  344 M, 118 F  Year of graduation: 1 <1949; 37 1950-1969; 282 1970-1989; 142 1990+ | 2  58-year-old current smoker, persistent dyspnea, normal chest radiograph, unresponsive to antibiotics & short-acting bronchodilator/ 61-year-old M, smoker, subtle respiratory symptoms as secondary problem | 91% chose spirometry for scenario 1, 66% for scenario 2  Treatment: scenario 1: 127 chose long-acting anticholinergic; scenario 2: 251 chose inhaled corticosteroid | Used guidelines to evaluate decisions quality; different guidelines mentioned throughout, decision appropriateness varies depending on guidelines used |
| Hobus, 1987, not specified [73] | Interview survey  Explore reasons for differences in diagnostic accuracy between experts & novices | 18 recruited & analysed  Mean 11.1 years’ experience | 32  Patients with complaints relating to different organ systems  Included picture of patient (to convey age & gender) | Most likely diagnosis  No basic decisional data | Diagnosis received by actual patient used to determine appropriate decisions  Mean 12.11 appropriate diagnostic hypotheses generated |
| Hummers-Pradier, 1999, The Netherlands & Norway & Germany & Sweden [74] | Questionnaire survey  Identify factors predicting non-optimal treatment decisions for UTI | 453 recruited & analysed  No age/sex/ experience data | Sweden: 26  Others: 18  Varied day of week, known/unknown patient, age, history of UTIs, pain & frequency, blood in urine | Prescribing  No basic decisional data | Used guidelines to determine appropriate decisions; prescribing 2^nd^ as opposed to 1^st^ choice antibiotics/ symptomatic drugs & prescribing long as opposed to short courses was inappropriate  N=91 Netherlands & 99 Norway & 59 Germany & 204 Sweden:  Mean proportion non–optimal decisions:  Prescribing 2nd choice antibiotics: 10% & 1% & 28% & 33%  Prescribing long courses:  80% & 61% & 53% & 7% |
| James, 1998, USA [75] | Questionnaire survey  Investigate influences on congestive heart failure treatment decisions & determine whether non-disease factors conflicting with guideline recommendations affect adherence | 459 recruited & analysed  80% M  Mean 18 years’ experience | 6; each received 2  3 scenario pairs, patient-centred conflict with guideline-recommended care/no conflict  Pair 1: Financial difficulty  Pair 2: Reduced quality of life due to co-morbidity  Pair 3: Wish to remain in community with family | Pair 1: drugs recommended by 78% in conflict situation (N=142),70% in no conflict situation (N=165)  Pair 2: referral to cardiologist by 86% in conflict situation (N=149), 84% in no conflict situation (N=142)  Pair 3: treadmill recommended by 52% in conflict situation (N=165), 42% in no conflict situation (N=149) | Participants rated likelihood of performing activities recommended in guidelines; not clear which specific actions were appropriate/ inappropriate for each scenario |
| Kostopoulou, 2008, UK [76] | Questionnaire survey  Investigate role of information gathering & clinical experience on diagnosis & management of difficult diagnostic problems | 63 recruited & analysed  21 had 1-3 years’ experience, 42 >=10, range 10–31, mean 17 | 7  Contained cues critical for diagnosis & other cues used in diagnosis & 1-4 features of difficulty  Fatigue/ headache/ dyspnea (x2)/chest pain/abdominal pain/pyrexial child | Diagnosis & management  No basic decisional data | Diagnosis: experts (GPs with specialist interest in relevant clinical area) estimated likelihood of each differential diagnosis, & top-ranking diagnosis taken as the appropriate diagnosis; participants diagnoses appropriate if included in final list of differentials  Management: appropriate decisions informed by clinical guidelines for the diagnosis  No. appropriate decisions for whole sample (incl. 21 residents) ranged from 21 to 48 across scenarios for diagnosis, & from 26 to 62 across scenarios for management |
| Marwill, 1996, USA [77] | Questionnaire survey  Describe breast cancer screening practices for older women & examine influences on screening recommendations | No. recruited & analysed NS  No age/sex/ experience data | 8; 2 versions of each scenario, each received 4  1. No illness or impairment, aged 81/65  2: Mild dementia, mild functional impairment, lived with daughter/in nursing home  3: Functionally limiting osteoarthritis, lived independently/ depended on friend  4: Multiple comorbidities, complex medication, active in community, chronic & stable illnesses (2 sets) | N=482 (incl. obstetrics & gynaecology & geriatrics specialists):  Clinical breast exam recommended for all scenarios | According to guidelines, women in all scenarios required screening  % preforming screening mammogram (incl. obstetrics & gynaecology & geriatrics specialists):  Scenario 1a: 98%; 1b: 92%  Scenario 2a: 86%; 2b: 70%  Scenario 3a: 96%; 3b: 95%  Scenario 4a: 95%; 4b: 91% |
| Middleton, 2003, Australia [78] | Questionnaire survey  Examine knowledge about stroke issues | 296 recruited & analysed  208 M, 88 F  Median age 47 years (range, 28-81) | 4  Previously symptomatic, recent non-disabling stroke/ symptomatic, recent carotid transient ischemic attack/ asymptomatic, neck bruit/ asymptomatic, previous carotid endarterectomy | Likelihood of conducting examination & investigation tasks & management tasks (highly likely-highly unlikely scales)  Scenario 1: 99% highly likely to measure blood pressure  2: 93% highly likely to listen for neck bruits  3: 84% highly likely to order carotid ultrasound  4: 75% highly likely to prescribe aspirin | Used published research to determine appropriate decisions  % highly likely to perform recommended actions:  Scenario 1: 99% measure blood pressure; 78% order carotid ultrasound; 85% manage all uncontrolled risk factors; 72% observe, monitor & consider aspirin  2: 78% order carotid ultrasound; 41% order computed tomography scan of brain; 87% manage all uncontrolled risk factors; 54% observe & consider aspirin  3: 85% order carotid ultrasound; 78% listen for cardiac murmur; 51% assess brachial pulse  4: 58% order carotid ultrasound; 75% prescribe aspirin |
| Peabody, 2004, Macedonia^d^ [79] | Questionnaire survey  Determine if scenarios useful for making explicit cross-national comparisons of quality of clinical practice | 200 recruited & analysed  No age/sex/ experience data | 8  Coronary artery disease/low back pain/COPD/ diabetes: simple & more complex case of each | History taking/physical exam/ test ordering/ diagnosis/ treatment  No basic decisional data | Used guidelines & panel including generalists & expert specialists to determine appropriate decisions for each scenario within 5 domains: history taking, physical examinations, test ordering, diagnosis, treatment  Quality scores generated for each scenario & expressed as % correct (no. appropriate actions within domain performed/total number appropriate actions)  Mean score overall: 48%  Within domains: history 61%; examination 45%; test ordering 55%; diagnosis 39%; treatment 27% |
| Persson, 2004, Sweden [80] | Questionnaire survey  Investigate influences on risk assessment & treatment decisions for uncontrolled hypertension & concordance with guideline recommendations | No recruited & analysed NS  No age/sex/ experience data | 54; each received 12  Represented World Health Organisation guidelines risk groups: low/medium/ high/very-high | N=110 (incl. GP trainees)  Numbers willing to initiate/give further antihypertensive treatment in the 4 risk groups:  Low: 17  Medium: 149  High: 222  Very high: 416 | According to guidelines, as risk increases, willingness to prescribe should increase: specific appropriate decisions for each scenario unclear  % willing to prescribe: Low risk scenarios: 16%; med risk scenarios: 47%; high risk scenarios: 69%; very high risk scenarios: 77% |
| Poels, 2008, The Netherlands [81] | Simulated cluster RCT  Assess influence of expert support for spirometry test interpretation on decision-making for chronic respiratory disease | 78 recruited & analysed  64%, 57% M | 10  COPD/asthma/ allergic asthma/ lung fibrosis/no respiratory disease/incorrect test manoeuvre/ exercise-induced asthma  Intervention group received spirometry expert interpretation | Baseline:  Intervention group (357 scenarios) & control group (417 scenarios):  Probability of ordering additional diagnostic tests: 70% & 75%  Probability of referral: 19% & 18%  Follow-up:  Intervention group (216 scenarios) & control group (252 scenarios):  76% & 69% made medication/non-medication changes | Consensus diagnoses of two chest physicians, a GP & a health scientist represented appropriate decisions: assessed agreement between participants & panel  Baseline:  Intervention group: 66% agreement with expert panel  Control group: 66% agreement with expert panel  (For all cases except incorrect test manoeuvre case) |
| Ribacke, 1995a & 1995b, Sweden [82, 83] | Questionnaire survey  Examine treatment & follow-up in hypertension care & compare with a reference standard & practice reported by specialists | 76 recruited & analysed  Mean 6.2 years as specialists | 6  Included gender, age, occupation, smoking status, relevant history, symptoms, blood pressure, current therapy, values such as cholesterol & triglycerides | Most common treatment:  Scenario 1 & 3 & 5: 41% & 29% & 42% chose beta blockers  2: 42% diuretics  4: 39% ACE inhibitors  6: 34% calcium antagonists  Mean time to nurse follow up: within 4 weeks for 4 scenarios, 6 weeks for others | Responses of 7 GPs & 7 hospital specialists used to develop scoring system  Decisions made by >=5 experts (obligatory)=5 points  Decisions made by <=2 experts (superfluous)=1 point  All others (intermediate)= 3 points  Across 6 scenarios, 53 tests obligatory, 18 intermediate, 12 superfluous  Mean (range) scores per scenario:  1: 46.5 (25-62)  2: 37.3 (12-56)  3: 32.8 (8-46)  4: 33.5 (5-53)  5: 30.4 (10-50)  6: 43.4 (10-59) |
| Short, 2003, UK [84] | Questionnaire survey within before & after study  Investigate whether a decision support tool could assist with aspirin prescribing for secondary prevention of stroke | 15 recruited & analysed  13 M, 2 F  Mean 16 years’ experience, range 6–34 | 10  Stroke patients with complicating co-morbidity | Decided likelihood of prescribing aspirin (15 point scale, 1=no aspirin-unsure-15=aspirin; adapted from scale developed by Ottawa Hospital Research Institute)  Baseline & follow-up mean (range) scores per scenario:  1: 14.4 (10-15) & 14.3 (11-15)  2: 13.9 (8-15) & 14.8 (13-15)  3: 10.9 (1-15) & 13.2 (3-15)  4: 13.3 (9-15) & 14.6 (12-15)  5: 12.3 (8-15) & 13.7 (8-15)  6: 13.8 (9-15) & 14.3 (11-15)  7: 12.6 (8-15) & 14.3 (12-15)  8: 1.5 (1-4) & 1.9 (1-6)  9: 14.7 (13-15) & 14.5 (12-15)  10: 7.9 (1-15) & 7.8 (1-15) | According to guidelines, prescribing aspirin appropriate for 9 scenarios, not for 1 scenario (scenario 8, included contra-indication)  Baseline: mean scores ranged from 7.9 to 14.7 when prescribing appropriate |
| Smith, 2003, UK [85] | Questionnaire survey  Investigate influences on treatment of depression & compare with guidelines | 40 recruited & analysed  Roughly 2-to-1 ratio M-F  Years’ experience range 8-34, mean 19 | 20  Varied 5 symptoms of depression, duration of symptoms, patient preference, gender | Likelihood of prescribing antidepressant medication (0% definitely would not -100% definitely would scale) ranged from 0–100% in 13/20 scenarios | Used guidelines to determine which scenarios prescribing antidepressants was appropriate for  Took >=50% likelihood estimate to indicate that participants would prescribe  Mean 7.02/20 cases prescribed for: prescribing appropriate for 5 cases: overprescribed by 40% |
| Wagner, 1996, Canada & USA [86] | Questionnaire survey  Evaluate whether eosinophilia-myalgia syndrome more likely to be diagnosed when clinical history includes l-tryptophan use | No. recruited & analysed NS  No age/sex/ experience data | 12  Varied l-tryptophan use  Pairs 1 & 2: definite eosinophilia-myalgia  Pairs 3-5: possible eosinophilia-myalgia (pair 3 also varied gender)  Pair 6: polymyositis | N=227 (incl. rheumatologists)  Phase 1 each received 6 scenarios: No. diagnosing eosinophilia-myalgia syndrome:  1a. 51; 1b. 28; 2a. 30; 2b. 6; 3a. 19; 3b. 5; 4a. 14; 4b. 0; 5a. 11 5b. 2; 6a. 7; 6b. 0 | Guidelines used to create 2 scenario pairs representing definite eosinophilia-myalgia (cases 1a-2b); 3 possible eosinophilia-myalgia (3a-5b); 1 polymyositis (6a & 6b): these diagnoses were appropriate decisions  No. diagnosing eosinophilia-myalgia given, but total N not specified |
| Wahlstrom, 2002, The Netherlands & Norway & Germany & Sweden & Slovak Republic [87] | Questionnaire survey  Identify influences on treatment variation, & assess deviations from guidelines on drug therapy of asthma | 274 recruited & analysed  79 Netherlands (11% F), 77 Norway (21% F), 20 Germany (22% F), 98 Sweden (41% F)  (Slovak Republic: only lung specialists/ allergologists recruited) | Sweden: 26  Others: 18  Set 1: varied age, symptoms, body temp, peak expiratory flow measurement  Set 2: varied age, current use of B2-agonist, treatment level with inhaled corticosteroids, symptoms, peak expiratory flow | Decisions of all within a country pooled case-wise, to obtain proportion of yes answers  Netherlands & Norway & Germany & Sweden:  Set 1: Mean proportion decisions to prescribe oral corticosteroid: 0.669 & 0.500 & 0.350 & 0.557  Mean proportion decisions to prescribe antibiotic: 0.343 & 0.490 & 0.550 & 0.469  Set 2: Mean proportion decisions to increase inhaled corticosteroids treatment: 0.495 &0.584 & 0.474 & 0.489 | Used guidelines to determine appropriate decisions  Netherlands & Norway & Germany & Sweden:  Scenario set 1: mean 33% & 33% & 33% & 35% appropriate antibiotic prescribing decisions  Scenario set 2: mean range appropriate inhaled corticosteroid prescribing decisions: 50%-61% & 50%-61% & 50%-61% & 46%-54% |
| Walton, 1997, UK [88] | Crossover experiment with balanced block design  Examine potential effects of computer support in general practice & determine most effective way of presenting advice | 42 recruited & analysed  No age/sex/ experience data | 36  Described medical history, social history, presenting problem, current treatment | % different drug types prescribed:  3% cardiovascular system; 14% central nervous system; 6% respiratory; 19% gastrointestinal; 3%; endocrine; 14% musculoskeletal; 4% all others | 2 clinical pharmacologists, 2 GPs, & 1 pharmacist agreed appropriate decisions per scenario; used this to calculate quality scores (max 8):  Drug choice: 4=top of panel's list; 3=2nd; 2=3rd; 1=4th/lower/not on list but effective & safe; 0= ineffective/unsafe  Dose/frequency: 2=same as panel; 1=not same but effective & safe; 0=ineffective/unsafe  Duration: 2=same as panel; 1=not same but effective & safe; 0=ineffective/unsafe  12 scenarios responded to in control condition: median (range) prescribing score: 6.0 (4.2-7.0) |
| Wigton, 2008, USA [89] | Questionnaire survey  Examine influences on antibiotic prescribing decisions for respiratory tract infection & compare decisions with guidelines | No. recruited & analysed NS  No age/sex/ experience data | 20  Included duration & severity of illness, nasal drainage, productive cough, sinus symptoms, temp, antibiotics expectation, pending trip, prior antibiotics | N= 101 (incl. paediatricians, physician assistants, & nurse practitioners):  Decided likelihood of prescribing antibiotics (0 not at all likely-100% very likely)  Mean likelihood: 43.6, median: 40, inter-quartile range 10–77.5 | Panel of general internists applied guidelines to scenarios & determined whether prescribing antibiotics was appropriate: explicitly instructed to follow guidelines & not use own clinical judgement  Expert panel prescribed antibiotics in 20% cases, participants in 46% |

**Notes:** *CHD= coronary heart disease; COPD=chronic obstructive pulmonary disease; F=female; GP= General Practitioner (used to represent any variant in terminology used e.g. primary care physician /family physician etc.); HBOC=hereditary breast & ovarian cancer; HRT=hormone replacement therapy; M=male; NS=not specified; RCT=randomised controlled trial*; *URTI=upper respiratory tract infection; UTI=urinary tract infection*

*^a^Unless otherwise specified, participants responded to all scenarios;* **^b^***written scenarios unless otherwise specified;* ^c^*where ‘no basic decisional data’ is stated, studies did not report any descriptive statistics in relation to decisions made in response to scenarios;* ^d^*Study also run in USA, but this data not extracted as participants were residents and attendings, not GPs*

*Eighty-six studies which did not assess decision appropriateness*

| **First author, year, country** | **Study design & aims(s)** | **GP participants** | **Scenarios^a,b^** | **Decisions & findings^c^** |
| --- | --- | --- | --- | --- |
| Arnold, 2005, Canada [90] | Questionnaire survey  Examine influences on antibiotic prescribing for paediatric URTI | 119 recruited & analysed  60 M, 59 F  25 0-5 years’ experience; 27 6-10; 22 11-15; 19 16-20; 26 >20 | 16  Children with signs & symptoms of viral URTI | N=250 (incl. 131 paediatricians):  Likelihood of prescribing an antibiotic ( highly unlikely-highly likely): 163 prescribed for at least 1 scenario  Overall prescribing rate 14.5% for GPs |
| Bedson, 2003, UK [91] | Questionnaire survey  Estimate how - rays used in knee pain management in older adults, & investigate influences on management | 447 recruited & analysed  No age/sex/experience data | 4  Patients in mid-60s, 2 had signs & symptoms of clinical osteoarthritis; 2 had pain in absence of these (chronic simple knee pain) | 106 x-rayed all patients, 64 none, 249 1+ but not all  15 would x ray only clinical osteoarthritis scenarios; 15 would x ray only simple knee pain scenarios |
| Belue, 2004, USA [92] | Questionnaire survey  Assess extent of wish for patient involvement in deciding between two treatments & influences on decisions | 40 recruited & analysed  No age/sex/experience data | Average 8 scenarios per participant  Patient with 2-vessel coronary artery disease eligible for either procedure | N=50 (Incl. 10 cardiologists):  Chose coronary artery bypass graft/percutaneous translumial coronary angioplasty/ unsure: 8 chose treatment with lowest mortality/morbidity risk despite patient preference for other |
| Berg, 1991, USA [93] | Questionnaire survey  Determine variation in diagnosis & management of UTIs in women | 135 recruited & analysed  78% M, 22% F  Modal age 39 | 3  Women with differing UTI symptoms & duration | Diagnostic testing: Scenario 1: 94% chose microscopic urinalysis, Scenario 2: 93% chose urinalysis, Scenario 3: 98% chose pelvic examination |
| Bernstein, 1981, USA [94] | Questionnaire survey  Assess influence of patient's gender & expression of personal problem on physician attitudes | 253 recruited & analysed  225 M, 28 F  Mean age: 45 for M, 41 for F | 8; each received 2  F with low back pain/ epigastric pain & M with opposite complaint | % prescribing pain medication & psychoactive drugs:  Abdominal pain scenarios: 6% & 18%  Back pain scenarios: 40% & 13% |
| Bierma-Zeinstra, 2000, The Netherlands [95] | Questionnaire survey within observational study  Investigate influences on & consistency of hip problem management | 20 recruited & analysed  15 M, 5 F | 4  Included medical history, present complaint hip pain, abnormal findings | Management after 3 repeated consultations:  Cumulative % selecting each strategy:  Scenario 1: 95% x-ray  Scenario 2 & 4: 90% & 95% orthopaedic surgeon  Scenario 3: 90% physical therapy |
| Bitzer, 2009, Switzerland [96] | Questionnaire survey  Assess whether & which criteria used to prescribe 20 & 30 µg combined oral contraceptives with the same progestogen | 25 recruited, 20 analysed  No age/sex/experience data | Scenario no. unclear  Included general characteristics, risks & pre-existing complaints | N=158 (incl. 138 gynaecologists):  Prescribing: considered 2:1 ratio in favour of either treatment clinically relevant  20 µg prescribed in at least 2:1 ratio with age > 35, venous thromboembolic disease, breast tenderness, headache history, smoke > 15/day  30 µg prescribed in at least 2:1 ratio with acne history, body mass index <18.5, poor medication compliance, menstrual cycle irregularities, family history of osteoporosis |
| G:Borkhoff, 2009, Canada [97] | Questionnaire survey within standardised patient study  Compare management of total knee arthroplasty for standardized patients with matching scenarios, & determine influence of patient gender on decisions | 123 recruited, 119 analysed  No age/sex/experience data | 8  Described presenting problem (chronic right knee pain) & symptoms consistent with moderate knee osteoarthritis | N=180 (incl 61 orthopaedic surgeons):  Group 1 (visited by standardized patients, N=60); 75% recommended total knee arthroplasty  group 2 (not visited, N=120): 80% recommended total knee arthroplasty |
| Bremberg, 2003 & 2005, Sweden & Slovakia [98, 99] | Questionnaire survey  Compare different groups views of patients reluctant to accept medically motivated action/ demanding something not medically motivated | 220 recruited & analysed  110 M, 109 F  121 aged <=50, 98 >50 | 4  Healthy, reluctant to quit smoking/ demanding pulmonary x-ray/pulmonary cancer, reluctant to quit smoking/demanding immunotherapy | Scenario 1 (N=219): 176 would/probably would discuss smoking again  2 (N=215): 119 absolutely or probably would not refer for new pulmonary x-ray  3 (N=218): 126 would/probably would discuss smoking again  4 (N=216): 117 absolutely or probably would not help patient get immunotherapy |
| Bryant-Waugh, 1992, UK [100] | Questionnaire survey  Determine extent to which anorexia nervosa & eating disorder in general considered possible diagnoses in children & effects on management | 46 recruited & analysed  No age/sex/experience data | 2  11-year-old girl/boy, symptoms of anorexia | Scenario 1 & 2:  6 & 9 diagnosed anorexia; 1 & 1 eating related disorder; 15 & 30 other psychiatric  Before urine & blood test results proved normal: 49 & 46 would refer  After: 63 & 67 would refer |
| Burgess, 2008, & Phelan, 2009, USA [101, 102] | Postal questionnaire survey  Identify patterns in treatment decisions for non-malignant chronic pain, & influences on decisions | 382 recruited, 381 analysed  112 F  Mean age 45 years | 24; each received 1  Patients black/white, younger/ older, appear confident/ angry/ dejected, verbal behaviour challenging/ nonchallenging  All male  Symptoms, functional status, medical history & physical exam results equivalent  Sequence of photographs & written dialogue | 63% would order further diagnostic testing; 62% question about drugs/alcohol; 60% perform functional assessment; 57% discuss emotional issues; 56% pain clinic/specialist referral; 50% physical therapy referral |
| Campbell, 2006, UK [103] | Factorial experiment within questionnaire survey  Examine influences on sick note offer | 489 recruited & analysed  328 M, 153 F  Years’ experience: 74 0–5; 98 6–10; 175 11–20; 108 21–30; 28 >30; 6 no data | 8; each received 1  Varied patient request, family issues, & problem | Rated agreement with giving sick note because patient needs one/deserves one/to maintain good relationship  Responses summed & divided by 3 to create total score  Mean total scores:  Scenario 1 (N= 62): psychological problem: 2.99  2 (N=65): psychological problem & request: 2.95  3 (N=60): psychological problem & family issues: 3.17  4 (N=56): psychological problem & request & issues: 3.20  5 (N= 58): physical problem: 2.75  6 (N=66): physical problem & request: 2.90  7 (N=62): physical problem & issues: 2.87  8 (N=60): physical problem & request & issues: 2.93 |
| Carey, 1996, USA [104] | Questionnaire survey within prospective cohort study  Examine influences on lumbar spine radiography & computed tomography/ magnetic resonance imaging for acute low back pain | No. recruited & analysed NS  No age/sex/experience data | 3  Sciatica & diminished ankle reflex/chronic back pain/acute back pain & normal results on physical examination | N=85 (incl. other health-care professionals):  Scenarios 1 & 2 & 3: 35% & 26% & 11% would order computed tomography/magnetic resonance imaging |
| Carr, 1994, Australia [105] | questionnaire survey  Examine whether inaccurate knowledge about sore throat features & management influences inappropriate antibiotic prescribing | 284 recruited & analysed  214 M of 270  82 of 271 had > 25 years’ experience | 4  Tonsillitis, child/ probable viral URTI, adult/tonsillitis or glandular fever, adult/ probable viral URTI, child | No. prescribing (N=274 for scenarios 1-3, & 275 for scenario 4):  Scenario 1: 266; 2: 26; 3: 191; 4: 79  No. using throat swab:  Scenario 1(N=274): 35  2 (N=273): 3  3 (N=271): 62  4 (N=272): 19 |
| Chapman, 2001, Canada & USA [106] | Interview survey  Determine if similar provisional diagnoses reached & tests ordered for patients with identical chronic respiratory symptoms & smoking histories differing in gender | 192 recruited & analysed  154 M, 38 F | 6; each received 1  Former smoker, morning cough worsening following URTIs, breathlessness on moderate exertion, expiratory wheezes throughout chest  M/F, age 47/59, Forced Vital Capacity, Forced Expiratory Volume 1 52%/32%, Forced Expiratory Volume 1/ Forced Vital Capacity 43%/27%  Also included laboratory test results & negative oral steroid trial results | Most common diagnoses was COPD at all 3 scenario stages:  Basic scenario: 65% diagnoses for M scenarios & 49% for F  Test results given: 76% for M scenarios & 65% for F  Trial results given: 85% for M scenarios & 78% for F  78% ordered chest radiograph |
| Chaput de Saintonge, 1981, UK [107] | Questionnaire survey, pilot study  Examine usefulness of scenarios for auditing antibiotic prescribing for otitis media | 7 recruited & analysed  Years’ experience range 7-22 | 89  Children with painful ears: all included history, signs & symptoms  48 included ear drum description  41 included ear drum photograph | Assessed agreement between decisions made for actual cases & in response to scenarios using Cohen's weighted Kappa: found significant agreement (p<.005) between diagnoses reached in life & scenarios  Antibiotic prescribing decisions also showed good agreement |
| Chaput de Saintonge, 1985, UK [108] | Questionnaire survey  Determine whether differences exist in diagnosis & management of bacterial otitis media & whether differences could be resolved by informing participants of the underlying basis of their decisions | 4 recruited & analysed  No age/sex/experience data | 50  Patients with otitis media  Certain history, examination & clinical variables present/absent | N=5 (incl. 1 trainee):  Bacterial otitis media diagnosed in 28%-80% cases  Antibiotics prescribed for 41%-100% cases |
| Cicutto, 2000, Canada [109] | Questionnaire survey  Determine treatment approaches to adult outpatient asthma of varying severity & clinical characteristics | 80 recruited & analysed  61 M, 19 F  Years’ experience: 12 0-10; 40 11-20; 28 >20 | 6  Triggers of asthma symptoms, degree of asthma symptoms, use of medications  3 severity levels: mild (scenario 1); moderate (scenarios 2 & 5 & 6); severe (scenarios 3 & 4) | Modified RAND method: area of agreement identified when at least 75% would recommend/not recommend a treatment  Areas of agreement:  Scenario 1: 98% no theophylline  2 & 5 & 6: 100% no wait & see  3: 99% no wait & see  4: 100% no wait & see |
| Currin, 2007 & Currin, 2009, UK [110, 111] | Questionnaire survey  Investigate influences on decisions about diagnosis & treatment of eating disorders | 82 recruited & analysed  44 F | 16; each received 2  Varied gender, ethnicity, body mass index, presence of co-morbid medical condition  Scenario 1. Underweight, restricts food intake, regimented exercise, presenting complaint: trouble sleeping & lack of concentration  2. Overweight, difficulty managing anger, distress & loss of control during binge eating sessions, occurring 4-6 times/week | Scenario 1: 47/82 received primary diagnosis of anorexia nervosa  2: 60/82 received eating disorder diagnosis  Most popular treatment choice: follow-up appointment (79% scenario 1; 70% scenario 2)  Mean proposed time between initial presentation & follow-up appointment: 2.6 weeks |
| Davidson, 1986, USA [112] | Questionnaire survey  Investigate effect of uncertainty in selection of therapies for hypertension on treatment | No. recruited & analysed NS  No age/sex/experience data | 4  Hypertension, varied race, sex & SES | N=95 (incl. other specialties):  Diuretics most frequently chosen, range 55% to 29% across scenarios  Beta-blocking agents: range 9% to 40% across scenarios |
| de Bock, 1999, The Netherlands [113] | Interview survey  Examine whether multiattribute utility scores correctly reflect management decisions for patients with suspected sinusitis | 80 recruited & analysed  No age/sex/experience data | 3  Symptoms suggestive of acute maxillary sinusitis/symptoms not clearly related to sinusitis/symptoms related to rhinitis | Analgesic &/or local therapy prescribed by 14 (scenario 1), 74 (scenario 2) & 83 (scenario 3)  Antibiotic prescribed by 86 & 23 & 2  3 (scenario 2) & 15 (scenario 3) waited & provided information |
| Diette, 2007, USA [114] | Questionnaire survey  Examine influences on asthma treatment decisions | 225 recruited & analysed  69% M, 31% F | 9; each received 4  Scenario 1: baseline for stepping up treatment  2-5: receiving low-intensity therapy & persistent symptoms/ intermittent symptoms & hospitalized 6 months ago/intermittent symptoms & patient bothered by asthma/ intermittent symptoms & symptoms worse than at previous visit  6: baseline for stepping down treatment  7-9: intermittent symptoms & on a stable regimen for past 6 months &: hospitalized in the past 6 months/ patient bothered by asthma/symptoms worse than at last visit | Scenario 1: Most did not step up treatment  % stepping up treatment:  2: 97%; 3: 72%; 4: 80%; 5: 68%  6: 29% would step down treatment  % stepping down treatment:  7: 11%; 8: 8%; 9: 2% |
| Doust, 2000, Australia [115] | Questionnaire survey  Investigate interpretation of systematic review evidence challenging routine use of antibiotics for otitis media in children | 51 recruited & analysed  29 M  Mean 22 years’ experience | 3  7 -year-old with unilateral signs & symptoms consistent with otitis media < 24 hours duration/3 -year-old with fever & irritability for 3 days/2 -year-old with fever, clear nasal discharge & pulling at ear for 2 days | N=62 (incl. 11 professors/head of depts. of general practice):  Physicians with critical appraisal training (N=19) & physicians without (N=43):  Probability of prescribing antibiotics marked on visual analogue scale, 0% never- 100% always:  Scenario 1: mean probability 43% (range 0-100%) & 77% (19-100%)  2. 70% (0-100%) & 88% (41-100%)  3. 43% (0-100%) & 82% (21-100%) |
| Elson, 1997, USA [116] | Simulated decision-making experiment using controlled parallel experimental design  Investigate impact of summarized patient data display on decisions based on serum lipid test results | 4 recruited & analysed  Aged 35 (x2)/36/38 | 8; each received 4  Included current & prior lipid values; copy of actual index lab report from reference laboratory  Of the four charts, two had summary of patient data of relevance to lipid management (intervention), two did not (control) | Made recommendations related to diet, medication, & further lab testing  No basic decisional data |
| Epstein, 2008 & Hooper, 2008, USA [117, 118] | Factorial experiment within interview survey  Examine characteristics associated with depression decisions | 418 recruited, 404 analysed  248 M, 156 F  Mean age 48 | 32; each received 1  Varied race, gender, medical illness, attribution of cause of depression, willingness to receive mental health treatment  Mid-50s, major depressive episode | 395 had depression as a leading diagnosis  239 ordered thyroid function tests  347 recommended antidepressant; 88 physicians counselling; 93 exercise  189 referred to mental health specialist |
| Eva, 2010 & Ketcham, 2009, & Lutfey,2010, USA [119-121] | Factorial experiment within interview survey  Examine influences on diagnostic flexibility & diagnosis & treatment of CHD | 256 recruited & analysed  50% F  Mean 18 years’ experience | 16; each received 1  Varied age, gender, race, SES  CHD signs & symptoms | 155 named CHD as most likely diagnosis  94 changed diagnosis from initial impression  Between 2-19 diagnoses generated, mean: 6.3  98% ordered cardiac test  Between 0-7 medications ordered, mean 2.2  Between 0-15 pieces of advice given, mean 4.1  Between 0-42 days to follow-up, mean 8.9 |
| Evans, 1995, & Harries, 1996, UK [122, 123] | Simulated decision-making experiment  Determine tacit & stated policies in treatment/ management of raised blood cholesterol/ migraine/symptoms relevant for HRT, & extent of agreement | 35 recruited, 34 analysed  31 M, 4 F  Mean 9 years in general practice, range 0.5-27 | 390; each received up to 130  Patients with raised blood cholesterol levels/migraine/ symptoms relevant for HRT  Each set contained 13 factors which varied | Likelihood of prescribing: no basic decisional data |
| Farmer, 1992, UK [124] | Interview survey  Evaluate changes in referral practice when characteristics of patients presenting with symptoms of anxiety &/or depression varied | 17 recruited & analysed  Mean 11 years in general practice, range 1-35 | 16  Severity, psychiatric history, psychosocial context, gender, & age varied | 31% ‘cases’ & 12% ‘non-cases’ referred to psychiatrists  For all scenarios with severity level 4+, further treatment (drugs/counselling/ psychotherapy) was given if no referral |
| Finkelstein, 1998, USA [125] | Questionnaire survey  Investigate recommendations for growth hormone therapy for treatment of childhood short stature | No. recruited & analysed NS  No age/sex/experience data | 4  Varied degree of short stature (height 2/3 standard deviations < mean for age), rate of growth (3^rd^-10^th^/< 3^rd^ percentile for age) | % who would refer/likely to refer to paediatric endocrinologist:  Scenario 1 (2 standard deviations <, 3rd-10^th^): 6%  2 (2 Standard deviations <, < 3^rd^): 81%  3 (3 standard deviations <, 3rd-10^th^): 56%  4 (3 standard deviations <, < 3^rd^): 95% |
| Friedmann, 1996, USA [126] | Questionnaire survey  Assess perceptions of drug therapy for hypercholesterolemia & isolated systolic hypertension & coronary artery bypass graft for stable angina & left main coronary stenosis | 161 recruited & analysed  84 family physicians & 77 Internists:  79%/79% M  13%/21% aged <35, 42%/36% 35-44, 17%/21% 45-54, 15%/ 9% 55-64, 13%/13% >=65 | 3  Hypercholesterolemia/ isolated systolic hypertension/chronic stable angina with left main coronary stenosis | Family physicians & internists:  Scenario 1: drug treatment recommended by 84% & 88%  2: drug treatment recommended by 82% & 90%  3: coronary artery bypass graft recommended by 45% & 556% |
| Gagnon, 2008, Canada [127] | Questionnaire survey  Examine treatment of subclinical hypothyroidism & hyperthyroidism & influences on treatment | 141 recruited, 130 analysed  Majority had > 20 years’ experience | 4  25-year-old F/70-year-old F/30-year-old F, lost weight, palpitations, intolerance of heat , undetectably low thyroid stimulating hormone, slightly elevated thyroxine, results compatible with Graves’ Disease/66-year-old F, osteopenia, subclinical hyperthyroidism, results compatible with multinodular goiter  Levels of thyroid stimulating hormone provided | Scenario 1: 42% chose a treatment option (i.e. did not choose observation)  2: 48% chose a treatment option  3: 88% referred to specialist  4: 86% referred to specialist  (Percentages for scenarios 1 & 2 are approximate, taken from figure) |
| Gerbert , 1984, USA [128] | Questionnaire survey  Determine whether patient likeability & competence influence treatment | 61 recruited & analysed  No age/sex/experience data | 18; each received 6 (1 diabetes, hypertension & asthma video & written scenario)  Patient likeable-competent/unlikeable- competent /likeable-incompetent | N=93 (incl. 3 paediatricians; 2 emergency physicians; 1 preventive medicine specialist; 26 specialty unspecified):  Decisions regarding nine dimensions for treatment analysed using series of nine, three-by-three Iatin square analyses:  For 5/9 scales, treatment varied depending on likeability & competence |
| Grant, 2009, USA [129] | Factorial experiment within interview survey  Investigate influences on tendency to intensify medical therapy | 192 recruited & analysed  No age/sex/experience data | 24; each received 1  Patient with type 2 diabetes & initial symptoms of foot neuropathy  Varied age sex, race, SES | 53% would recommend a diabetes- related medication prescription: 49% antihypertensive therapy, 25% antiglycemic therapy, 15% lipid-lowering therapy; 47% would not intensify treatment |
| Gupta, 2009, Australia [130] | Questionnaire survey  Identify methods used to assess cardiovascular risk, accuracy of assessments, & describe strategies used for management of medical & lifestyle risk factors | 197 recruited & analysed  67% M  Mean 18 years’ experience | 6  Included gender, age, cholesterol  Some included body mass index, smoking status, blood pressure, screening tests, positive family history of heart disease, diabetes | Provided 6 month management plan: up to three strategies organised in order of priority  Most frequent first lines of management:  Scenario 1: Stop smoking, chosen by 123/194  2 & 6: Treat blood pressure, 101/191 & 102/188  3 & 4: Reduce cholesterol: 168/191 & 103/192  5: Stabilise diabetes: 80/191 |
| Haggerty,2005 & Tudiver, 2002, Canada [131, 132] | Questionnaire survey  Compare influences on ordering cancer screening tests where guidelines equivocal | 351 recruited & analysed  65% M  Mean 16 years’ experience | 64; each received 4  Varied cancer anxiety, test expected, cancer family history, easy/difficult patient-physician relationship  2 scenarios on prostate cancer screening in men > 50 years; 2 on breast cancer screening in women 40-49 years; 2 on colorectal cancer screening in adults >40 | For each scenario, test more likely to be ordered when patients anxious about cancer/expected to have test; test most likely to be ordered when both anxiety & expectation present |
| Halpin, 2007, UK [133] | Questionnaire survey  Assess confidence in diagnosing COPD & differentiating between asthma & COPD | 85 recruited & analysed  (46 in 2001, 39 in 2005) | 4  Asthmatic as child, ex-smoker, myocardial infarction, breathless, ankle swelling/short of breath, history of hypertension, smokes 40 a day, on HRT/asthmatic, breathless, ex-smoker/chest tightness, difficulty playing golf, was semi-professional footballer, smokes 20 a day | N= 39 (participants assessed in 2005):  1: COPD diagnosed by 51%; of these, 50% ordered chest x-ray  2: COPD diagnosed by 74%; of these, 54% ordered chest x-ray  3: COPD diagnosed by 62%; of these, 64% ordered chest x-ray  4: Angina 49% |
| Halvorsen, 2011, Norway & Poland [134] | Questionnaire survey  Explore whether frequency & duration of sick-leave certification for acute airways infections differ between countries | 387 recruited & analysed  216 Poland & 171 Norway  66% & 45% F  Mean 12 years’ experience | 4  Patients with different types of acute airway infection  Symptoms of pneumonia/sinusitis/ common cold/ exacerbation of COPD | % offering a sick leave note: 98% for pneumonia, 92% COPD, 89% sinusitis, 75% common cold  Mean duration of sick note varied from 8 days for pneumonia to about 4.5 days for common cold |
| Hamilton-Craig, 2000, UK [135] | Questionnaire survey  Determine which factors most influence prescribing & treatment of moderate hypertension | 110 recruited & analysed  39% F  Mean age 42 years | 5  Included age, blood pressure, cholesterol, diabetic & smoking status, resulting in patients with 5-year absolute risks of cardiovascular disease from <5% to >20% | ACE inhibitors comprised 45% of prescriptions, diuretics 30%, beta-blockers 17%, calcium channel blockers 8%  Diabetic patients more likely to receive ACE inhibitors than others |
| Harries, 2007, UK [136] | Questionnaire survey  Examine whether & how patient age influences decisions for angina | 28 recruited & analysed  17 F | 72  Varied gender, age, occupational status, ethnicity & clinical information  Patients presented with chest discomfort | Made investigation & treatment decisions: no basic decisional data |
| Hart, 1997, Israel [137] | Questionnaire survey  Examine influence of pharmaceutical cost on decisions about drug prescription for UTI | 35 recruited & analysed  Experience in family medicine range 2-30 years, mean 12 | 2  Patients with mild/severe UTI  Included age, gender, clinical features, lab findings | Drug to prescribe chosen before being aware of drug cost, then after 2 months with price of drugs shown  Mild UTI: 60% chose Trimethoprim/ sulfamethoxazole at both phases  Severe UTI: 74% gentamycin, 22% ceftriaxone/ cefotaxime at both phases |
| Hartley, 1985, UK [138] | Questionnaire survey  Examine use of laboratory testing by comparing responses to scenarios with actual behaviour | 20 recruited, 19 analysed  No age/sex/experience data | 15  Patients with problems typical of general practice  Included presenting complaints & examination findings | Total no. tests ordered per GP ranged from 13 - 26  Also made diagnosis, prescribing, referral & follow-up decisions: no basic decisional data |
| Horne, 1999, UK [139] | Interview survey  Establish management intentions for heart failure & identify barriers to implementing evidence-based guidelines | 100 recruited & analysed  2% aged <=30, 36% 30-39, 35% 40-49, 21% 50-59, 6% 60-69 | 3  Typical congestive heart failure/more unusual pathology & more complex symptomatology | Typical case: 30% referred; of 70 not referring, 54% chose chest X-ray, 30% electrocardiogram, 20% echocardiogram, 16% measure haemoglobin  Complex cases: 73% & 38% referred  Diuretic therapy preferred treatment for all cases |
| Howie, 1974, UK [140] | Interview survey  Examine respiratory illness consultation in general practice | 20 recruited & analysed  Experience range, 5-25 years | 10  Patients with various symptoms of respiratory illness  4 small children, 4 young adults, 2 middle aged, each differed on 1/more significant features | URTI mentioned as diagnosis for scenarios 1-6; coryza for scenarios 1-4 & 6; tonsillitis for scenarios 3, 4, 5, & 7  Antibiotic prescribed for 132/200 scenarios; 80% consensus for prescribing for scenarios 1, 4, & 7-10, & against for scenarios 2 & 5; scenarios 3 & 6 had majority views against (12:8 & 13:7) |
| Hrisos, 2008, UK [141] | Questionnaire survey within RCT  Evaluate whether graded task with action planning & a persuasive communication intervention influence decisions to manage URTI without prescribing antibiotics | 340 recruited & analysed  56% M  Mean 20 years’ experience | 16 (8 pre- & 8 post-intervention)  Included name, address, age, active problems, smoking status, significant past, occupation, current medication, history, examination | Calculated total no. scenarios per participant for which antibiotics not prescribed:  Mean scores pre- & post-intervention:  Those who received graded task intervention (N=164): 5.40 & 5.10  Those who did not (N=176): 5.36 & 4.97  Those who received persuasive communication intervention (N=164): 5.35 & 5.25  Those who did not (N=176): 5.41; 4.83  Also made diagnosis & other management decisions: no basic decisional data |
| Jenkins, 1988, UK [142] | Questionnaire/interview survey  Identify extent to which International Classification of Diseases & International Classification of Health Problems in Primary Care can be applied consistently & identify stages in decision making where diagnostic variation occurs | 27 recruited & analysed  All qualified between 1945-1975 | 8  3 videos: selected from a library, contained prominent psychological/ emotional component  5 written: had psychological component | Scenario 1: Over two-thirds recommended further exploration & psychotherapy  2 (N=26): Over 90% recommended further exploration  3 (N=26): Two-thirds proposed further exploration  4 (N=20): 80% agreement for social worker referral  5 (N=20): General agreement for psychotherapy, further exploration, & antidepressant  6 (N=20): Three-quarters recommended antidepressants  7 & 8: no relevant results |
| Kaner, 2001, Australia, Belgium, Bulgaria, Canada, France, Hungary, Italy, New Zealand, Norway, Poland, Portugal, Thailand, UK [143] | Questionnaire/interview survey  Assess extent of postgraduate education & training on alcohol & investigate influence on attitudes to & diagnosis & management of alcohol-related problems | 2139 recruited & analysed  1268 M  Mean 14 years’ experience | 2  Patient drinking excessively, evidence of some health problems, no physical dependence/alcohol consumption & physical symptoms suggestive of alcohol dependence  Scenarios from an earlier study adapted | Scenario 1: 85% would record consumption & advise cutting back; 97% state alcohol is related to problems  2: 83% record consumption & advise abstaining; 99% order blood & liver enzyme tests |
| Kellen, 1998, Canada [144] | Questionnaire survey  Investigate whether warfarin prescription for stroke prevention in elderly patients with atrial fibrillation influenced by physician characteristics | 138 recruited, 91 analysed  69% M  Mean 17 years’ experience | 2  Included age, background information, history, current medication, physical examination, 12-lead electrocardiogram  Scenarios selected from those used at 1994 Canadian Cardiovascular Society Atrial Fibrillation workshop | 62% prescribed warfarin for the elderly female scenario  67% prescribed warfarin for the elderly male scenario |
| Kesten, 1993, Canada [145] | Interview survey  Assess attitudes & management decisions for COPD | 75 recruited & analysed  No age/sex/experience data | 2; each received 1  52-year-old smoker, symptoms suggestive of recent URTI, subsequent cough, expiratory wheezes on chest examination  Varied explicit reference to earlier tentative diagnosis of chronic bronchitis/not | Chest radiographs most frequently ordered, chosen by 80%  Bronchitis/pneumonia most frequently mentioned primary diagnosis, stated by 33%  Oral antibiotics most frequently chosen first choice drug, chosen by 63% |
| Kikano, 1991, USA [146] | Questionnaire survey  Evaluate use of lab tests in the management of febrile infants without a focal source of infection | No. recruited & analysed NS  No age/sex/experience data | 2; each received 1  10 month old infant, 2 day history fever, nonspecific symptoms, no focal findings on physical exam, results of white blood cell count (9000/18000) | N=196 ( incl. paediatricians & emergency physicians):  Initial test ordering: overall mean 1.4 tests ordered  Initial management: overall 11% selected reassurance, 83% antipyretics & fluids, 4% outpatient antibiotics, 2% hospitalisation |
| Kikano, 1996, USA [147] | Questionnaire survey  Determine influence of patient's SES/profession on diagnosis & treatment of certain medical problems | 201 recruited & analysed  87% M  73% aged >45 years | 4; each received 2  Scenarios did/didn’t include SES or profession information  Scenario 1: patient with low back pain, lawyer/not specified  2: patient with a cough, spouse of local hospital chief executive officer/not specified | Scenarios with SES/occupation data:  Scenario 1: 15% chose physical therapy; mean 2.2 tests ordered; 56% would follow-up <= 1 week  2: 45% complete blood cell count with differential; mean 2.3 tests ordered; 59% follow-up <=1 week  Without SES/occupation data:  1: 4% physical therapy; mean 2.2 tests ordered; 38% follow-up <= 1 week  2: 29% complete blood cell count with differential; mean 1.8 tests ordered; 34% follow-up<=1 week |
| Kuder, 1987, USA [148] | Questionnaire survey  Determine variation in admittance of patients with diabetes/chronic hypertension to hospital when best management protocol is uncertain, & factors influencing variation | 78 recruited, 38 analysed  No age/sex/experience data | 24  Varied living arrangements: (lives alone/with daughter), travel time to practice (2 hours/15 mins), extent of financial hardship (no hardship/ financial hardship by paying for out-patient medical costs/ financial hardship for any costs)  Scenario 1: 60-year-old, obesity & diabetes, no major complications  2: 60-year-old, chronic hypertension controlled by diet & diuretics | N=57 (incl. 19 surgeons/other specialties):  Mean ratings for likelihood of admitting to hospital (very unlikely to admit-very likely; placed slash mark on response line & marks transformed into numerical responses by measuring length of the line segment in mm): 1: 20.6 2: 70.9 |
| Little, 1998, USA [149] | Interview survey  Identify clinical factors strongly influencing sinusitis diagnosis & investigate extent to which x-ray & transillumination used to diagnose sinusitis | 40 recruited & analysed  Years’ experience range 2-46 years, median 14 years | 128; each received 16  Adult patient with rhinorrhea  7 clinical factors (present/absent): history of coloured nasal discharge; maxillary or facial pain; failure to respond to decongestants; purulent drainage on nasal examination; sinus tenderness on examination; fever on examination; smoking history | Total 640 cases: 180 (28%) classed as low probability sinusitis; 283 (44%) intermediate; 177 (28%) high  Antibiotics prescribed for 406 (63%)  Cases with <=2 factors present: 2% classed as high probability, antibiotic prescribed for 3%  Cases with >=5 factors present: 72% classed as high probability, antibiotic prescribed for 99% |
| Lynggaard, 2006, Denmark [150] | Questionnaire survey  Explore decision process leading to starting or withholding treatment in hypertensive patients | 55 recruited & analysed  67% M  Median age 51 years | 5  F, aged 58, diabetes, smoker, cholesterol 9.0, blood pressure 150/98, risk category 1/F, aged 74, no diabetes, smoker, cholesterol 4.4, blood pressure 180/84, risk category 2/M, aged 50, diabetes, non-smoker, cholesterol 6.6, blood pressure 162/92, risk category 3/F, aged 52, no diabetes, non-smoker, cholesterol 7.2, blood pressure 150/95, risk category 4/M, aged 35, no diabetes, non-smoker, cholesterol 5.0, blood pressure 158/96, risk category 5  Adapted from Hamilton-Craig et al (2000) | % choosing to treat for each scenario: 1: 96% 2: 85% 3: 96% 4: 56% 5: 63%  Most frequently chosen drugs for first-line monotherapy were ACE inhibitors, angiotensin 2 receptor blockers & diuretics |
| Matthews, 1993, UK [151] | Questionnaire survey  Elicit attitudes, experiences & influences on antidepressant drug use | 231 recruited & analysed  180 M, 47 F  Mean 11 years as a GP | 3  Reactive or neurotic depression/endogenous depression/panic disorder or phobic neurosis | 47% prescribed for scenario 1, 78% for 2, 68% for 3  29 chose drug & non-drug measures for 1, 41 for 2, 75 for 3 |
| McCranie, 1978, USA [152] | Questionnaire survey  Assess effects of gender-role stereotyping on diagnostic inferences | 117 recruited & analysed  Mean 21 years in practice | 4; each received 1  M/F versions of 2 scenarios  Scenario 1: chronic headaches, generalized weakness, malaise, stiff neck, tinnitus, occasional nausea with headaches, decreased visual acuity, lost interest in sex, weight loss, occasional insomnia  2: chronic abdominal pain, increased flatus, intermittent diarrhoea, frequent bilateral headaches, occasional stiffness in neck & shoulders, occasional lower backache, frequent heartburn | Number of psychogenic & organic diagnoses:  M headache (N=27): 7 & 19  F headache (N=25): 4 & 21  M abdominal pain (N=32): 2 & 30  F abdominal pain (N=33):4 & 28  Few ordered psychological tests  Few prescribed psychological treatment; psychotropic medication mentioned more frequently  Few requested psychiatric consultation |
| Modi, 2007, USA [153] | Questionnaire survey  Examine whether tube feeding recommended more often for African American than Caucasian patients, all else equal, & identify influences on decisions | 1083 recruited, 981 analysed  75% M, 25% F  33% 0-10 years’ experience, 32% 11–20, 36% >20 | 2; each received 1  Caucasian/African American elderly F with advanced dementia & weight loss presenting for routine follow-up with daughter | 18% recommended feeding tube placement, 82% recommended against/made no recommendation  16% of those receiving Caucasian patient recommended tube placement, compared to 20% receiving African American patient |
| Montgomery, 2006, Ireland [154] | Interview survey  Examine influences on referral for renal replacement therapy | 51 recruited & analysed  43 M, 8 F  Mean age 50 years | 32  Different versions of a moderate case and a severe case: creatinine and urea levels varied  Also differed in age, single/married, inactive rheumatoid arthritis/no co-morbidity | Moderate case: 811 decisions: visit 1: 28% referred to nephrologist, 47% not referred: visit 2: of this 47%, 18% referred to nephrologist  Severe case: 821 decisions: visit 1: 28% referred to nephrologist, 45% not referred: visit 2: of this 45%, 14% referred to nephrologist  Nephrologist referral decreased in likelihood as scenarios became more complex |
| Montori, 2006, Canada & USA [155] | Questionnaire survey  Assess extent to which contraindications affect treatment decision-making, & evaluate possibility that contraindications impair ability to make evidence-based, patient-centred decisions | No. recruited & analysed NS  No age/sex/experience data | 2; each received 1  Scenario 1: diabetes, creatinine level exceeding contraindicated threshold for metformin, unwilling to use antihyperglycemic agents, informed & strong preference against insulin  2: difficult to control migraines, eager to use triptans, coronary artery disease (contraindication to triptan use) | N= 466 (incl. other specialties):  After all information given (scenario details & contraindication pointed out & weak evidence supporting contraindication): 164/236 (scenario 1) & 144/209 (scenario 2) didn’t prescribe contraindicated medication  For each scenario, proportion offering contraindicated intervention decreased after second stage relative to first & increased after third stage relative to second but lower than first |
| Morrell, 1990, UK [156] | Questionnaire survey  Validate indirect measure of referral behaviour by examining association between scenario responses & actual referral behaviour | 18 recruited & analysed  No age/sex/experience data | 21  One part revealed at a time: participant made referral decision: if yes, moved on to next scenario: if no, received next piece of information & decided again: total no. of steps ranged from 3-6  Some became more serious, others had increasing patient pressure for referral | N=20 (incl. 2 trainees):  Referral decision scored by adding number of steps at which participant decided not to refer & subtracting from total number of steps:  Mean score = 38, range 24-48 (maximum possible range 0-107) |
| Neuner , 2007 & 2012, USA [157, 158] | Questionnaire survey  Evaluate osteoporosis management & determine whether quantitative hip fracture risk estimates used for postmenopausal osteoporosis prevention/ treatment & assess influences on treatment decisions | No. recruited & analysed NS  No age/sex/experience data | 4  Varied on age, weight, bone density, 5-year & lifetime estimates of hip fracture risk  All white postmenopausal women, lifelong non-smoker, no personal/ family history of fractures  Basic bone density report/augmented report with 5-year & lifetime risks | N=287 (incl. obstetrics & gynaecology specialists):  Over 96% prescribed calcium & vitamin D for all scenarios |
| Nikolajevic-Sarunac , 1999, Australia [159] | Questionnaire survey within RCT  Investigate effect of information framing on prescribing long-term HRT & extent to which framing effects varied across scenarios | 243 recruited phase 1, 215 phase 2; 215 analysed  Of 243 recruited: 149 M  Group A (N=81) & group B (N=82) & control group (N=80):  Median 14 & 12 & 12 years in general practice | 7  Postmenopausal women, no mention hysterectomy, none had preference for/against HRT, all would comply with decision  Scenarios had different risks for hip fracture, myocardial infarction, and/or breast cancer | Intention to prescribe long-term HRT measured on 7-point Likert scale (definitely no-definitely yes):  In 6 scenarios, trend for control & group A to become more likely to prescribe HRT; group B highest proportion less likely to prescribe & lowest proportion more likely to prescribe |
| Petitti, 1993, USA [160] | Questionnaire survey  Determine extent of variation in recommendations about revisit interval for 3 common chronic conditions & determine associated physician characteristics | 116 recruited & analysed  Mostly male & middle-aged | 3  Diabetes/angina/ hypertension  Included gender, age, history, clinical findings, current medication | 80% were family physician & 20% were internists:  Diabetes: follow-up visit interval <=1 month chosen by 23% & 0%; 2-4 months 66% & 78%; >= 6 months 11% & 22%  Angina: <=1 month 12% & 12%; 2-4 months 72% & 82%; >= 6 months 16% & 6%  Hypertension: <=1 month 23% & 0%; 2-4 months 66% & 95%; >= 6 months 11% & 6% |
| Potter, 2001, USA [161] | Questionnaire survey  Determine attitudes & practices regarding use of opioids to treat chronic nonmalignant pain | 161 recruited & analysed  67% M  Mean age 47 | 3  45 -year-old F, architect, severe low back pain, persistent after extensive work up & treatment /66 -year-old M, postherpetic neuralgia, new patient, tried multiple oral & topical medications to alleviate pain/37 -year-old F, chronic daily headaches, extensive workup & tried multiple medications | % referring:  Scenario 1: 56% 2: 32% 3: 40%  % would treat with opioid:  1: 38% 2: 80% 3: 40%  % agree/strongly agree to treat with long-acting opioids if pain persisted:  1: 28% 2: 58% 3: 20% |
| Rathore , 2009, USA [162] | Questionnaire survey  Assess effect of patient race on treatment decisions for hypertension, hypercholesterolemia, & diabetes mellitus | 716 recruited & analysed  31% F | Each received 3  Varied race, treatment adherence, drug insurance source, age  Included history, relevant current medications, findings from exam & patient photo (initially used by Schulman et al (1999)) | Most common treatment:  Hypercholesterolemia: 99.7% recommended any treatment; 97.9% statin  Hypertension: 99.6% recommended any treatment; 54.8% ACE inhibitor  Diabetes: 99.7% recommended any treatment; 69.0% Biguanide |
| Roark, 1995, USA [163] | Questionnaire survey  Determine theoretical practice patterns for persistent & recurrent otitis media | 142 recruited & analysed  No age/sex/experience data | 2  13-month-old boy, acute otitis media treated for 10 days, returning asymptomatic, middle ear effusion persists despite therapy at 9, 12, & 15 week follow up visits/15-month-old boy, 3 episodes acute otitis media in previous 3 months, asymptomatic, translucent, mobile tympanic membranes bilaterally | Scenario 1 (all (6, 9 & 12 weeks) visits combined): 152 treatment courses specified: 22% cefaclor, 22% amoxicillin  12 week visit: 121 would refer for tubes at/before now  2 (N=256, incl. 114 paediatricians): 239 responses: 54% antibiotic prophylaxis  10% chose referral for audiologic evaluation |
| Rudestam, 1981, Canada [164] | Questionnaire survey  Assess psychotropic drugs prescribing decisions by creating mathematical models of scenario-based decisions | 31 recruited, 24 analysed  19 M, 5 F  9 had 1-5 years’ experience, 5 6-10, 1 11-15, 5 16-20, 4 20+ | 63  7 symptom classes (% cases in which each occured): agitation, mania (25%); insomnia, fatigue (51%); irritable, anxious, fidgety (51%); low mood, hopeless, helpless (25%); psychosomatic problem (51%); psychotic thought disorder, paranoid, hostile suspicious (38%); withdrawn (38%)  Also varied age & gender | Across all scenarios: No. selecting: major tranquilizer: 419; minor tranquilizer: 463; antidepressant: 506; other 124  Second drug choice: modal response was none |
| Ryynanen, 1997, Finland [165] | Questionnaire survey  Investigate relationship between willingness to refer elderly patients with different common medical conditions for surgical operations & patient characteristics | No. recruited & analysed NS  No age/sex/experience data | 88; each received 18  3 represented hip arthrosis, 5 arterial obstruction in lower extremities, 6 coronary artery disease, 2 re-operation for coronary artery disease, 2 cataract  Age randomly varied, also varied comorbidity, living habits & dementia symptoms | N=837 (incl. other specialties):  % referring for surgery at age 65 ranged across scenarios from 17-100%  % referring for surgery at age 85 ranged across scenarios from 0-65% |
| Ryynanen, 2001, Finland [166] | Questionnaire survey  Investigate plain lumbar x-ray requests & attitudes to indications for lumbar spine radiography in relation to patient age & symptom duration | 615 recruited & analysed  46% M  Median year graduated from medical school 1982, range 1958-1995 | 45; each received 5  Varied age & symptom duration  All had low back pain | Decided whether to request a plain lumbar radiograph: results presented in graphs  For most cases, more requests when symptoms lasted 8 weeks  For some cases, more requests for older patients than younger ones when symptoms lasted 1/2 weeks |
| Sandvik, 1995, Norway [167] | Questionnaire survey  Compare responses to scenarios with actual performance on matched patients | 37 recruited, 32 analysed  6 F  Mean 16 years’ experience (range 4-43) | No. scenarios unclear: 27 were possible  Women with urinary incontinence  Included age, incontinence type, severity | 30 chose gynaecological examination; 30 urinalysis; 21 instruction in pelvic floor exercises  No. prescribing drugs in different categories: oestrogens=15; anticholinergics=6; sympathomimetics=4; others=2 |
| Schwartz, 1997, USA [168] | Questionnaire survey  Determine how acute purulent rhinitis is managed in primary care | 145 recruited & analysed  26% 0-5 years’ experience, 20% 6-10, 54% >10 | 2  Mother of 10-month-old calls, child fussy, cold for 2 days, clear/cloudy nasal discharge turned greenish yellow, in office later same day, tympanic membrane temp 38.2°C, eardrums reddened but not bulging/6 -year-old child with same history | Diagnostic decision results presented in graph  Scenario 1: 101 would immediately prescribe antibiotics  2: Participants more likely to opt against immediate antibiotic treatment, but 71 would still prescribe antibiotics |
| Shen, 2004, USA [169] | Questionnaire survey  Determine link between payment arrangement, clinical decision-making & physician distress | 601 recruited & analysed  19% F  Mean 17 years’ experience | 8; each received 4  Varied insurance status  50-year-old M, arthritis, takes over-counter medication, requests more costly prescription drug/healthy 28-year-old F, early pregnancy, requests foetal ultrasound/5-year-old boy, dog bite cheek, mother requests plastic surgery/61-year-old M, congestive heart failure, heart transplant only remaining therapy | % providing relevant treatment/management:  Fee for service & capitation scenarios:  Scenario 1: 76% & 55%  2: 47% & 33%  3: 78% & 67%  4: 92% & 92% |
| Smith, 2006, UK [170] | Questionnaire survey  Compare relative fits of ‘fast & frugal’ & linear regression models of prescription decisions for patients with/without depression symptoms | 62 recruited, 57 analysed  60% M, 40% F  Years’ experience range 8-47, mean 23 years | 20  Same as Smith et al. (2003) | Overall, 66% of decisions were to not prescribe antidepressant medication |
| Sorum, 2002a & 2002b, USA & France [171, 172] | Questionnaire survey  Investigate differences between parents & physicians in treatment decisions for children with possible acute otitis media | 54 recruited & analysed  No age/sex/experience data | 46  Children aged 15 months  Varied randomly on 15 cues: some clinical factors (e.g. asymmetry of tympanic membrane), some non-clinical (e.g. parents’ position concerning antibiotics) | N=75 (incl. 19 USA GPs, 35 French GPs, 21 French paediatricians):  Probability of acute otitis media:  USA: mean 50, range 28 to 75  France: mean 53, range 33 to 85  Probability of prescribing antibiotics or observing the child & degree of certainty in choice (5-point scales: scores combined to create score on 10-point scale (-5 (observe/completely sure) to 5 (treat/completely sure)):  USA: mean 0.22, range -2.4 to 3.0  France: mean 0.36, range -1.5 to 3.0  % cases judged as needing antibiotics:  USA: 53.0% France: 53.4% |
| Sorum, 2003, USA & France [173] | Postal questionnaire survey:  Understand why many prostate-specific antigen tests routinely ordered for asymptomatic male patients despite evidence-based recommendations | 65 recruited & analysed  USA: 17 Internists, 14 family physicians, 1 unknown  23 M, 8 F, 1 unknown  Mean 14 years’ experience (range 1–49)  France: 33 GPs  27 M, 4 F, 2 unknown  Mean 19 years of practice (range 10–26) | 32  Varied age, obstruction of urination symptoms, size of prostate on rectal exam, prostate shape, patient request or not for testing  All patients white, healthy, normal urinalyses, no prostate cancer family history | Decisions assessed on 11-point scales: 0 very unlikely-100% very likely:  Internists & family physicians & French GPs:  Mean probability of prostate cancer: 25% & 28% & 27%  Mean likelihood of ordering tests: 74% & 61% & 50% |
| Spiegel, 2009, USA [174] | Questionnaire survey  Compare adherence with dyspepsia best practice guidelines & identify specific areas of wide variation & influences on low adherence | 96 recruited & analysed  68% M  Mean 21 years’ experience | 3  44-year-old M, 12 months epigastric discomfort unrelated to NSAID use, no alarming features/47-year-old F, 12 months NSAID-related epigastric pain, no alarming features/ 58-year-old F, 6 months epigastric discomfort, nausea & bloating unrelated to NSAID use, no alarming features | Made diagnostic testing, treatment & follow-up decisions: no basic decisional data |
| Stoppe, 1999, Germany [175] | Interview survey  Investigate willingness to elicit a history of suicidal ideation in elderly depressed patients | 170 recruited & analysed  131 M, 39 F  Running practices for mean 11 years | 6; each received 2  Scenario 1: mild depression, free of other diseases, M/F  2: moderate-severe depression, M/F & stroke/thyroid disorder | Depression considered for primary or differential diagnosis by 91% for Case 1 & 70% for Case 2 |
| Swarztrauber, 2002, USA [176] | Questionnaire survey  Identify preferences regarding role of specialist in care of patients with common neurologic conditions & influence of nonclinical factors on preferences | 688 recruited & analysed  Internists & family physicians:  26% & 17% F  Mean 9 & 10 years’ experience | 3  Recent transient neurologic event & carotid stenosis/ Parkinsons disease patient on carbidopa-levodopa with dyskinesias/Patient with dementia symptoms | Decided whether to manage patient without specialty assistance/request a curbside consultation from specialist/formally refer patient to specialist:  Scenario 1: 48% internists (N=362) & 39% family physicians (N=304) chose to manage alone; 15% & 15% chose curbside; 37% & 46% referred  2. 37% internists (N=363) & 38% family physicians (N=303) chose to manage alone; 18% & 23% curbside; 47% & 39% referred  3. 74% internists (N=362) & 59% family physicians (N=304) chose to manage alone; 6% & 12% curbside; 20% & 29% referred |
| Teelucksingh, 2002, Trinidad & Tobago [177] | Questionnaire survey  Evaluate investigative & therapeutic approaches to Graves Disease, to allow comparison with practices elsewhere & inform national guidelines | 107 recruited & analysed  No age/sex/experience data | 5  F aged 43, moderate severity, 40-50 gram goitre, first episode/F aged 43, moderate severity, 40-50 gram goitre, 3-4 year history antithyroid drugs/M aged 43, moderate severity, 40-50 gram goitre, first episode/F aged 71, moderate severity, 40-50 gram goitre, first episode/F aged 16, severe, 80 gram goitre, 3-4 year history antithyroid drugs | N=130 (incl. 23 surgeons):  Scenario 1: decided what scanning procedures to perform to inform diagnosis & biochemical tests: 38% chose no scanning procedure, 48% chose thyroid function tests  Treatment:  1: 97 chose antithyroid drugs  2: 81 radioactive iodine  3: 95 antithyroid drugs  4: 74 antithyroid drugs  5: 79 surgery |
| Tracy, 2003, Canada [178] | Questionnaire survey  Examine relationships among attitudes toward evidence based medicine, contextual factors & clinical decision-making & investigate influences on 'contrary to evidence' clinical decisions | 431 recruited & analysed  Mean 16 years’ experience | 12; each received 4  Varied patient expectation: Wonders about or requests or demands:  Mammography/lumbar spine x-ray/antibiotics for bronchitis/ information not provided | Scenario 1: 37% (n = 152) would offer screening mammography  2. 23% (n = 99) order lumbar spine x-ray  3. 25% (n = 104) prescribe antibiotics  4. 80% (n = 341) teach breast self-examination |
| Vayda, 1981, Canada [179] | Questionnaire survey, pilot study  Identify differences in indication for certain operations & influences on differences | 40 recruited & analysed  No age/sex/experience data | 24  Four cases for each of six procedures: cholecystectomy/ inguinal herniorrhaphy/ hysterectomy/ caesarean section/ tonsillectomy/ colectomy | Decided whether to operate themselves/ refer to specialist for opinion/ refer to specialist for opinion & surgery/no referral  Control cases: agreement expected  Test cases: disagreement expected  Cholecystectomy:  Scenario 1=control: 21 chose refer for opinion  2=control: 30 refer for opinion & surgery  3=test: 26 no referral  4=test: 13 refer for opinion, 13 no referral  Hysterectomy cases (N=29):  5=control: 14 refer for opinion  6=test: 17 refer for opinion  7=test: 19 refer for opinion  8=test: 12 no referral |
| Vayda, 1982, Canada [180] | Questionnaire survey  Measure agreement in decision to refer/operate, & determine whether decisions made more frequently in counties with high operative rates | No. recruited & analysed NS  No age/sex/experience data | 23  3 cases for caesarean section, 4 for each of cholecystectomy/ inguinal herniorrhaphy/ hysterectomy/ colectomy/ tonsillectomy-adenoidectomy  All except caesarean section sent to 257 FPs: tonsillectomy-adenoidectomy cases also sent to another 40 FPs | N= 417 (incl. other specialties):  Control cases: agreement expected  Test cases: disagreement expected  % referring:  Cholecystectomy (N=126): 2 control cases: 93% & 44%; 2 test cases: 67% & 75%  Colectomy (N=124): 4 test cases: 69% & 74% & 61% & 71%  Inguinal herniorrhaphy (N=126): 1 control case: 86%; 3 test cases: 88% & 52% & 79%  Tonsillectomy-adenoidectomy (N=152): 2 control cases: 97% & 46%; 2 test cases: 75% & 57% |
| von dem Knesebeck, 2010, USA & UK & Germany [181] | Factorial experiment within interview survey  Examine differences in diabetes diagnosis & management in 3 countries, & assess influences on decisions | 192 analysed  No age/sex/experience data | 48; each received 2 (UK: 16 versions of 2 scenarios; USA: 24 versions of 2 scenarios; Germany: 8 versions of 2 scenarios)  Varied age, gender, SES, race (race not varied in Germany)  Diabetes signs & symptoms/diagnosed diabetes: burning in feet sometimes up to ankle, burning not constant | Analyses restricted to white patients for comparison purposes, N=64 each country:  Germany & UK & USA:  Scenario 1:  61% & 86% & 48% mentioned diabetes diagnosis  5% & 2% & 20% prescribed  8% & 22% & 11% chose any referral  Mean 3.7 & 10.9 & 15.7 days to next appointment  Mean 1.2 & 1.9 & 2.6 pieces of advice given  Scenario 2:  0% & 0% & 28% prescribed antidiabetic agents  2% & 27% & 27% chose podiatrist/chiropodist referral  Mean 20.1 7 34.2 & 38.0 days to next appointment  Mean 2.3 & 2.8 & 3.4 pieces of advice given |
| Votron, 2004, Belgium [182] | Questionnaire survey  Assess opinions on staging & treatment of prostate & breast cancer | 546 recruited & analysed  75% M  Median age 48 years | 14; each received 2  Varied age, medical history, health status  Prostate cancer & breast cancer scenarios | Decisions made concerning cancer diagnosis & staging & treatment: no basic decisional data |
| Weber, 1993, UK [183] | Questionnaire survey  Determine influence of case information, base rates, & expertise in generation of initial diagnostic hypotheses; identify determinants of size & diversity of initial hypothesis set; identify what stopping criterion terminates initial generation of hypotheses | No. recruited & analysed NS  Years’ experience range, 1-40 years | 48; each received 3  Varied amount of clinical & background information indicative of two plausible diagnoses (A/B; A=higher population base rate but less severe clinical consequences) | N=84 (incl. trainees):  Differential diagnosis: calculated rank of first mention of hypothesis; smaller ranks indicating earlier listing & thus greater strength  Mean ranks of A & B & other diagnoses (O) generated for each case:  Scenario 1: 1.2 & 3.1 & 3.8  2: 1.6 & 2.4 & 3.5  3: 1.1 & 3.0 & 3.3  Calculated frequency with which different versions of particular type of hypothesis listed, as second indicator of strength  Mean frequencies of A, B, & O diagnoses generated for each case:  1: 2.0 & 0.7 & 1.3  2: 1.2 & 1.3 & 1.5  3: 2.5 & 0.5 & 0.5 |
| Wilson, 2001, Canada & USA & UK [184] | Questionnaire survey  Determine influences on referral of patients with end stage renal disease to a nephrologist for dialysis & whether factors differed in three countries | 2,155 recruited & analysed  1282 M, 873 F  Canada & USA & UK: median year of graduation from medical school: 1982 & 1979 & 1979 | 10; each received 5  55-year-old patient with hypertension & different comorbid conditions, developed renal failure  Varied gender & severity of medical/ psychological/social condition | For all countries, as level of severity of condition/situation increased, % referring to a nephrologist for consideration for dialysis decreased, but downward trend different for each condition |
| Young, 1987, USA [185] | Questionnaire survey  Use descriptive thresholds to understand utilization of coronary arteriography | 119 recruited & analysed  7% F | 4  Patients with chest pain, similar in age, gender & SES, differed in pattern of chest pain & results of exercise testing | % recommending coronary arteriography per scenario presented in graph:  Scenario 1: between 90-100%, 2: 90-100%, 3: 40-50%, 4: 70-80% |

**Notes:** *ACE=angiotensin converting enzyme; CHD= coronary heart disease; COPD=chronic obstructive pulmonary disease; F=female; GP= General Practitioner (used to represent any variant in terminology used e.g. primary care physician /family physician etc.); HRT=hormone replacement therapy; M=male; NS=not specified; NSAID=nonsteroidal anti-inflammatory drug; SES=socioeconomic status; µg=microgram; URTI=upper respiratory tract infection; UTI=urinary tract infection*

*^a^Unless otherwise specified, participants responded to all scenarios;* **^b^***written scenarios unless otherwise specified;* ^c^*where ‘no basic decisional data’ is stated, studies did not report any descriptive statistics in relation to decisions made in response to scenarios*

**References**

1. Abdulmajeed A, Akram K, Tareq H, Gamal S, Hajar H, El-Zean A: **Outcome of a training course in psychiatry for primary health care physicians in Abu Dhabi, UAE.** *Primary Care Psychiatry* 2000, **6**(2):77-81.

2. Ang DC, Thomas K, Kroenke K: **An exploratory study of primary care physician decision making regarding total joint arthroplasty.** *J Gen Intern Med* 2007, **22**(1):74-79.

3. Arber S, McKinlay J, Adams A, Marceau L, Link C, O'Donnell A: **Patient characteristics and inequalities in doctors' diagnostic and management strategies relating to CHD: A video-simulation experiment.** *Soc Sci Med* 2006, **62**(1):103-115.

4. Arber S, McKinlay J, Adams A, Marceau L, Link C, O'Donnell A: **Influence of patient characteristics on doctors' questioning and lifestyle advice for coronary heart disease: A UK/US video experiment.** *Brit J Gen Pract* 2004, **54**(506):673-678.

5. Bonte M, von dem Knesebeck O, Siegrist J, Marceau L, Link C, Arber S, Adams A, McKinlay JB: **Women and men with coronary heart disease in three countries: are they treated differently?** *Womens Health Issues* 2008, **18**(3):191-198.

6. von dem Knesebeck O, Bonte M, Siegrist J, Marceau L, Link C, Arber S, Adams A, McKinlay J: **Country differences in the diagnosis and management of coronary heart disease - a comparison between the US, the UK and Germany.** *BMC Health Serv Res* 2008, **8**:198.

7. Lutfey KE, Link CL, Marceau LD, Grant RW, Adams A, Arber S, Siegrist J, Bonte M, von dem Knesebeck O, McKinlay JB: **Diagnostic certainty as a source of medical practice variation in coronary heart disease: results from a cross-national experiment of clinical decision making.** *Med Decis Making* 2009, **29**(5):606-618.

8. Lutfey KE, Link CL, Grant RW, Marceau LD, Mckinlay JB: **Is certainty more important than diagnosis for understanding race and gender disparities?: An experiment using coronary heart disease and depression case vignettes.** *Health Policy* 2009, **89**(3):279-287.

9. McKinlay J, Link C, Arber S, Marceau L, O'Donnell A, Adams A: **How do doctors in different countries manage the same patient? Results of a factorial experiment.** *Health Serv Res* 2006, **41**(6):2182-2200.

10. McKinlay JB, Link CL, Freund KM, Marceau LD, O'Donnell AB, Luffey KL: **Sources of variation in physician adherence with clinical guidelines: Results from a factorial experiment.** *J Gen Intern Med* 2007, **22**(3):289-296.

11. Binard A, Letebvre B, De Bandt M, Berthelot J, Saraux A: **Validity of the polymyalgia rheumatica activity score in primary care practice.** *Ann Rheum Dis* 2009, **68**(4):541-545.

12. Chin MH, Friedmann PD, Cassel CK, Lang RM: **Differences in generalist and specialist physicians' knowledge and use of angiotensin-converting enzyme inhibitors for congestive heart failure.** *J Gen Intern Med* 1997, **12**(9):523-530.

13. De Klippel N, Jansen J, Carlos JS: **Survey to evaluate diagnosis and management of headache in primary care: Headache Management Pattern programme.** *Curr Med Res Opin* 2008, **24**(12):3413-3422.

14. Di Caccavo A, Fazal-Short N, Moss T: **Primary care decision making in response to psychological complaints: The influence of patient race.** *J Community Appl Soc* 2000, **10**(1):63-67.

15. Emery J, Walton R, Murphy M, Austoker J, Yudkin P, Chapman C: **Computer support for interpreting family histories of breast and ovarian cancer in primary care: comparative study with simulated cases.** *BMJ* 2000, **321**(7252):28-32.

16. Glasspool D, Fox J, Coulson A, Emery J: **Risk assessment in genetics: A semi-quantitative approach.** *Medinfo 2001: Proceedings of the 10th World Congress on Medical Informatics, Pts 1 and 2* 2001, **84**:459-463.

17. Evink B, Crouse BJ, Elliott BA: **Diagnosing childhood attention-deficit/hyperactivity disorder. Do family practitioners and pediatricians make the same call?** *Minn Med* 2000, **83**(6):57-62.

18. Ferreira MB: **Different forms of using the same therapeutic tools in rhinitis: A comparison between general practitioners and allergologists using the OPERA questionnaire.** *Revista Portuguesa de Imunoalergologia* 2010, **18**(5):431-449.

19. Frayne S, Freund K, Skinner K, Ash A, Moskowitz M: **Depression management in medical clinics: Does healthcare sector make a difference?** *Am J Med Qual* 2004, **19**(1):28-36.

20. Frayne S, Skinner K, Lin H, Ash A, Freund K: **Effect of patient gender on late-life depression management.** *J Womens Health* 2004, **13**(8):919-925.

21. McKinlay JB, Lin T, Freund K, Moskowitz M: **The unexpected influence of physician attributes on clinical decisions: results of an experiment.** *J Health Soc Behav* 2002, **43**(1):92-106.

22. Freund KM, Moskowitz MA, Lin TH, McKinlay JB: **Early antidepressant therapy for elderly patients.** *Am J Med* 2003, **114**(1):15-19.

23. Glazier RH, Dalby DM, Badley EM, Hawker GA, Bell MJ, Buchbinder R, Lineker SC: **Management of the early and late presentations of rheumatoid arthritis: A survey of Ontario primary care physicians.** *CMAJ* 1996, **155**(6):679-687.

24. Glazier RH, Dalby DM, Badley EM, Hawker GA, Bell MJ, Buchbinder R, Lineker SC: **Management of common musculoskeletal problems: A survey of Ontario primary care physicians.** *CMAJ* 1998, **158**(8):1037-1040.

25. Hillson SD, Connelly DP, Liu Y: **The effects of computer-assisted electrocardiographic interpretation on physicians' diagnostic decisions.** *Med Decis Makin*g 1995, **15**(2):107-112.

26. Jiwa M, Gordon M, Arnet H, Ee H, Bulsara M, Colwell B: **Referring patients to specialists: a structured vignette survey of Australian and British GPs.** *BMC Fam Pract* 2008, **9**:2.

27. Jones K, Gruffydd-Jones K: **Management of acute asthma attacks associated with respiratory tract infection: A postal survey of general practitioners in the U.K.** *Respir Med* 1996, **90**(7):419-425.

28. Kales H, Neighbors H, Valenstein M, Blow F, McCarthy J, Ignacio R, Taylor K, Gillon L, Mellow A: **Effect of race and sex on primary care physicians' diagnosis and treatment of late-life depression.** *J Am Geriatr Soc* 2005, **53**(5):777-784.

29. Kuyvenhoven MM, Jacobs HM, Touw-Otten FWMM, Van Es JC: **Written simulation of patient-doctor encounters. 1. Research instrument for registration of the performance of general practitioners.** *Fam Pract* 1984, **1**(1):14-19.

30. Murray S, Del Mar C, O'Rourke P: **Predictors of an antibiotic prescription by GPs for respiratory tract infections: A pilot.** *Fam Pract* 2000, **17**(5):386-388.

31. Park SY, Gerber MA, Tanz RR, Hickner JM, Galliher JM, Chuang I, Besser RE: **Clinicians' management of children and adolescents with acute pharyngitis.** *Pediatrics* 2006, **117**(6):1871-1878.

32. Persell SD, Zei C, Cameron KA, Zielinski M, Lloyd-Jones DM: **Potential use of 10-year and lifetime coronary risk information for preventive cardiology prescribing decisions: A primary care physician survey.** *Arch Intern Med* 2010, **170**(5):470-477.

33. Rose PW, Watson E, Yudkin P, Emery J, Murphy M, Fuller A, Lucassen A: **Referral of patients with a family history of breast/ovarian cancer - GPs' knowledge and expectations.** *Fam Pract* 2001, **18**(5):487-490.

34. Schulman KA, Berlin JA, Harless W, Kerner JF, Sistrunk S, Gersh BJ, Dube R, Taleghani CK, Burke JE, Williams S, Eisenberg JM, Escarce JJ, Ayers W: **The effect of race and sex on physicians' recommendations for cardiac catheterization.** *N Engl J Med* 1999, **340**(8):618-626.

35. Shackelton R, Link C, Marceau L, McKinlay J: **Does the culture of a medical practice affect the clinical management of diabetes by primary care providers?** *Journal of Health Services Research and Policy* 2009, **14**(2):96-103.

36. Shackelton RJ, Marceau LD, Link CL, McKinlay JB: **The intended and unintended consequences of clinical guidelines.** *J Eval Clin Pract* 2009, **15**(6):1035-1042.

37. Shackelton-Piccolo R, McKinlay JB, Marceau LD, Goroll AH, Link CL: **Differences between internists and family practitioners in the diagnosis and management of the same patient with coronary heart disease.** *Med Care Res Rev* 2011, **68**(6):650-666.

38. Sohn W, Ismail AI, Taichman LS: **Caries risk-based fluoride supplementation for children.** *Pediatr Dent* 2007, **29**(1):23-31.

39. Spiegel BMR, Farid M, Esrailian E, Talley J, Chang L: **Is irritable bowel syndrome a diagnosis of exclusion?: A survey of primary care providers, gastroenterologists, and ibs experts.** *Am J Gastroenterol* 2010, **105**(4):848-858.

40. Stoppe G, Sandholzer H, Staedt J, Winter S, Kiefer J, Kochen MM, Ruther E: **Diagnosis of dementia in primary care: Results of a representative survey in Lower Saxony, Germany.** *Eur Arch Psychiatry Clin Neurosci* 1994, **244**(5):278-283.

41. Stoppe G, Sandholzer H, Staedt J, Winter S, Kiefer J, Ruther E: **Prescribing practice with cognition enhancers in outpatient care: are there differences regarding type of dementia?--Results of a representative survey in lower Saxony, Germany.** *Pharmacopsychiatry* 1996, **29**(4):150-155.

42. Stoppe G, Sandholzer H, Staedt J, Winter S, Kiefer J, Ruther E: **Sleep disturbances in the demented elderly: Treatment in ambulatory care.** *Sleep* 1995, **18**(10):844-848.

43. Stoppe G, Sandholzer H, Staedt J, Winter S, Kiefer J, Ruther E: **Reasons for prescribing cognition enhancers in primary-care - results of a representative survey in Lower Saxony, Germany.** *Int J Clin Pharmacol Ther* 1995, **33**(9):486-490.

44. Stoppe G, Haak S, Knoblauch A, Maeck L: **Diagnosis of dementia in primary care: A representative survey of family physicians and neuropsychiatrists in Germany.** *Dement Geriatr Cogn Disord* 2007, **23**(4):207-214.

45. Tiemeier H, De Vries WJ, Van het Loo M, Kahan JP, Klazinga N, Grol R, Rigter H: **Guideline adherence rates and interprofessional variation in a vignette study of depression.** *Qual Saf Health Care* 2002, **11**(3):214-218.

46. Tucker J, Farmer J, Stimpson P: **Guidelines and management of mild hypertensive conditions in pregnancy in rural general practices in Scotland: Issues of appropriateness and access.** *Qual Saf Health Care* 2003, **12**(4):286-290.

47. Vancheri F, Strender L, Bring J, Montgomery H, Skaner Y, Backlund LG: **General practitioners' coronary risk assessments and lipid-lowering treatment decisions in primary prevention: Comparison between two European areas with different cardiovascular risk levels.** *Primary Health Care Research and Development* 2008, **9**(4):248-256.

48. Vancheri F, Strender L, Montgomery H, Skaner Y, Backlund LG: **Coronary risk estimates and decisions on lipid-lowering treatment in primary prevention. Comparison between general practitioners, internists, and cardiologists.** *Eur J Intern Med* 2009, **20**(6):601-606.

49. Watson E, Clements A, Yudkin P, Rose P, Bukach C, Mackay J, Lucassen A, Austoker J: **Evaluation of the impact of two educational interventions on GP management of familial breast/ovarian cancer cases: A cluster randomised controlled trial.** *Brit J Gen Pract* 2001, **51**(471):817-821.

50. Webster BS, Courtney TK, Huang YH, Matz S, Christiani DC: **Physicians' initial management of acute low back pain versus evidence-based guidelines. Influence of sciatica.** *J Gen Intern Med* 2005, **20**(12):1132-1135.

51. Webster BS, Courtney TK, Huang Y-, Matz S, Christiani DC: **Survey of acute low back pain management by specialty group and practice experience.** *J Occup Environ Med* 2006, **48**(7):723-732.

52. White DB, Bonham VL, Jenkins J, Stevens N, McBride CM: **Too many referrals of low-risk women for BRCA1/2 genetic services by family physicians.** *Cancer Epidem Biomar* 2008, **17**(11):2980-2986.

53. Wijeratne C, Harris P: **Late life depression and dementia: a mental health literacy survey of Australian general practitioners.** *Int Psychogeriatr* 2009, **21**(2):330-337.

54. Windak A, Gryglewska B, Tomasik T, Narkiewicz K, Grodzicki T: **Competence of general practitioners in giving advice about changes in lifestyle to hypertensive patients.** *Med Decis Making* 2009, **29**(2):217-223.

55. Windak A, Gryglewska B, Tomasik T, Narkiewicz K, Grodzicki T: **General practitioners ignore high normal blood pressure.** *Blood Press* 2008, **17**(1):42-49.

56. Windak A, Gryglewska B, Tomasik T, Narkiewicz K, John Y, Grodzicki T: **Competence of Polish primary-care doctors in the pharmacological treatment of hypertension.** *J Eval Clin Pract* 2010, **16**(1):25-30.

57. Windak A, Gryglewska B, Tomasik T, Narkiewicz K, Yaphe J, Grodzicki T: **The competence of primary care doctors in the investigation of patients with elevated blood pressure: results of a cross-sectional study using clinical vignettes RID B-8034-2010.** *J Eval Clin Pract* 2010, **16**(4):784-789.

58. Yanovski SZ, Yanovski JA, Malley JD, Brown RL, Balaban DJ: **Telephone triage by primary care physicians.** *Pediatrics* 1992, **89**(4 SUPPL):701-706.

59. Backlund L, Danielsson B, Bring J, Strender L-: **Factors influencing GPs' decisions on the treatment of hypercholesterolaemic patients.** *Scand J Prim Health Care* 2000, **18**(2):87-93.

60. Bonetti D, Eccles M, Johnston M, Steen N, Grimshaw J, Baker R, Walker A, Pitts N: **Guiding the design and selection of interventions to influence the implementation of evidence-based practice: an experimental simulation of a complex intervention trial RID C-5245-2008.** *Soc Sci Med* 2005, **60**(9):2135-2147.

61. Carroll JC, Wilson BJ, Allanson J, Grimshaw J, Blaine SM, Meschino WS, Permaul JA, Graham ID: **GenetiKit: A randomized controlled trial to enhance delivery of genetics services by family physicians.** *Fam Pract* 2011, **28**(6) (pp 615-623).

62. Chavannes N, Schermer T, Akkermans R, Jacobs JE, van de Graaf G, Bollen R, van Schayck O, Bottema B: **Impact of spirometry on GPs' diagnostic differentiation and decision-making.** *Respir Med* 2004, **98**(11):1124-1130.

63. Cherkin DC, Deyo RA, Wheeler K, Ciol MA: **Physician variation in diagnostic testing for low back pain: Who you see is what you get.** *Arthritis Rheum* 1994, **37**(1):15-22.

64. Mosca L, Linfante AH, Benjamin EJ, Berra K, Hayes SN, Walsh BW, Fabunmi RP, Kwan J, Mills T, Simpson SL: **National study of physician awareness and adherence to cardiovascular disease prevention guidelines.** *Circulation* 2005, **111**(4):499-510.

65. Christian AH, Mills T, Simpson SL, Mosca L: **Quality of cardiovascular disease preventive care and physician/practice characteristics.** *J Gen Intern Med* 2006, **21**(3):231-237.

66. De Melker RA, Kuyvenhoven MM: **Management of upper respiratory tract infection in Dutch general practice.** *Brit J Gen Pract* 1991, **41**(353):504-507.

67. Elinson L, Cohen MM, Elmslie T: **Hormone replacement therapy: A survey of Ontario physicians' prescribing practices.** *CMAJ* 1999, **161**(6):695-698.

68. Everitt DE, Avorn J, Baker MW: **Clinical decision-making in the evaluation and treatment of insomnia.** *Am J Med* 1990, **89**(3):357-362.

69. Mahoney DF: **Appropriateness of geriatric prescribing decisions made by nurse practitioners and physicians.** *Image J Nurs Sch* 1994, **26**(1):41-46.

70. Ferris DG, Miller MD, Wagner P, Walaitis E, Lawler FH: **Clinical decision-making following abnormal Papanicolaou smear reports.** *Fam Pract Res J* 1993, **13**(4):343-353.

71. Fortinsky RH, Leighton A, Wasson JH: **Primary care physicians' diagnostic, management, and referral practices for older persons and families affected by dementia.** *Res Aging* 1995, **17**(2):124-148.

72. Foster JA, Yawn BP, Maziar A, Jenkins T, Rennard SI, Casebeer L: **Enhancing COPD management in primary care settings.** *MedGenMed* 2007, **9**(3):24.

73. Hobus PPM, Schmidt HG, Boshuizen HPA, Patel VL: **Contextual factors in the activation of first diagnostic hypotheses: Expert-novice differences.** *Med Educ* 1987, **21**(6):471-476.

74. Hummers-Pradier E, Denig P, Oke T, Lagerlov P, Wahlstrom R, Haaijer-Ruskamp F, DEP Grp: **GPs' treatment of uncomplicated urinary tract infections - a clinical judgement analysis in four European countries.** *Fam Pract* 1999, **16**(6):605-607.

75. James PA, Cowan TM, Graham RP: **Patient-centered clinical decisions and their impact on physician adherence to clinical guidelines.** *J Fam Pract* 1998, **46**(4):311-318.

76. Kostopoulou O, Oudhoff J, Nath R, Delaney BC, Munro CW, Harries C, Holder R: **Predictors of diagnostic accuracy and safe management in difficult diagnostic problems in family medicine.** *Med Decis Making* 2008, **28**(5):668-680.

77. Marwill SL, Freund KM, Barry PP: **Patient factors associated with breast cancer screening among older women.** *J Am Geriatr Soc* 1996, **44**(10):1210-1214.

78. Middleton S, Sharpe D, Harris J, Corbett A, Lusby R, Ward J: **Case Scenarios to Assess Australian General Practitioners' Understanding of Stroke Diagnosis, Management, and Prevention.** *Stroke* 2003, **34**(11):2681-2686.

79. Peabody JW, Tozija F, Munoz JA, Nordyke RJ, Luck J: **Using vignettes to compare the quality of clinical care variation in economically divergent countries.** *Health Serv Res* 2004, **39**(6):1951-1970.

80. Persson M, Carlberg B, Tavelin B, Lindholm LH: **Doctors' estimation of cardiovascular risk and willingness to give drug treatment in hypertension: Fair risk assessment but defensive treatment policy.** *J Hypertens* 2004, **22**(1):65-71.

81. Poels PJ, Schermer TR, Schellekens DP, Akkermans RP, de Vries RPF, Kaplan A, Bottema BJ, van Weel C: **Impact of a spirometry expert system on general practitioners' decision making.** *Eur Respir J* 2008, **31**(1):84-92.

82. Ribacke M: **Treatment preferences, return visit planning and factors affecting hypertension practice amongst general practitioners and internal medicine specialists (The general practitioner hypertension practice study).** *J Intern Med* 1995, **237**(5):473-478.

83. Ribacke M: **The concept of individualized hypertension care in general practice and outpatient clinics. The general practitioner hypertension practice study (III).** *Scand J Prim Health Care* 1995, **13**(2):112-117.

84. Short D, Frischer M, Bashford J: **The development and evaluation of a computerised decision support system for primary care based upon 'patient profile decision analysis'.** *Informatics in Primary Care* 2003, **11**(4):195-202.

85. Smith L, Gilhooly K, Walker A: **Factors influencing prescribing decisions in the treatment of depression: A social judgement theory approach.** *Appl Cog Psych* 2003, **17**(1):51-63.

86. Wagner KR, Elmore JG, Horwitz RI, Hines J, H.H: **Diagnostic bias in clinical decision making: An example of L-tryptophan and the diagnosis of eosinophilia-myalgia syndrome.** *J Rheumatol* 1996, **23**(12):2079-2085.

87. Wahlstrom R, Hummers-Pradier E, Lundborg C, Muskova M, Lagerlov P, Denig P, Oke T, de Saintonge D: **Variations in asthma treatment in five European countries - judgement analysis of case simulations.** *Fam Pract* 2002, **19**(5):452-460.

88. Walton RT, Gierl C, Yudkin P, Mistry H, Vessey MP, Fox J: **Evaluation of computer support for prescribing (CAPSULE) using simulated cases.** *BMJ* 1997, **315**(7111):791-795.

89. Wigton RS, Darr CA, Corbett KK, Nickol DR, Gonzales R: **How do community practitioners decide whether to prescribe antibiotics for acute respiratory tract infections?** *J Gen Intern Med* 2008, **23**(10):1615-1620.

90. Arnold S, To T, McIsaac W, Wang F: **Antibiotic prescribing for upper respiratory tract infection: The importance of diagnostic uncertainty.** *J Pediatr* 2005, **146**(2):222-226.

91. Bedson J, Jordan K, Croft P: **How do GPs use x rays to manage chronic knee pain in the elderly? A case study.** *Ann Rheum Dis* 2003, **62**(5):450-454.

92. BeLue R, Butler J, Kuder J: **Implications of patient and physician decision making: An illustration in treatment options for coronary artery disease.** *J Ambulatory Care Manage* 2004, **27**(4):305-313.

93. Berg AO: **Variations among family physicians' management strategies for lower urinary tract infection in women: a report from the Washington Family Physicians Collaborative Research Network.** *J Am Board Fam Pract* 1991, **4**(5):327-330.

94. Bernstein B, Kane R: **Physicians' attitudes toward female patients.** *Med Care* 1981, **19**(6):600-608.

95. Bierma-Zeinstra SMA, Lipschart S, Njoo KH, Bernsen R, Verhaar J, Prins A, Bohnen AM: **How do general practitioners manage hip problems in adults?** *Scand J Prim Health Care* 2000, **18**(3):159-164.

96. Bitzer J, Frey B, Von Schonau M, Sabler N, Tschudin S: **Twenty or thirty microgram ethinyloestradiol in an oral contraceptive: Does it make a difference in the mind and the daily practise of gynaecologists and general practitioners.** *Eur J Contracep Repr* 2009, **14**(4):258-267.

97. Borkhoff CM, Hawker GA, Kreder HJ, Glazier RH, Mahomed NN, Wright JG: **Patients' gender affected physicians' clinical decisions when presented with standardized patients but not for matching paper patients.** *J Clin Epidemiol* 2009, **62**(5):527-541.

98. Bremberg S, Nilstun T: **Justifications of physicians' choice of action - Attitudes among the general public, GPs, and oncologists in Sweden.** *Scand J Prim Health Care* 2005, **23**(2):102-108.

99. Bremberg S, Nilstun T, Kovac V, Zwitter M: **GPs facing reluctant and demanding patients: Analysing ethical justifications.** *Fam Pract* 2003, **20**(3):254-261.

100. Bryant-Waugh RJ, Lask BD, Shafran RL, Fosson AR: **Do doctors recognise eating disorders in children?** *Arch Dis Child* 1992, **67**(1):103-105.

101. Burgess DJ, Crowley-Matoka M, Phelan S, Dovidio JF, Kerns R, Roth C, Saha S, van Ryn M: **Patient race and physicians' decisions to prescribe opioids for chronic low back pain.** *Soc Sci Med* 2008, **67**(11):1852-1860.

102. Phelan SM, van Ryn M, Wall M, Burgess D: **Understanding primary care physicians' treatment of chronic low back pain: the role of physician and practice factors RID B-1664-2010.** *Pain Med* 2009, **10**(7):1270-1279.

103. Campbell A, Ogden J: **Why do doctors issue sick notes? An experimental questionnaire study in primary care.** *Fam Pract* 2006, **23**(6):125-130.

104. Carey T, Garrett J, Curtis P, Darter J, DeFriese G, Fryer J, Hadler N, Hunter G, Joines J, Jackman A, Kalsbeek W, McLaughlin C, Konrad T, Ricketts T, Taylor D, McNutt R, Smucker D: **Patterns of ordering diagnostic tests for patients with acute low back pain.** *Ann Intern Med* 1996, **125**(10):807-&.

105. Carr N, Wales S, Young D: **Reported management of patients with sore throat in Australian general-practice.** *Brit J Gen Pract* 1994, **44**(388):515-518.

106. Chapman K, Tashkin D, Pye D: **Gender bias in the diagnosis of COPD.** *Chest* 2001, **119**(6):1691-1695.

107. Chaput De Saintonge DM, Hathaway NR: **Antibiotic use in otitis media: Patient simulations as an aid to audit.** *BMJ* 1981, **283**(6296):883-884.

108. Chaput de Saintonge DM, Hattersley LA: **Antibiotics for otitis media: Can we help doctors agree?** *Fam Pract* 1985, **2**(4):205-212.

109. Cicutto L, Llewellyn-Thomas H, Geerts W: **The management of asthma: A case-scenario-based survey of family physicians and pulmonary specialists.** *J Asthma* 2000, **37**(3):235-246.

110. Currin L, Schmidt U, Waller G: **Variables that influence diagnosis and treatment of the eating disorders within primary care settings: A vignette study.** *Int J Eat Disord* 2007, **40**(3):257-262.

111. Currin L, Waller G, Schmidt U: **Primary care physicians' knowledge of and attitudes toward the eating disorders: Do they affect clinical actions?** *Int J Eat Disord* 2009, **42**(5):453-458.

112. Davidson RA, Meuleman JR: **Initial treatment of hypertension: A questionnaire survey.** *J Clin Hypertens* 1986, **2**(4):339-345.

113. De Bock GH, Reijneveld SA, Van Houwelingen JC, Knottnerus JA, Kievit J: **Multiattribute utility scores for predicting family physicians' decisions regarding sinusitis.** *Med Decis Making* 1999, **19**(1):58-65.

114. Diette GB, Patino CM, Merriman B, Paulin L, Riekert K, Okelo S, Thompson K, Krishnan JA, Quartey R, Perez-Williams D, Rand C: **Patient factors that physicians use to assign asthma treatment.** *Arch Intern Med* 2007, **167**(13):1360-1366.

115. Doust JA, Silagy CA: **Applying the results of a systematic review in general practice.** *Med J Aust* 2000, **172**(4):153-156.

116. Elson RB, Connelly DP: **The impact of anticipatory patient data displays on physician decision making: a pilot study.** *Proc AMIA Annu Fall Symp* 1997, :233-237.

117. Epstein SA, Hooper LM, Weinfurt KP, DePuy V, Cooper LA, Harless WG, Tracy CM: **Primary care physicians' evaluation and treatment of depression: Results of an experimental study using video vignettes.** *Med Care Res Re*v 2008, **65**(6):674-695.

118. Hooper LM, Weinfurt KP, Cooper LA, Mensh J, Harless W, Kuhajda MC, Epstein SA: **Virtual standardized patients: An interactive method to examine variation in depression care among primary care physicians.** *Primary Health Care Research and Development* 2008, **9**(4):257-268.

119. Eva KW, Link CL, Lutfey KE, McKinlay JB: **Swapping horses midstream: Factors related to physicians' changing their minds about a diagnosis.** *Acad Med* 2010, **85**(7):1112-1117.

120. Ketcham JD, Lutfey KE, Gerstenberger E, Link CL, McKinlay JB: **Physician clinical information technology and health care disparities.** *Med Care Res Rev* 2009, **66**(6):658-681.

121. Lutfey KE, Eva KW, Gerstenberger E, Link CL, McKinlay JB: **Physician cognitive processing as a source of diagnostic and treatment disparities in coronary heart disease: results of a factorial priming experiment.** *J Health Soc Behav* 2010, **51**(1):16-29.

122. Evans S, J.B.T, Harries C, Dennis I, Dean J: **General practitioners' tacit and stated policies in the prescription of lipid lowering agents.** *Brit J Gen Pract* 1995, **45**(390):15-18.

123. Harries C, Evans J, Dennis I, Dean J: **A clinical judgement analysis of prescribing decisions in general practice.** *Travail Humain* 1996, **59**(1):87-109.

124. Farmer A, Griffiths H: **Labeling and illness in primary care - comparing factors influencing general-practitioners and psychiatrists decisions regarding patient referral to mental-illness services.** *Psychol Med* 1992, **22**(3):717-723.

125. Finkelstein BS, Silvers JB, Marrero U, Neuhauser D, Cuttler L: **Insurance coverage, physician recommendations, and access to emerging treatments: Growth hormone therapy for childhood short stature.** *JAMA* 1998, **279**(9):663-668.

126. Friedmann P, Brett A, MayoSmith M: **Differences in generalists' and cardiologists' perceptions of cardiovascular risk and the outcomes of preventive therapy in cardiovascular disease.** *Ann Intern Med* 1996, **124**(4):414-&.

127. Gagnon F, Lareau-Trudel E, Bachand M-, Hamel C, Lapointe V, Dubois M-, Xhignesse M, Langlois M: **Management of thyroid dysfunction by primary care physicians of the Province of Quebec: A transversal study.** *Ann Endocrinol* 2008, **69**(3):210-217.

128. Gerbert B: **Perceived likeability and competence of simulated patients: influence on physicians' management plans.** *Soc Sci Med* 1984, **18**(12):1053-1059.

129. Grant RW, Lutfey KE, Gerstenberger E, Link CL, Marceau LD, McKinlay JB: **The decision to intensify therapy in patients with type 2 diabetes: Results from an experiment using a clinical case vignette.** *J Am Board Fam Med* 2009, **22**(5):513-520.

130. Gupta R, Stocks NP, Broadbent J: **Cardiovascular risk assessment in Australian general practice.** *Aust Fam Physician* 2009, **38**(5):364-368.

131. Haggerty J, Tudiver F, Brown JB, Herbert C, Ciampi A, Guibert R: **Patients' anxiety and expectations: how they influence family physicians' decisions to order cancer screening tests.** *Can Fam Physician* 2005, **51**:1658-1659.

132. Tudiver F, Guibert R, Haggerty J, Ciampi A, Medved W, Brown JB, Herbert C, Katz A, Ritvo P, Grant B, Goel V, Smith P, O'Beirne M, Williams JI, Moliner P: **What influences family physicians' cancer screening decisions when practice guidelines are unclear or conflicting?** *J Fam Pract* 2002, **51**(9):760.

133. Halpin DMG, O'Reilly JF, Connellan S, Rudolf M: **Confidence and understanding among general practitioners and practice nurses in the UK about diagnosis and management of COPD.** *Respir Med* 2007, **101**(11):2378-2385.

134. Halvorsen PA, Wennevold K, Fleten N, Muras M, Kowalczyk A, Godycki-Cwirko M, Melbye H: **Decisions on sick leave certifications for acute airways infections based on vignettes: a cross-sectional survey of GPs in Norway and Poland.** *Scand J Prim Health Care* 2011, **29**(2):110-116.

135. Hamilton-Craig CR, Tonkin AL, Jobling RG: **How accurate are hypertension treatment decisions? Absolute risk assessment and prescribing for moderate hypertension - A study of Cambridge general practitioners.** *Blood Press* 2000, **9**(6):323-327.

136. Harries C, Forrest D, Harvey N, McClelland A, Bowling A: **Which doctors are influenced by a patient's age? A multi-method study of angina treatment in general practice, cardiology and gerontology.** *Qual Saf Health Care* 2007, **16**(1):23-27.

137. Hart J, Salman H, Bergman M, Neuman V, Rudniki C, Gilenberg D, Matalon A, Djaldetti M: **Do drug costs affect physicians' prescription decisions?** *J Intern Med* 1997, **241**(5):415-420.

138. Hartley RM, Charlton JR, Jarman B, Harris CM: **Case history questionnaires in the study of doctors' use of resources. Are they measuring what we want?** *Med Care* 1985, **23**(10):1163-1170.

139. Horne R, Coombes I, Davies G, Hankins M, Vincent R: **Barriers to optimum management of heart failure by general practitioners RID C-6000-2009.** *Brit J Gen Pract* 1999, **49**(442):353-357.

140. Howie JG: **Further observations on diagnosis and management of general practice respiratory illness using simulated patient consultations.** *BMJ* 1974, **2**(5918):540-543.

141. Hrisos S, Eccles M, Johnston M, Francis J, Kaner EF, Steen N, Grimshaw J: **An intervention modelling experiment to change GPs' intentions to implement evidence-based practice: using theory-based interventions to promote GP management of upper respiratory tract infection without prescribing antibiotics #2.** *BMC Health Serv Res* 2008, **8**:10.

142. Jenkins R, Smeeton N, Shepherd M: **Classification of mental disorder in primary care.** *Psychol Med. Monograph Suppl* 1988, **12**:1-59.

143. Kaner E, Wutzke S, Saunders J, Powell A, Morawski J, Bouix J: **Impact of alcohol education and training on general practitioners' diagnostic and management skills: Findings from a World Health Organization collaborative study.** *J Stud Alcohol* 2001, **62**(5):621-627.

144. Kellen JC, Russell ML: **Physician specialty is associated with differences in warfarin use for atrial fibrillation.** *Can J Cardiol* 1998, **14**(3):365-368.

145. Kesten S, Chapman KR: **Physician perceptions and management of COPD.** *Chest* 1993, **104**(1):254-258.

146. Kikano GE, Stange KC, Flocke SA, Zyzanski SJ: **Effect of the white blood count on the clinical management of the febrile infant.** *J Fam Pract* 1991, **33**(5):465-469.

147. Kikano GE, Schiaffino MA, Zyzanski SJ: **Medical decision making and perceived socioeconomic class.** *Arch Fam Med* 1996, **5**(5):267-270.

148. Kuder JM, Vilmain JA, Demlo LK: **Exploring physician responses to patients' extramedical characteristics. The decision to hospitalize.** *Med Care* 1987, **25**(9):882-893.

149. Little DR, Mann BL, Sherk DW: **Factors influencing the clinical diagnosis of sinusitis.** *J Fam Pract* 1998, **46**(2):147-152.

150. Lynggaard MD, Strandgaard S: **Factors influencing the decision to start drug treatment in hypertension. A questionnaire study comparing general practitioners and hypertension specialists in Denmark.** *Blood Press* 2006, **15**(4):207-212.

151. Matthews K, Eagles JM, Matthews CA: **The use of antidepressant drugs in general practice. A questionnaire survey.** *Eur J Clin Pharmacol* 1993, **45**(3):205-210.

152. McCranie EW, Horowitz AJ, Martin RM: **Alleged sex-role stereotyping in the assessment of women's physical complaints: a study of general practitioners.** *Soc Sci Med* 1978, **12**(2A):111-116.

153. Modi SC, Whetstone LM, Cummings DM: **Influence of patient and physician characteristics on percutaneous endoscopic gastrostomy tube decision-making.** *J Palliat Med* 2007, **10**(2):359-366.

154. Montgomery AJ, McGee HM, Shannon W, Donohoe J: **Factors influencing general practitioner referral of patients developing end-stage renal failure: a standardised case-analysis study.** *BMC Healh Serv Res* 2006, **6**:114.

155. Montori VM, Leung TW, Devereaux PJ, Schunemann HJ, Akl EA, Gafni A, Guyatt GH: **Can contraindications compromise evidence-based, patient-centered clinical practice?** *Canadian Journal of Clinical Pharmacology* 2006, **13**(1):e92-e101.

156. Morrell DC, Roland MO: **Analysis of referral behaviour: responses to simulated case histories may not reflect real clinical behaviour.** *Brit J Gen Pract* 1990, **40**(334):182-185.

157. Neuner JM, Laud PW, Schapira MM: **A randomized study of the effect of 5-year and lifetime hip fracture risk information on physician recommendations for management of low bone density.** *J Clin Densitom* 2007, **10**(4):370-375.

158. Neuner JM, Schapira MM: **The Importance of Physicians' Risk Perception in Osteoporosis Treatment Decision Making.** *J Clin Densitom* 2012, **15**(1):49-54.

159. Nikolajevic-Sarunac J, Henry D, O'Connell D, Robertson C: **Effects of information framing on the intentions of family physicians to prescribe long-term hormone replacement therapy.** *J Gen Intern Med* 1999, **14**(10):591-598.

160. Petitti D, Grumbach K: **Variation in physicians recommendations about revisit interval for 3 common conditions.** *J Fam Pract* 1993, **37**(3):235-240.

161. Potter M, Schafer S, Gonzalez-Mendez E, Gjeltema K, Lopez A, Wu J, Pedrin R, Cozen M, Wilson R, Thom D, Croughan-Minihane M: **Opioids for chronic nonmalignant pain: Attitudes and practices of primary care physicians in the UCSF/Stanford Collaborative Research Network.** *J Fam Pract* 2001, **50**(2):145-151.

162. Rathore SS, Ketcham JD, Alexander GC, Epstein AJ: **Influence of patient race on physician prescribing decisions: A randomized on-line experiment.** *J Gen Intern Med* 2009, **24**(11):1183-1191.

163. Roark R, Petrofski J, Berson E, Berman S: **Practice variations among pediatricians and family physicians in the management of otitis media.** *Arch Pediatr Adolesc Med* 1995, **149**(8):839-844.

164. Rudestam KE, Tarbell SE: **The clinical judgement process in the prescribing of psychotropic drugs.** *Int J Addict* 1981, **16**(6):1049-1070.

165. Ryynanen OP, Myllykangas M, Kinnunen J, Takala J: **Doctors' willingness to refer elderly patients for elective surgery.** *Fam Pract* 1997, **14**(3):216-219.

166. Ryynanen OP, Lehtovirta J, Soimakallio S, Takala J: **General practitioners' willingness to request plain lumbar spine radiographic examinations.** *Eur J Radiol* 2001, **37**(1):47-53.

167. Sandvik H: **Criterion validity of responses to patient vignettes: An analysis based on management of female urinary incontinence.** *Fam Med* 1995, **27**(6):388-392.

168. Schwartz R, Freij B, Ziai M, Sheridan M: **Antimicrobial prescribing for acute purulent rhinitis in children: A survey of pediatricians and family practitioners.** *Pediatr Infect Dis J* 1997, **16**(2):185-190.

169. Shen J, Andersen R, Brook R, Kominski G, Albert PS, Wenger N: **The effects of payment method on clinical decision-making: physician responses to clinical scenarios.** *Med Care* 2004, **42**(3):297-302.

170. Smith L, Gilhooly K: **Regression versus fast and frugal models of decision-making: The case of prescribing for depression.** *Appl Cognitive Psych* 2006, **20**(2):265-274.

171. Sorum PC, Shim J, Chasseigne G, Mullet E, Sastre MTM, Stewart T, Gonzalez-Vallejo C: **Do parents and physicians differ in making decisions about acute otitis media?** *J Fam Pract* 2002, **51**(1):51-57.

172. Sorum P, Stewart T, Mullet E, Gonzalez-Vallejo C, Shim J, Chasseigne G, Sastre M, Grenier B: **Does choosing a treatment depend on making a diagnosis? US and French physicians' decision making about acute otitis media.** *Med Decis Making* 2002, **22**(5):394-402.

173. Sorum PC, Shim J, Chasseigne G, Bonnin-Scaon S, Cogneau J, Mullet E: **Why do primary care physicians in the United States and France order prostate-specific antigen tests for asymptomatic patients?** *Med Decis Making* 2003, **23**(4):301-313.

174. Spiegel BMR, Farid M, Van Oijen MGH, Laine L, Howden CW, Esrailian E: **Adherence to best practice guidelines in dyspepsia: A survey comparing dyspepsia experts, community gastroenterologists and primary-care providers.** *Aliment Pharm Ther* 2009, **29**(8):871-881.

175. Stoppe G, Sandholzer H, Huppertz C, Duwe H, Staedt J: **Family physicians and the risk of suicide in the depressed elderly.** *J Affect Disord* 1999, **54**(1-2):193-198.

176. Swartzrauber K, Vickrey BG, Mittman BS: **Physicians' Preferences for Specialty Involvement in the Care of Patients With Neurological Conditions.** *Med Care* 2002, **40**(12):1196-1209.

177. Teelucksingh S, Akong J, Klijn C, Ramdass M, Naraynsingh V: **Management of hyperthyroidism in Trinidad and Tobago.** *Int J Clin Pract* 2002, **56**(10):746-749.

178. Tracy CS, Dantas GC, Moineddin R, Upshur RE: **The nexus of evidence, context, and patient preferences in primary care: postal survey of Canadian family physicians.** *BMC Family Pract* 2003, **4**:13.

179. Vayda E, Mindell WR, Mueller CB: **Use of hypothetical cases to investigate indications for surgery.** *Can J Surg* 1981, **24**(1):19-21.

180. Vayda E, Mindell WR, Mueller CB, Yaffe B: **Measuring surgical decision-making with hypothetical cases.** *CMAJ* 1982, **127**(4):287-290.

181. Von Dem Knesebeck O, Gerstenberger E, Link C, Marceau L, Roland M, Campbell S, Siegrist J, De Cruppe W, McKinlay J: **Differences in the diagnosis and management of type 2 diabetes in 3 countries (US, UK, and Germany): Results from a factorial experiment.** *Med Care* 2010, **48**(4):321-326.

182. Votron L, D'Hoore W, Swine C, Daisne J-, Scalliet P: **The opinion of general practitioners on the treatment of prostate and breast cancer in elderly people: Results of a survey based on clinical models.** *Clin Oncol* 2004, **16**(7):474-478.

183. Weber EU, Bockenholt U, Hilton DJ, Wallace B: **Determinants of diagnostic hypothesis generation: effects of information, base rates, and experience.** *Journal of Experimental Psychology. Learning, Memory, and Cognition* 1993, **19**(5):1151-1164.

184. Wilson R, Godwin M, Seguin R, Burrows P, Caulfield P, Toffelmire E, Morton R, White P, Rogerson M, Eisele G, Bont G: **End-stage renal disease: Factors affecting referral decisions by family physicians in Canada, the United States, and Britain.** *Am J Kid Dis* 2001, **38**(1):42-48.

185. Young MJ, Fried LS, Eisenberg J, Hershey J, Williams S: **Do cardiologists have higher thresholds for recommending coronary arteriography than family physicians?** *Health Serv Res* 1987, **22**(5):623-635.
